# Conjugation-Induced Self-Selective Coordination Enables Organic Polymers with Low-Temperature Ammonium-Ion Storage

**DOI:** 10.34133/research.1160

**Published:** 2026-03-09

**Authors:** Xinji Zhou, Tiezhu Xu, Miaoran Zhang, Tengyu Yao, Zhenming Xu, Duo Chen, Laifa Shen

**Affiliations:** Jiangsu Key Laboratory of Electrochemical Energy Storage Technologies, College of Material Science and Technology, Nanjing University of Aeronautics and Astronautics, Nanjing 211106, China.

## Abstract

Aqueous ammonium-ion (NH_4_^+^) batteries/capacitors, recognized for inherent high safety and fast diffusion kinetics, are a promising alternative for sustainable energy storage. However, the development of ammonium-ion energy storage devices has been hindered by the poor compatibility between the distinctive solvation structure of NH_4_^+^ ions and conventional organic electrode materials, especially under low-temperature conditions. Here, a redox-active conjugated polymer with self-selective coordination mechanism is designed for achieving high-rate and low-temperature performance. The electron delocalization induced by the conjugated backbone facilitates rapid electronic transport in electrodes, delivering an ultrahigh-rate capacity of 107 mAh g^−1^ at 20 A g^−1^ under 25 °C and stable cycling performance with 99% capacity retention under −50 °C. Theoretical calculations and experimental investigations reveal that the inherent structural self-selectivity renders symmetrically arranged carbonyl groups as active binding sites for NH_4_^+^ storage, leading to a reversible 4-electron coordination process. Hence, the assembled all-organic hybrid ammonium-ion capacitor enables a long cycle life for over 3,000 cycles at −50 °C, which surpasses the lowest operating temperature reported for ammonium-ion devices, thus propelling the advancement of ammonium-ion energy storage technologies at low temperatures.

## Introduction

The growing demand for renewable energy sources has accelerated progress in new-generation energy storage technologies [1]. In recent decades, lithium-ion batteries (LIBs) have been recognized as a leading type of rechargeable system, owing to their high energy density, long cycle life, and minimal self-discharge, which make them highly suitable for diverse applications [2]. Nevertheless, the development of LIBs is substantially hindered by several factors, including the high cost of organic electrolytes, the inherent safety risks, the scarcity of lithium resources, and the pronounced degradation of capacity under low-temperature conditions [3,4]. Rechargeable batteries/capacitors using aqueous electrolytes occupy a distinctive position in energy storage technologies, especially in large-scale grid applications and compact power solutions for wearable devices, owing to cost-effectiveness, safe operation characteristics, and environmental benefits [5–8]. Conventionally, metallic cations, including Li^+^, Na^+^, K^+^, Zn^2+^, Mg^2+^, and Al^3+^ ions, are utilized as charge carriers in the majority of aqueous batteries/capacitors [9–21], but the sluggish solid-state diffusion process restricts further advancement [22]. In contrast, the increasing focus on non-metallic cations as charge carriers in aqueous batteries/capacitors is attributed to their rich availability, smaller hydrated ion size, and light molar mass [23,24]. Among them, acidic electrolytes based on proton (H^+^) typically exhibit a lower freezing point, which endows proton batteries/capacitors with great potential for cold climates [25–27]. However, despite the substantial progress made in H^+^-based proton batteries/capacitors, the strong acidic electrolytes can expedite the dissolution of electrode materials and current collectors, which unavoidably leads to cost losses and poses challenges for large-scale applications [28]. Fortunately, the ammonium salt electrolytes used in ammonium-ion batteries/capacitors undergo hydrolysis to form a mildly acidic or neutral environment, which effectively prevents corrosion of materials and devices while substantially suppressing side reactions. Moreover, unlike spherical metal charge carriers, the uniquely tetrahedral structure of NH_4_^+^ ion imparts distinct electrochemical behavior. In particular, the establishment of flexible hydrogen bonding interactions involving NH_4_^+^ ions and the host framework not only mitigates structural deformation of the electrodes but also reduces ion transport resistance, thereby enhancing reaction kinetics. Therefore, a comprehensive investigation into the electrochemical behavior and reaction mechanisms of NH_4_^+^ ions within electrode materials is essential to advancing efficient and practical ammonium-ion energy storage systems [29].

So far, extensive research has focused on exploring anode materials for ammonium-ion batteries/capacitors [30–32]. In particular, organic electrode materials are attracting growing interest due to their inherent sustainability, tunable molecular structure, minimal ion size constraints, and low cost [33]. However, organic small molecules and certain electronically insulating polymers often exhibit limited structural stability and tend to dissolve in the electrolyte, causing continuous capacity fading and poor cycling performance [34]. Consequently, exploring novel structural designs and improving interfacial compatibility are of paramount importance for enhancing the overall durability of organic-based electrode materials. Among all organic electrode materials, carbonyl derivatives stand out as the most prevalent organic anode/cathode materials for ammonium-ion batteries/capacitors [35]. Building upon this foundation, organic polymers containing rich carbonyl groups and π-conjugated structures have gained considerable attention owing to their exceptional electronic and ionic conductivity, highly tunable structures, and the potential for achieving large theoretical specific capacity [36,37]. It should be emphasized that the electrochemical performance and redox behavior in conjugated polymers are able to be precisely modulated through molecular design and electronic tuning techniques. However, the majority of these materials often contain a high proportion of inactive molecular units, which hinders charge transport and the redox kinetics of NH_4_^+^ storage [38,39].

Herein, we present a carbonyl-rich organic polymer with enhanced electronic conductivity by introducing active molecular units to construct an extended conjugated framework. The self-selective coordination induced by electronic conjugation enables a combined interaction between coordinated and synergistically coordinated carbonyl groups, which enhances NH_4_^+^ storage capability and facilitates coordination reaction kinetics. Benefiting from this structural advantage, the poly [(pentacene-bis (naphthalene diimide))-alt-(perylene diimide)] (PDP) electrode exhibits high-rate performance (107 mAh g^−1^ at 20 A g^−1^) and superior low-temperature performance (operating stably at −50 °C). Additionally, the polymer electrode can achieve a reversible 4-electron transfer during the coordination/de-coordination process with NH_4_^+^ ions, accompanied by the interconversion between C=O and C–O bonds. Meanwhile, PDP//PANI@rGO (a composite of polyaniline [PANI] and reduced graphene oxide [rGO]) all-organic hybrid ammonium-ion capacitors are assembled for the first instance, achieving an outstanding capacity retention of 97.5% after 3,000 cycles at −50 °C, breaking the minimum temperature limit for stable operation of ammonium-ion devices.

## Results and Discussion

### Theoretical prediction of NH_4_^+^ storage

**Fig. 1.**
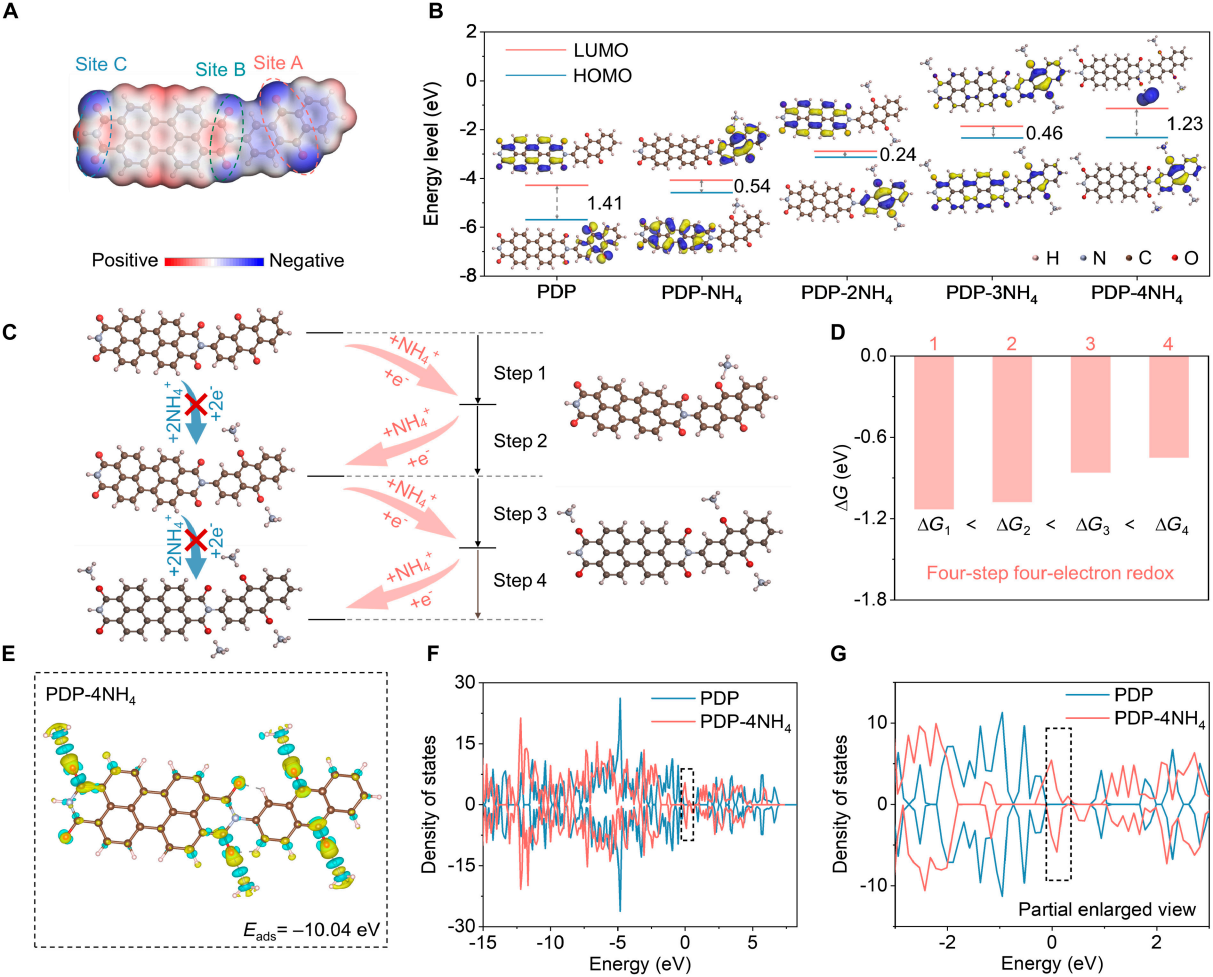
NH_4_^+^ storage in PDP predicted by theoretical analysis. (A) MESP distribution of PDP molecule. (B) Determined relative HOMO/LUMO energy gaps of PDP and PDP-xNH_4_ via the DFT method. (C) Structure evolution obtained from simulations of PDP under various NH_4_^+^ coordination routes. (D) Calculated ∆*G* of possible reaction paths. (E) Adsorption energy and charge density of PDP-4NH_4_. (F) DOS of PDP and PDP-4NH_4_. (G) Partial enlarged view of (F).

To evaluate the potential of PDP as an anode material for ammonium-ion batteries/capacitors, theoretical analyses based on density functional theory (DFT) calculations were performed. Molecular electrostatic potential (MESP) was employed to predict redox-active sites associated with electrophilic/nucleophilic interactions on the PDP electrode. As illustrated in Fig. 1A, the MESP image reveals pronounced regions of negative electrostatic potential (depicted in blue) surrounding the 6 oxygen atoms within each structural unit, indicative of high electronegativity and strong cation affinity. This suggests a marked tendency of C=O bonds to interact with NH_4_^+^ ions throughout the redox reaction. The C=O sites on the anthraquinone moiety are designated as Site A, while those on the perylene diimide moiety are referred to as Sites B and C. Notably, the electrostatic potential of Site A is more negative than that of Site C, which, in turn, is more negative than that of Site B. The more negative the electrostatic potential, the stronger the electron-withdrawing ability and the greater the affinity for NH_4_^+^ binding.

Through theoretical calculations, the possible reaction pathways and coordination mechanisms involved in the interaction between the PDP electrode and NH_4_^+^ ions were preliminarily predicted, providing theoretical insights into its electrochemical behavior. In accordance with molecular orbital theory, highly conjugated molecular frameworks tend to lower the energy levels of both the lowest unoccupied molecular orbital (LUMO) and the highest occupied molecular orbital (HOMO) [40,41]. As shown in Fig. 1B, pristine PDP exhibits the widest LUMO–HOMO gap (∆*E*) of 1.41 eV among all examined states. Upon coordination with NH_4_^+^ ion (denoted as PDP-xNH_4_), the energy gap narrows considerably, reflecting a lower barrier for electron excitation from HOMO to LUMO. Interestingly, when the number of coordinated NH_4_^+^ ion increases to 3 or 4, an unexpected widening of the energy gap is observed (Fig. S1). This phenomenon may result from the disruption of conjugation or electron cloud redistribution induced by the excessive presence of NH_4_^+^ ions, which in turn alters the molecular orbital energy gap. It can be observed that the LUMO in PDP-4NH_4_ is no longer distributed over the perylene diimide or anthraquinone planes, implying that [PDP]^4–^ anion can no longer accommodate additional electrons. The limitation in its electron-accepting ability determines that PDP may exhibit a 4-electron redox behavior during the electrochemical process.

In addition, the possible coordination pathways of NH_4_^+^ ion were elucidated from a thermodynamic perspective. By comparing the adsorption energies at different carbonyl sites, we identify the sequential binding sites of NH_4_^+^. Site 3 in region A (Fig. S2A) shows the most negative adsorption energy (−2.74 eV), corresponding to the strongest electrostatic attraction and thus serving as the initial binding site. Considering coordination-induced electronic structure rearrangement, subsequent calculations reveal that another site in region A (Fig. S2B) with a more negative adsorption energy (−5.56 eV) functions as the second coordination site. After the coordination of 2 NH_4_^+^ ions, further calculations assign the third coordination site to site 3 in region C (Fig. S2C) and the fourth to site 1 in region B (Fig. 1E and Fig. S2D), the latter exhibiting an adsorption energy of −10.04 eV. Notably, another site in region B (site 3) shows a relatively less negative adsorption energy, which can be attributed to steric hindrance effects. Moreover, considering the crowded distribution of active sites on PDP and the electrostatic repulsion between adjacent NH_4_^+^ ions, only 2 carbonyl groups located on diagonally opposite positions of the perylene diimide ring participate in NH_4_^+^ coordination [31,42,43]. Therefore, PDP molecule exhibits self-selective coordination, in which the active carbonyl groups participate in redox reactions for NH_4_^+^ storage, while the synergistically coordinated carbonyl groups modulate polar interactions to enable rapid NH_4_^+^ transport. Figure 1C and Fig. S4 clearly demonstrate this 4-step sequential NH_4_^+^ coordination process during discharge. Concurrently, the Gibbs free energy change (Δ*G*) (Fig. 1D and Fig. S3) shows that the values for Steps 1 to 4 follow the trend Δ*G*_1_ < Δ*G*_2_ < Δ*G*_3_ < Δ*G*_4_, indicating that these 4 steps can proceed sequentially in a thermodynamically favorable manner. Therefore, based on DFT calculations and predictions, NH_4_^+^ ions may undergo a 4-step, 4-electron coordination reaction on the PDP electrode.

Figure 1F shows the density of states (DOS) of PDP and PDP-4NH_4_. In the partial enlarged view (Fig. 1G), it is clearly observed that PDP-4NH_4_ exhibits an increased DOS at the Fermi level relative to PDP, further highlighting its ability to facilitate quick charge transport. The electron localization function (ELF) analysis (Fig. S5) was performed for the PDP and PDP-4NH_4_. Prior to and after NH_4_^+^ coordination, the molecular backbone exhibits distinct red petal-shaped ELF distributions, suggesting strong electron localization within covalent frameworks such as C–C, C=C, and C=O bonds [27]. The alteration in the electron delocalization distribution of PDP upon binding with NH_4_^+^ directly demonstrates that their coordination interaction occurs through localized electron interaction.

### Synthesis and structural characterization

**Fig. 2.**
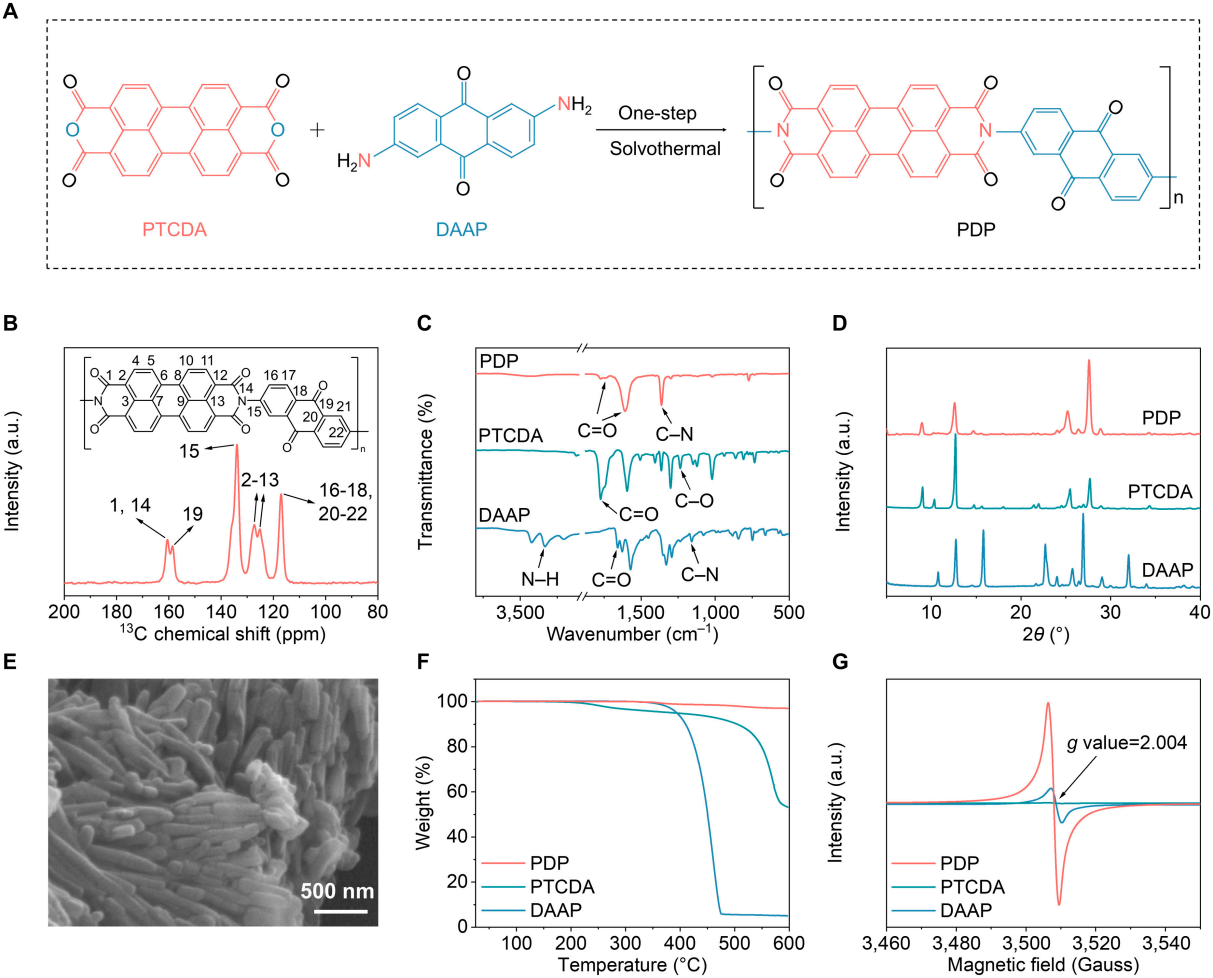
Synthesis and physicochemical characterization of PDP. (A) Schematic illustration of the preparation process of PDP polymer. (B) ^13^C NMR spectrum of PDP polymer. (C) FTIR spectra, (D) XRD patterns of PDP polymer and 2 monomers. (E) SEM image of PDP polymer. (F) TGA curves and (G) EPR spectra of PDP polymer and 2 monomers.

The PDP polymer was synthesized via a straightforward one-step solvothermal reaction using perylene-3,4,9,10-tetracarboxylic dianhydride (PTCDA) and 2,6-diaminoanthraquinone (DAAP) as organic monomers. This synthetic strategy features a simplified reaction pathway and controllable product morphology, along with the use of low-cost, readily available monomers and recyclable solvents, demonstrating preliminary potential for industrial scale-up. The formation of the amide structure enables the construction of an extended conjugated backbone and simultaneously provides additional reactive sites (Fig. 2A). As illustrated in Fig. S6, both the HOMO and LUMO energy levels of PDP lie between those of the 2 monomers, showing the smallest HOMO–LUMO gap among them. This is primarily attributed to the relatively high molecular polarizability of PDP, which supports its outstanding conductivity and promotes fast electron transfer. Solid-state ^13^C nuclear magnetic resonance (NMR) spectrum of PDP (Fig. 2B) retains the characteristic aromatic carbon signals of both the perylene unit in PTCDA and the anthraquinone unit in DAAP (Fig. S7B and C) in the range of 100 to 150 parts per million (ppm), confirming that the conjugated frameworks of both monomers remain intact and are fully incorporated into the polymer backbone. In the carbonyl region, the anhydride C=O resonances of PTCDA (160 to 200 ppm) shift to higher field (150 to 170 ppm) in PDP. This shift stems from imidization between the anhydride groups of PTCDA and the amino groups of DAAP, where the electron-donating resonance effect of the nitrogen increases carbonyl electron density and reduces deshielding. Further evidence for this structural transformation comes from ^1^H NMR spectra. DAAP displays high-field aromatic proton signals (6.3 ppm) due to the electron-donating effect [44], whereas PTCDA gives downfield signals at 8.3 ppm dominated by π-deshielding. Following imidization in PDP, the removal of amino groups shifts the aromatic protons to an intermediate position at 7.8 ppm (Fig. S7A, D, and E).

In the Fourier transform infrared (FTIR) spectrum (Fig. 2C), PTCDA is characterized by C=O and C–O stretching peaks at 1,771 and 1,236 cm^−1^, while DAAP presents C–N, N–H, and C=O peaks at 1,157, 3,332, and 1,657 cm^−1^, respectively. For PDP, new C=O (1,743 and 1,608 cm^−1^) and C–N (1,361 cm^−1^) peaks emerge, accompanied by the disappearance of the C–O signal from PTCDA, collectively pointing to successful imidization. Raman spectroscopy (Fig. S8) offers additional confirmation of the successful synthesis by revealing a C–N vibration at 1,287 cm^−1^ and the lack of C–O stretching. The characteristic peaks observed in the x-ray diffraction (XRD) patterns mainly reflect local ordering between molecules or chain segments. Differences in peak positions and intensities between the monomers and the polymer reflect changes in molecular packing and ordering (Fig. 2D). Powder wide-angle x-ray scattering (WAXS) is employed to assess molecular ordering in the bulk state. The 2-dimensional WAXS pattern shows continuous diffraction rings (Fig. S9), indicating the presence of ordered domains and semicrystalline packing. A diffraction ring in the high-*q* region is commonly associated with π–π stacking-related intermolecular correlations [45].

As seen in the scanning electron microscopy (SEM) image (Fig. 2E), PDP polymer forms numerous well-ordered nanorods about 500 nm in length. Thermogravimetric analysis (TGA) (Fig. 2F) confirms excellent thermal stability of PDP polymer, with a mass loss of less than 3% at 600 °C, whereas its monomer suffered severe decomposition. Moreover, the electron paramagnetic resonance (EPR) spectra (Fig. 2G) corroborate the successful synthesis of PDP at the electronic-structure level. While PTCDA shows no detectable signal and DAAP shows only a weak one, PDP produces a strong Gaussian signal at 3,508.7 (*g* = 2.004). This signal, indicative of spin-delocalized unpaired radicals that facilitate rapid ion/electron transfer [46], originates not from the monomers but from the polymerization-induced reconstruction of molecular conjugation. The reaction disrupts the paired-electron state of the monomers, forming a stable organic radical structure. Finally, contact angle measurements (Fig. S10) indicate that the PDP polymer possesses a smaller wettability angle compared to the PTCDA and DAAP monomers, suggesting good interfacial compatibility between the PDP material and the electrolyte.

### Electrochemical behavior of NH_4_^+^ versus other metal cations

**Fig. 3.**
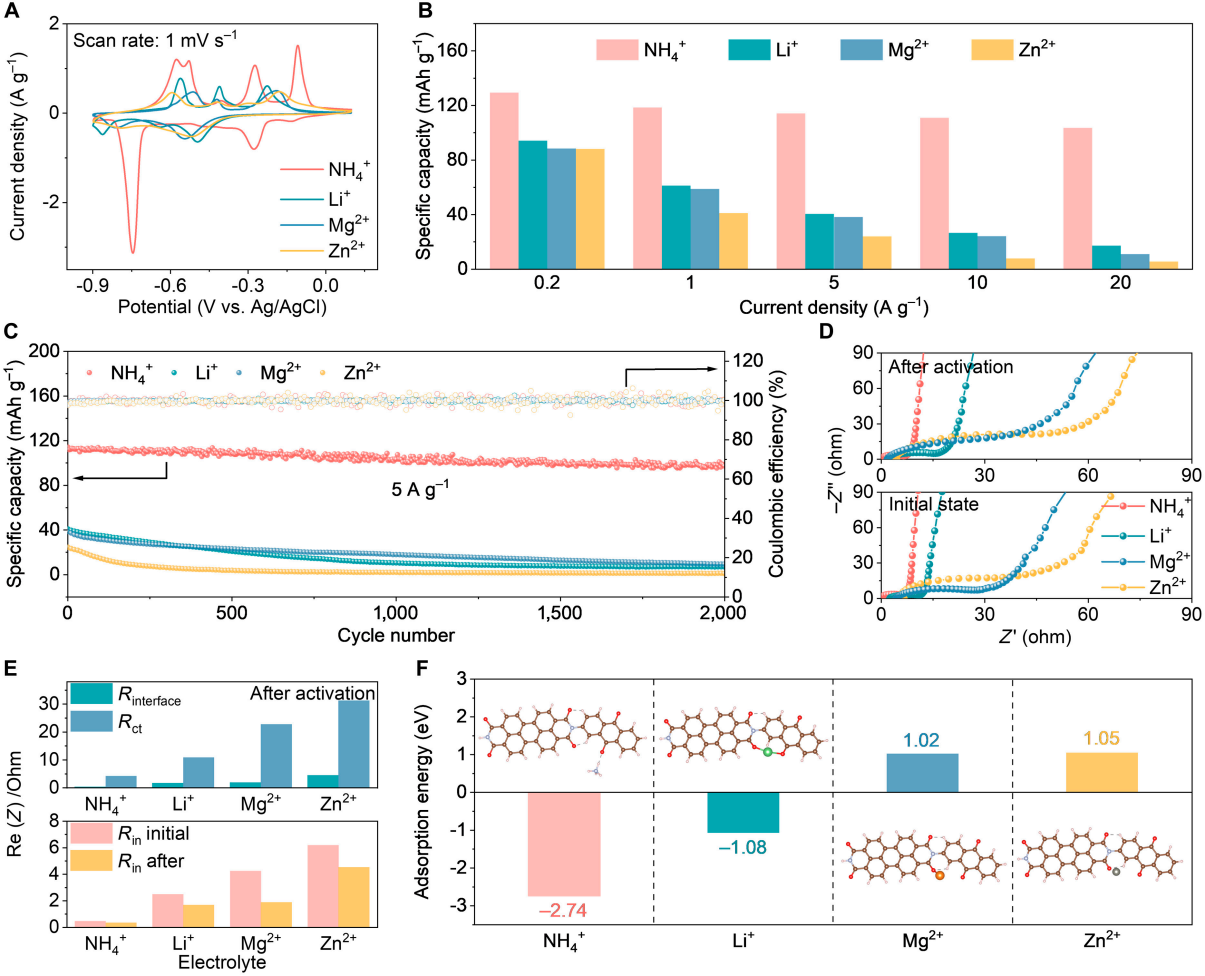
Electrochemical performance and adsorption energy analysis of PDP in a range of electrolytes containing cations. (A) CV curves in various cation-based electrolytes at a scan rate of 1 mV s^−1^. (B) Comparison of specific capacities for PDP electrode at various current densities in different electrolytes. (C) Cycling performance of PDP electrode at 5 A g^−1^ in different electrolytes. (D) Nyquist plots of PDP electrode in 4 distinct electrolytes before and after electrochemical activation. (E) The corresponding impedance derived from the equivalent circuit. (F) Comparison of cation adsorption energies within single-coordination structures.

Carbonyl-based organic materials feature abundant and accessible carbonyl groups that serve as coordination sites for reversible cation binding, making them broadly applicable to a variety of charge carriers (e.g., H^+^, Li^+^, Na^+^, K^+^, Zn^2+^, Mg^2+^, and Ca^2+^) [47]. Given this, we investigated the electrochemical behavior of PDP electrodes toward both non-metallic (NH_4_^+^) and metallic cations (Li^+^, Mg^2+^, and Zn^2+^). All tests were carried out in a standard 3-electrode system using 0.5 M (NH_4_)_2_SO_4_, 0.5 M Li_2_SO_4_, 1 M MgSO_4_, and 1 M ZnSO_4_ electrolytes, with active-material mass loadings ranging from 0.8 to 1.5 mg cm^−2^. Cyclic voltammetry (CV) curves in different electrolytes are presented in Fig. 3A and Fig. S11. Notably, the peak currents in the NH_4_^+^ electrolyte are relatively higher, reflecting a stronger redox current response and faster reaction kinetics. To provide electrochemical evidence for PDP synthesis, CV profiles of PTCDA and DAAP were obtained in different electrolytes at 1 mV s^−1^. In the NH_4_^+^ electrolyte (Fig. S13A), PTCDA features 3 reduction peaks at −0.43, −0.71, and −0.76 V, while DAAP presents a single peak at −0.26 V. PDP displays 3 reduction peaks at −0.13, −0.28, and −0.72 V, where the former 2 correspond to DAAP and the latter to PTCDA, confirming that PDP retains the C=O redox-active sites of both monomers. Furthermore, the CV profiles of PDP in Li^+^, Mg^2+^, and Zn^2+^ electrolytes (Fig. S13B to D) differ markedly from those of PTCDA, highlighting distinct redox behavior.

The specific capacities of the PDP electrode across the various cation-containing electrolytes are summarized in Fig. 3B and Fig. S12. It is obvious that PDP delivers a specific capacity of 126.9 mAh g^−1^ at 0.2 A g^−1^ and 107 mAh g^−1^ at 20 A g^−1^ in the NH_4_^+^ electrolyte, achieving an impressive capacity retention of 84.4%. In contrast, PTCDA suffers severe capacity decay (Fig. S13E) with increasing current density. Compared with most organic and inorganic materials studied for ammonium-ion batteries/capacitors (Table S1), PDP achieves outstanding rate performance for NH_4_^+^ storage. Moreover, the PDP electrode exhibits remarkable cycling stability in the NH_4_^+^ electrolyte with a capacity retention of 85.2% after 2,000 cycles (Fig. 3C). By comparison, significant capacity fading is observed in other electrolytes. As seen in Fig. S14, only the NH_4_^+^ electrolyte remains clear after cycling, while the others turn yellow, suggesting electrode dissolution. Electrochemical impedance spectroscopy (EIS) analysis (Fig. 3D and E) indicates that PDP possesses the lowest internal (*R*_in_) and charge transfer resistances (*R*_ct_) in the NH_4_^+^ electrolyte, both before and after activation (Figs. S15 and S16), pointing to the fastest interfacial kinetics. This result is ascribed to hydrogen-bond-assisted coordination between NH_4_^+^ ions and carbonyl groups, which facilitates the charge-transfer process.

To clarify the origin of PDP’s outstanding electrochemical performance in the NH_4_^+^ electrolyte, DFT calculations were performed to determine the adsorption energy of NH_4_^+^ on the electrode surface. As illustrated in Fig. 3F and Fig. S17, the adsorption energies of NH_4_^+^ are more negative than those of Li^+^, Mg^2+^, and Zn^2+^ across all evaluated sites. This implies that NH_4_^+^ forms stronger binding interactions with the coordination sites of the PDP molecule, and this advantage is universal across sites rather than being confined to specific active sites. The underlying cause of this differentiated adsorption behavior lies in the solvation behavior of different cations. NH_4_^+^ adopts a tetrahedral configuration, forming a loose NH_4_^+^(H_2_O)_4_ solvation structure through weak hydrogen bonds with 4 H_2_O molecules, with bond lengths (Fig. S18A) ranging from 1.76 to 1.81 Å and a binding energy (Fig. S18B) of only −1.35 eV. In comparison, metal cations form dense $M^{n+}$(H_2_O)_6_ structures through strong coordination bonds and ion-dipole interactions with 6 H_2_O molecules, exhibiting bond lengths greater than 2.0 Å and more negative binding energies. The differences in solvation configuration, coordination number, and interaction strength lead to lower energy consumption for the desolvation of NH_4_^+^, resulting in more rapid coordination reactions at the electrode interface. In summary, the PDP polymer can flexibly accommodate various cations, but display the best rate performance and cycling stability in the NH_4_^+^ electrolyte, reflecting superior compatibility with NH_4_^+^ ions.

### Ammonium-ion storage mechanism

**Fig. 4.**
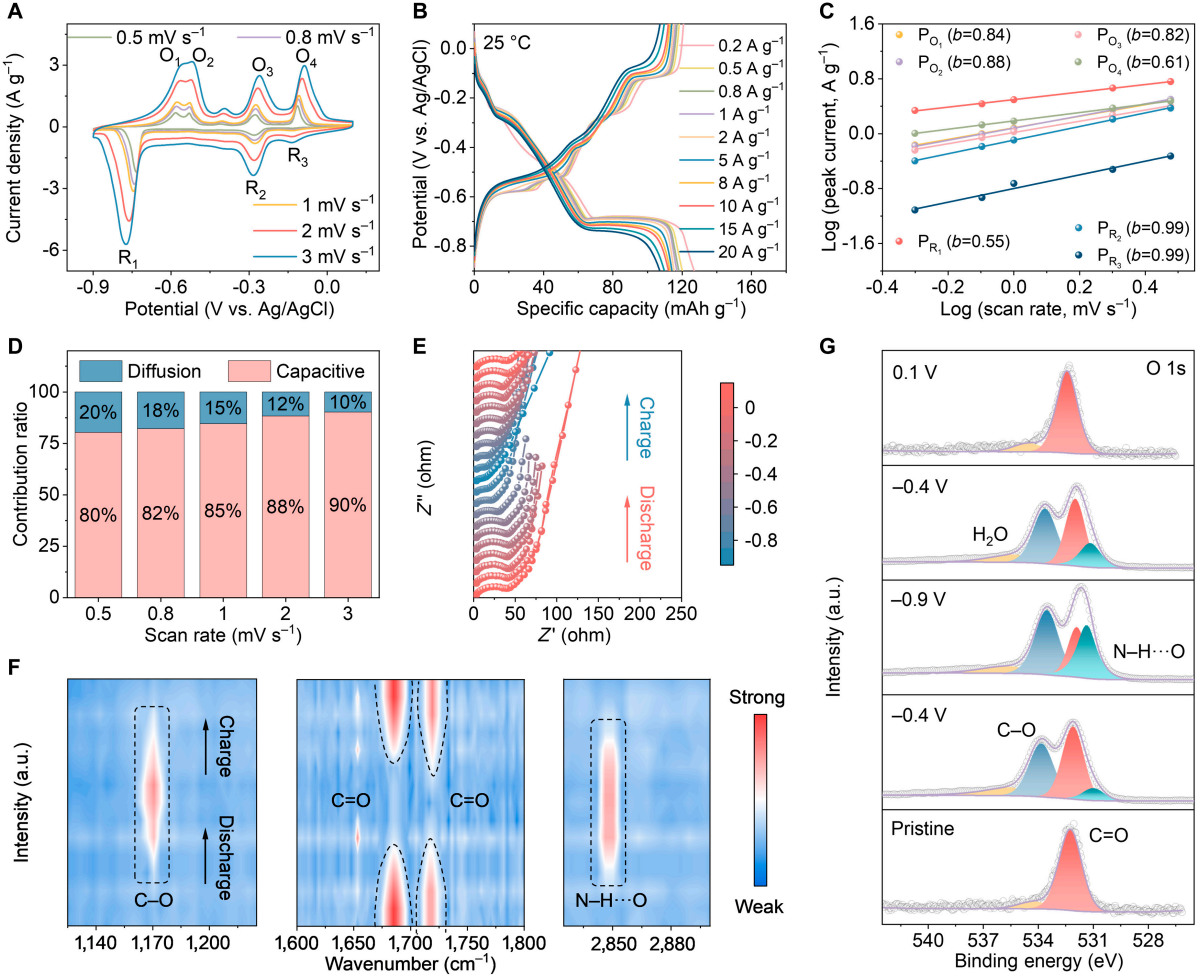
Reaction kinetic analysis and spectroscopic characterization of NH_4_^+^ storage mechanism in PDP. (A) CV curves of PDP electrode at various scan rates (25 °C). (B) GCD curves of PDP electrode at various current densities (25 °C). (C) *b* values extracted from Log (*i*) − Log (*v*) diagram. (D) Capacitive and diffusion-controlled capacity ratios at various scan rates. (E) In situ EIS plot of PDP electrode. (F) In situ FTIR spectra of PDP electrode. (G) Ex situ XPS spectra of O 1s.

CV tests were conducted at scan rates of 0.5, 0.8, 1, 2, and 3 mV s^−1^ (Fig. 4A). Figure 4B presents the galvanostatic charge and discharge (GCD) curves of the PDP electrode across current densities from 0.2 A g^−1^ to 20 A g^−1^ at 25 °C, which maintain similar profiles even at high rates. The corresponding differential capacitance (*dQ*/*dV*) curve in Fig. S19B was derived from the third cycle of GCD curve at 0.2 A g^−1^ (Fig. S19A). The curve displays 4 distinct pairs of redox peaks (at −0.11/−0.12 V, −0.26/−0.27 V, −0.51/−0.49 V, and −0.56/−0.70 V), effectively resolving redox signals that are less distinct in the CV profile. This visually confirms that the interaction of NH_4_^+^ with the PDP electrode involves 4 independent and highly reversible redox steps, with the carbonyl activity of perylene diimide and anthraquinone monomers fully preserved during polymerization. Furthermore, the *b* values of the redox peaks, obtained from the power-law relationship *i* = *av^b^* [48], fall between 0.5 and 1 (Fig. 4C), suggesting that the charge/discharge reactions of PDP electrode are governed by both diffusion and surface capacitance effects. As depicted in Fig. 4D, the capacitive contribution ratio rises with the scan rate, implying that capacitance effects dominate at higher scan rates. In situ EIS analyses were performed to examine the electrode reaction kinetics of PDP during the charge and discharge process. The potential-time profile recorded during the EIS measurement is shown in Fig. S20. Figure 4E reveals that the *R*_ct_ of PDP electrode decreases during discharge, attributed to the formation of stable O⋯H–N hydrogen bonds between C=O groups and NH_4_^+^ ions, which facilitate interfacial electron transport and charge transfer. Upon charging, hydrogen-bond dissociation weakens the interfacial interactions, leading to an increased *R*_ct_ and recovery of the initial surface state of the electrode.

To gain deeper insight into the NH_4_^+^ storage mechanism, multiple spectroscopic techniques were employed to probe the interaction between PDP electrode and NH_4_^+^ ions at different potentials. In situ FTIR and Raman spectroscopy monitored the evolution of functional groups in the PDP electrode during charge/discharge (Fig. 4F and Fig. S23). The corresponding GCD profile is provided in Fig. S21. During discharge, the anthraquinone C=O vibration gradually weakens and diminishes first, while the perylene imide C=O signal remains initially unchanged and subsequently attenuates with deeper discharge, a sequential redox process consistent across both in situ FTIR and Raman spectra. Notably, the perylene imide carbonyl signal does not vanish completely, suggesting that only part of these sites participate in the reaction. Simultaneously, the emergence and progressive intensification of C–O and N–H···O vibrational bonds confirm coordination between carbonyl groups and NH_4_^+^ ions. Slight peak shifts in the Raman spectrum further indicate coordination-induced electronic redistribution and structural relaxation [49]. Upon charging, the C=O bonds recover in reverse order, accompanied by the weakening and disappearance of C–O and N–H signals, demonstrating the reversibility of the process. In summary, in situ FTIR and Raman characterization reveals 3 key points: (a) C=O groups are identified as the redox-active sites; (b) a sequential reaction order exists, with anthraquinone carbonyls reacting before perylene imide carbonyls; and (c) not all 4 carbonyl groups on the perylene imide ring participate in the reaction. To directly correlate the potential with functional group evolution, spectra were extracted from the continuously acquired in situ FTIR data at the initial potential and at 4 reduction potentials (Fig. S22) corresponding to the discharge features on the *dQ*/*dV* curve. The results reveal that upon discharge to −0.12 and −0.27 V, the anthraquinone carbonyl peak gradually attenuates and nearly diminishes, corresponding to the stepwise coordination of 2 carbonyl groups with NH_4_^+^ ions, each accompanied by the transfer of one electron. As the discharge proceeds to −0.49 and −0.70 V, the perylene diimide carbonyl peak progressively weakens, reflecting the involvement of 2 additional carbonyl groups in coordination and electron transfer. Notably, even at −0.70 V, the perylene diimide carbonyl signal does not disappear completely, suggesting that a fraction of carbonyl groups within this structural unit remains unreacted. Collectively, these observations assign the 4 reduction peaks to 4 distinct C=O sites (2 in the anthraquinone unit and 2 in the perylene diimide unit) that undergo successive coordination reactions, each coupled with a single-electron transfer, thereby constituting an overall 4-electron process.

Additionally, ex situ XPS of O 1s (Fig. 4G) was used to analyze the PDP electrode from the pristine state to full charge. In the pristine electrode, the O 1s spectrum features 2 peaks at 532.1 and 534.5 eV, corresponding to C=O and adsorbed H_2_O, respectively. During discharge, coordination between the C=O groups and NH_4_^+^ ions leads to the gradual emergence of C–O peak at 533.8 eV and N–H···O hydrogen-bonding feature at 531.1 eV, both of which increase in intensity with deeper discharge. It is notable that the C=O peak does not disappear completely even at the lowest discharge potential, indicating the presence of uncoordinated carbonyl groups within the PDP electrode. Upon charging, the intensities of the C–O and N–H···O peaks gradually diminish and eventually disappear. In addition, the binding-energy shift of the C=O peak during cycling originates from hydrogen-bond formation with NH_4_^+^. The lone-pair electrons on the carbonyl oxygen gain partial electron density from the hydrogen-bond donor (NH_4_^+^), slightly increasing the core electron density and shifting the O 1s binding energy toward lower values by typically 0.2 to 0.5 eV. Such hydrogen-bond-induced shifts are commonly observed in carbonyl-based electrode materials [50], which further confirms the C=O bond as the active site for NH_4_^+^ storage and reflects the dynamic reconstruction of the hydrogen-bonding network at the electrode-electrolyte interface.

### Low-temperature performance of PDP in EG60 electrolyte

**Fig. 5.**
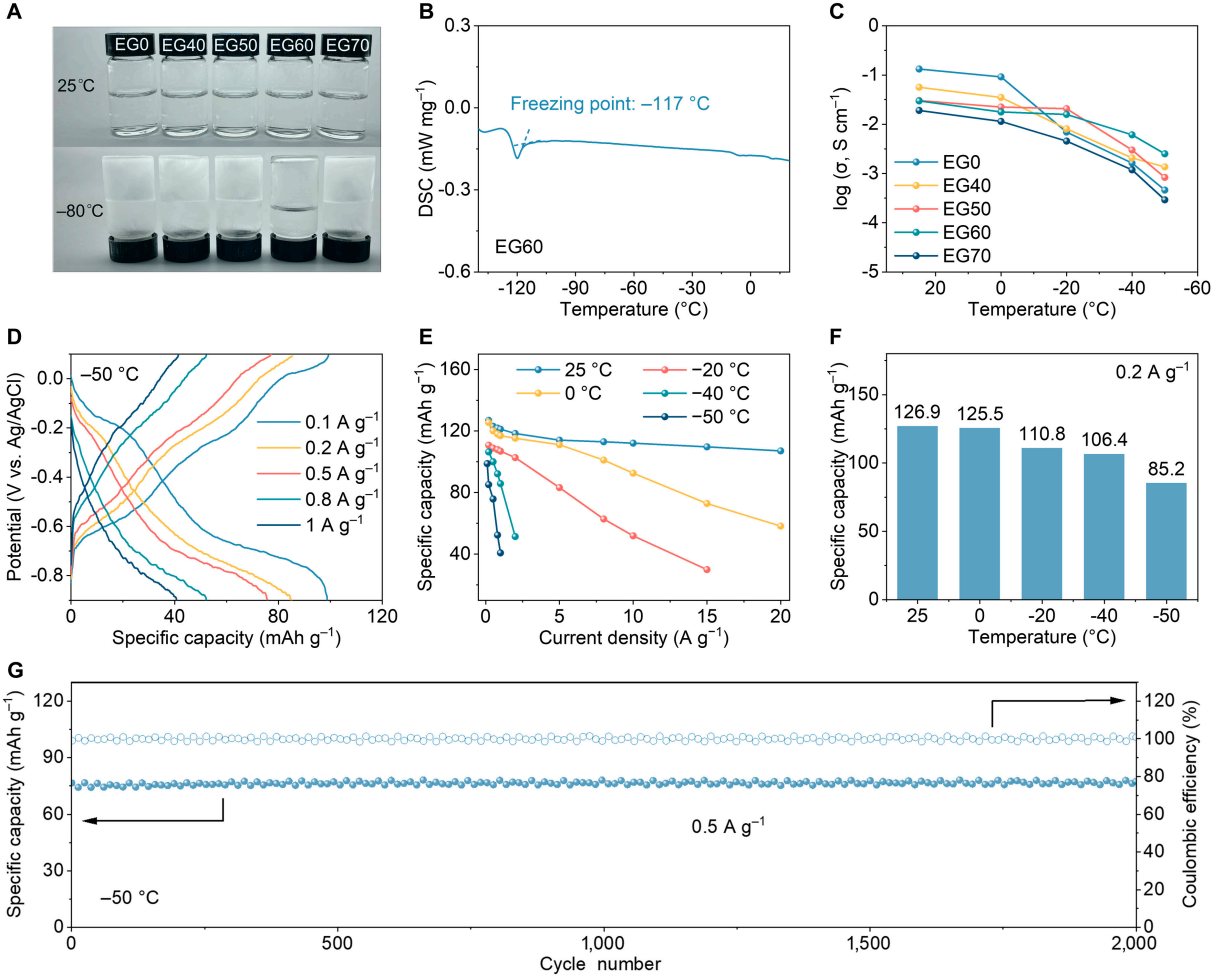
Design of low-temperature resistant electrolytes and electrochemical performance of PDP in EG60 electrolyte at different temperatures. (A) The digital photographs of electrolytes with different composition ratios at 25 and −80 °C. (B) DSC curve of EG60 electrolyte. (C) Temperature dependence of ion conductivity in different electrolytes. (D) GCD curves of PDP electrode at −50 °C. (E) Specific discharge capacities at various current densities and temperatures. (F) Comparison of specific capacities for PDP electrode at 0.2 A g^−1^ under different temperatures. (G) Cycling performance of PDP electrode at 0.5 A g^−1^ under −50 °C.

It is well established that optimizing electrolyte design is crucial for expanding the operating temperature range of energy storage devices. To enhance the electrolyte’s low-temperature performance, ethylene glycol (EG) was introduced as an additive [51]. A series of 0.5 M (NH_4_)_2_SO_4_ mixed electrolytes were prepared by dissolving ammonium sulfate in EG/H_2_O mixtures with volume ratios of 0:1, 4:6, 5:5, 6:4, and 7:3, designated as EG0, EG40, EG50, EG60, and EG70, respectively. As seen in Fig. 5A, all 5 electrolytes are colorless transparent liquids at 25 °C. After freezing at −80 °C for 24 h, only EG60 remained a flowing liquid, highlighting its superior low-temperature fluidity. Differential scanning calorimetry (DSC) was used to determine the freezing points of the electrolytes. EG60 possesses the lowest freezing point of −117 °C (Fig. 5B), compared with −7 °C for the EG-free electrolyte (Fig. S24). The freezing points of EG40, EG50, and EG70 were measured at −40, −42, and −41 °C, respectively.

To elucidate the synergistic interaction between EG and H_2_O, hydrogen-bond binding energy calculations (Fig. S25A) were conducted. The H_2_O–EG system exhibits a lower binding energy than H_2_O–H_2_O, suggesting stronger EG–H_2_O hydrogen bonding that disrupts the native water network. Raman spectra (Fig. S25B and C) display a gradual red shift of the O–H stretching band (3,000 to 3,600 cm^−1^) and enhanced, red-shifted C–H vibrations of EG (2,900 to 3,000 cm^−1^) with increasing EG content, confirming the formation of strong EG–H_2_O hydrogen bonds. Consistently, ^1^H NMR spectra (Fig. S25D) feature a linear downfield shift of the ^1^H signal with increasing EG ratio, reflecting enhanced mixed hydrogen bonding [52]. Furthermore, EIS measurements demonstrate that ionic conductivity decreases with temperature due to increased viscosity (Fig. S26), yet EG60 maintains higher conductivity than EG70 at low temperatures (Fig. S27 and Fig. 5C). Notably, EG60 retains a conductivity of 3.16 mS cm^−1^ at −50 °C, implying more efficient NH_4_^+^ transport within the EG–H_2_O hydrogen-bond network. Therefore, EG60 was selected as the optimal electrolyte. Variable-temperature Raman spectra (Fig. S25e) reveal negligible changes in O–H and C–H vibrational features as the temperature decreases, confirming a stable, homogeneous liquid/supercooled-liquid structure without phase separation, which ensures continuous ion transport at low temperatures.

To gain deeper insight into the regulatory mechanism of EG on NH_4_^+^ transport and stability, molecular dynamics (MD) simulations were performed. Structural snapshots (Fig. S28) illustrate that in EG60, NH_4_^+^ forms solvation clusters coordinated with EG, replacing the dense H_2_O-dominated network in EG0. The total number of hydrogen bonds decreases with increasing EG content, indicating reconstruction and moderate weakening of the water hydrogen-bond network, which reduces solvation constraints on NH_4_^+^ and enhances mobility. Radial distribution function and coordination analyses at −50 °C (Fig. S29) show reduced NH_4_^+^–H_2_O coordination and the emergence of NH_4_^+^–EG interactions at ~2 Å, together with slightly enhanced NH_4_^+^–SO_4_^2–^ interactions. This cooperative NH_4_^+^–EG–H_2_O solvation structure promotes ion migration while suppressing aggregation. Finally, energy barrier analysis (Fig. S30) reveals that introducing EG decreases the NH_4_^+^ desolvation energy from 105.70 to 87.98 kJ mol^−1^. Preferential removal of EG during initial desolvation facilitates coordination of NH_4_^+^ with PDP at the electrode–electrolyte interface. The temperature-dependent EIS measurement was conducted to analyze the electrode/electrolyte interfacial kinetics (Fig. S31). Compared with EG0, the PDP electrode in the EG60 electrolyte exhibits consistently lower *R*_ct_ across all measured temperatures, along with a lower activation energy (*E*_a_) derived from Arrhenius fitting (0.153 eV vs. 0.186 eV for EG0). Given that the active sites in PDP electrode are carbonyl groups, the charge storage mechanism involves the coordination of NH_4_^+^ ions with C=O groups at the interface accompanied by electron transfer. Therefore, the obtained *E*_a_ can be regarded as the energy barrier associated with the coupled process of NH_4_^+^ coordination and charge transfer process. The lower *E*_a_ signifies that NH_4_^+^ ions can interact more rapidly with the active sites, thereby enhancing the interfacial reaction kinetics. After 100 cycles, Raman analysis (Fig. S32) shows negligible change in the C=O peak intensity for PDP in EG60, whereas a pronounced decrease is observed in EG0, indicating that EG stabilizes carbonyl active sites by regulating interfacial solvation/desolvation and suppressing water-related competitive processes.

To evaluate the electrochemical performance of PDP electrode at low temperatures, tests were conducted at 0, −20, −40, and −50 °C. With decreasing temperature, pronounced polarization was observed in the CV curves (Fig. S33). Figure S34 and Fig. 5D present the GCD curves of PDP electrode under low-temperature conditions. Even at −50 °C, it retains a specific discharge capacity of 85.2 mAh g^−1^ at 0.2 A g^−1^, achieving 67.1% of its room-temperature capacity (Fig. 5E and F). To probe the role of self-selective coordination in structural stabilization, in situ Raman spectroscopy was further employed under low-temperature conditions (Fig. S35). The evolution of the C=O and C–O bonds follows trends similar to those at room temperature, suggesting that the underlying reaction pathway remains consistent. However, reduced dynamic coordination interactions between NH_4_^+^ ions and carbonyl groups at low temperature limit electron density redistribution, resulting in decreased peak intensities and vanishing peak shifts.

To clarify the role of the conjugated backbone, a non-conjugated polymer (poly [4,4'-diaminobenzophenone], PDABP) was used as a control (Fig. S36). PDP possesses a planar, fully conjugated main chain that enables efficient π-electron delocalization for charge transport and storage, whereas the sp^3^-hybridized amino linkages in PDABP disrupt long-range conjugation, causing charge storage to rely primarily on weak polar interactions and leading to inferior specific capacity and rate performance [53]. To elucidate the influence of the conjugated structure on the reaction kinetics of NH_4_^+^ ions at the electrode surface, the NH_4_^+^ diffusion coefficients ($D_{{{\mathrm{NH}}_{4}}^{+}}$) of conjugated PDP and non-conjugated PDABP were compared at different temperatures using the galvanostatic intermittent titration technique (GITT) (Fig. S37). The results indicate that at 25 °C, PDP exhibits the $D_{{{\mathrm{NH}}_{4}}^{+}}$ of $10^{-10.59}$ to $10^{-9.02}$ cm^2^ s^−1^, markedly higher than that of PDABP ($10^{-11.11}$ to $10^{-9.86}$ cm^2^ s^−1^). At 0 °C, the $D_{{{\mathrm{NH}}_{4}}^{+}}$ of PDP decreases only slightly to $10^{-11.05}$ to $10^{-9.06}$ cm^2^ s^−1^, whereas PDABP suffers a pronounced decline of more than one order of magnitude. At even lower temperatures (−20 and −40 °C), PDP still maintains effective NH_4_^+^ storage capability, while PDABP can no longer operate. These findings clearly demonstrate that the conjugated structure stabilizes surface coordination, which mitigates the impact of low temperature on the coordination reaction between NH_4_^+^ ions and C=O groups, thereby enhancing the interfacial reaction kinetics.

As shown in Fig. S38, the charge-transfer resistance of the PDP electrode increases as the temperature decreases, leading to capacity degradation. Nevertheless, the electrode achieves a capacity retention of 97.1% after 2,000 cycles at 1 A g^−1^ under −40 °C (Fig. S39), and even a capacity retention of 99% after 2,000 cycles at 0.5 A g^−1^ under −50 °C (Fig. 5G). SEM and XPS characterizations were performed on the PDP electrode before and after cycling. The SEM results (Fig. S40) reveal that the electrode retains its well-ordered nanorod morphology even after 2,000 cycles without noticeable structural expansion, indicating excellent morphological stability. Further insights from the O 1s XPS spectra show that prior to cycling, the O 1s signal is dominated by C=O bonds (86.9%) with a minor contribution from adsorbed water (13.1%) (Fig. S41A and C). After cycling, the C=O component remains predominant (79.1%), accompanied by a small fraction of C–O bonds (6.4%) (Fig. S41B and D), which can be attributed to slight physical adsorption of the electrolyte rather than decomposition products. Notably, no characteristic peaks associated with side reactions were detected. These results collectively demonstrate that the PDP electrode undergoes negligible side reactions or interfacial degradation during cycling, exhibiting its outstanding structural stability. Additionally, electrochemical performance tests were conducted at varying temperatures on 3,4,9,10-perylenetetracarboxylic diimide (PTCDI) and polyimide (PI) (Fig. S42), both of which feature imide structures. Compared with PTCDI and PI, PDP demonstrates markedly superior rate performance, low-temperature performance, and cycling stability across all tested conditions. While PTCDI and PI suffer severe capacity loss and rapid failure at low temperatures, PDP maintains high capacity retention even at −20 °C and after long-term cycling. These results highlight that polymerization mitigates the dissolution of small molecules and that the extended conjugated backbone of PDP enables fast electron transport, accounting for its outstanding electrochemical performance.

### Electrochemical performance of hybrid ammonium-ion capacitor

The superior electrochemical performance of the PDP electrode motivated us to explore its application in ammonium-ion devices. Taking advantage of the matched redox potentials, PANI@rGO was selected as the cathode material to construct an all-organic hybrid ammonium-ion capacitor. On the one hand, the combination of nanoparticulate PANI and nanosheet-shaped rGO yields a porous, interconnected network (Fig. S43) that facilitates rapid ion diffusion and efficient charge transfer. Compared with the FTIR spectrum of rGO, the new peaks at 1,652, 1,476, 1,294, and 795 cm^−1^ are attributed to C=N, C=C, C–N, and C–H vibrations, respectively (Fig. S44), confirming the successful coupling of PANI with rGO. On the other hand, oligomers of aniline with terminal hydroxyl or amine groups, arising from PANI degradation, serve as additional electroactive materials that enhance capacity, while rGO acts as a conductive matrix that stabilizes the degraded PANI, thereby improving overall conductivity. Consequently, the PANI@rGO composite displays substantially enhanced rate performance and cycling stability (Figs. S45 and S46) [54].

**Fig. 6.**
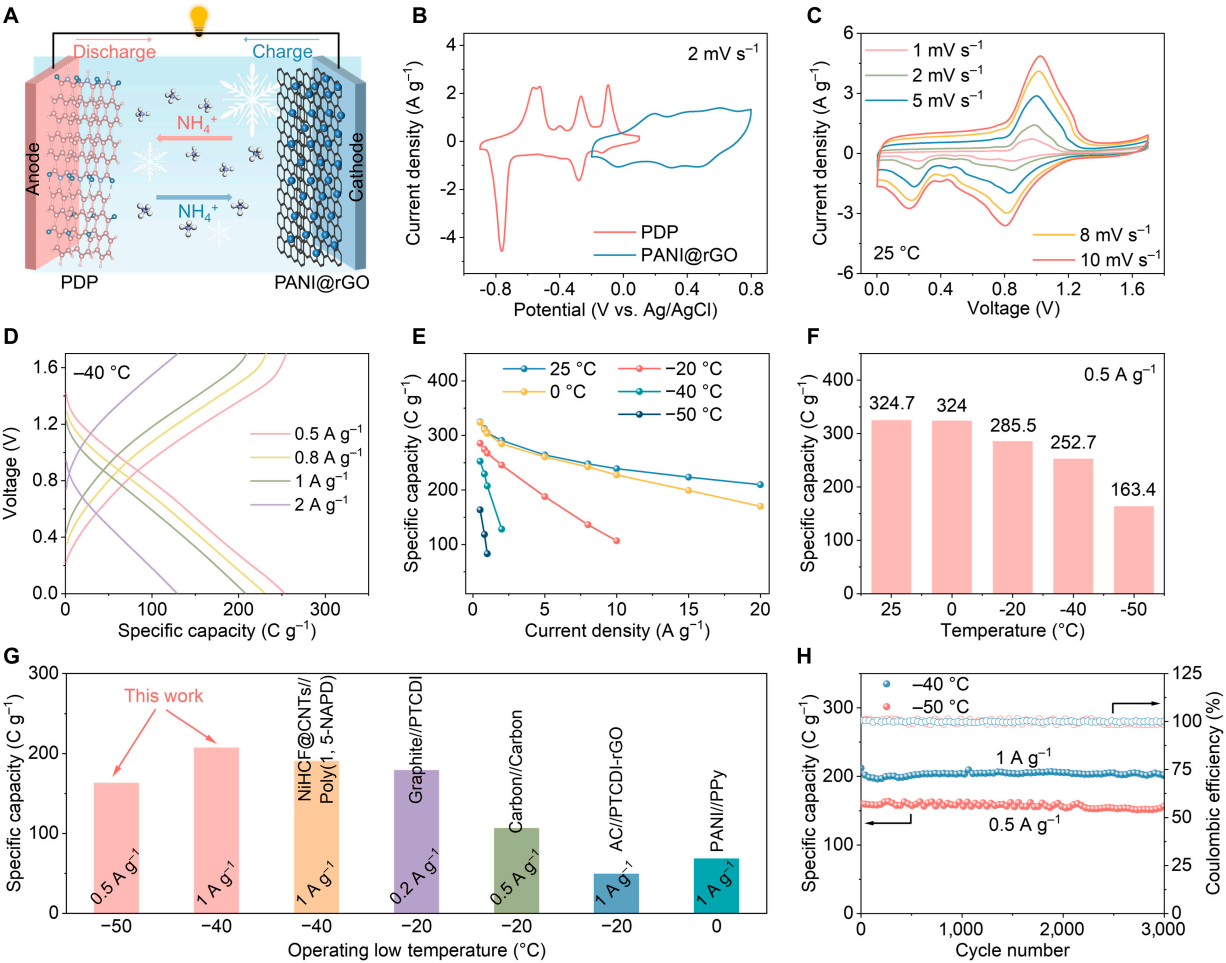
Electrochemical performance of PANI@rGO//PDP hybrid ammonium-ion capacitor at different temperatures. (A) Schematic structure of the PANI@rGO//PDP hybrid ammonium-ion capacitor. (B) CV curves of PDP and PANI@rGO electrodes at a scan rate of 2 mV s^−1^. (C) CV curves of the hybrid ammonium-ion capacitor at different scan rates under 25 °C. (D) GCD curves of the hybrid ammonium-ion capacitor at −40 °C. (E) Specific capacities at various current densities and temperatures. (F) Comparison of specific capacities for the hybrid ammonium-ion capacitor at 0.5 A g^−1^ under different temperatures. (G) Comparison of operating low temperature for ammonium-ion storage devices. (H) Cycling performance of the hybrid ammonium-ion capacitor at −40 and −50 °C.

A hybrid ammonium-ion capacitor was subsequently assembled using a PDP anode, PANI@rGO cathode, and EG60 electrolyte (Fig. 6A), and its electrochemical performance was systematically evaluated. The device achieves a working voltage of 1.7 V, primarily arising from the potential difference between the PDP anode (−0.9 to 0.1 V) and the PANI@rGO cathode (−0.2 to 0.8 V) (Fig. 6B). Figure 6C presents the CV curves of the PANI@rGO//PDP hybrid capacitor within the 0 to 1.7 V window at various scan rates. GCD curves recorded at 25 °C are shown in Fig. S48A, with specific capacity calculated based on the mass of PDP anode. The capacitor retains approximately 65% of its initial specific capacity when the current density is increased by 40 times (324.7 and 209.9 C g^−1^ at 0.5 and 20 A g^−1^, respectively). Furthermore, CV curves were measured at various temperatures (Fig. S47), with redox peak potentials corresponding to the charge/discharge platforms in the GCD curves (Fig. S48B to D). At −40 °C, the device provides a specific capacity of 252.7 C g^−1^ at 0.5 A g^−1^, which remains at 207.4 C g^−1^ when the current density is increased to 1 A g^−1^ (Fig. 6D). The capacity variation of the PDP electrode with current density and temperature is summarized in Fig. 6E. Notably, increased impedance at lower temperatures leads to reduced capacity (Fig. S49). Nevertheless, the PANI@rGO//PDP hybrid capacitor continues to operate at −50 °C, supplying discharge capacities of 163.4 C g^−1^ at 0.5 A g^−1^ (Fig. 6F). The operating temperature of this device surpasses the reported lower limits of other ammonium-ion storage systems, such as NiHCF@CNTs//Poly(1,5-NAPD) [55], Graphite//PTCDI [30], Carbon//Carbon [56], AC//PTCDI-rGO [57], and PANI//PPy [58] (Fig. 6G and Table S2). The hybrid capacitor also demonstrates outstanding cycling performance, achieving 82.2% capacity retention after 3,000 cycles at 25 °C (Fig. S50), 95.4% capacity retention after 3,000 cycles at −40 °C, and 97.5% capacity retention after 3,000 cycles at −50 °C (Fig. 6H). Moreover, compared with conventional LIBs, this device holds a distinct advantage in low-temperature performance (Table S3). These results point to considerable potential for all-organic ammonium-ion devices in low-temperature energy storage.

After 3,000 cycles, the PDP anode and PANI@rGO cathode in the hybrid ammonium-ion capacitor were characterized (Figs. S51 and S52). SEM images reveal that the PDP anode retains its well-defined nanorod morphology without apparent structural degradation, while the PANI@rGO cathode displays only slight surface roughening. XRD patterns indicate that the characteristic diffraction peaks of both electrodes remain unchanged after cycling, with no impurity phases observed. FTIR spectra further demonstrate no obvious changes in characteristic functional groups after cycling. These findings collectively affirm the excellent structural and chemical stability of the PDP and PANI@rGO electrodes, which underlies the high cycling stability of the device. The compositional stability of the EG60 electrolyte after long-term cycling at low temperature was assessed using ^1^H NMR, ^13^C NMR, and Raman spectroscopy (Fig. S53). The results indicate that after cycling, the positions and intensities of the O–H signal in the ^1^H NMR spectrum, the methylene carbon signal of EG in the ^13^C NMR spectrum, and the C–H stretching vibration peak in the Raman spectrum show no discernible changes, and no new characteristic peaks appear. This confirms that the main components of the electrolyte remain stable after 3,000 cycles, which is attributed to the synergistic effect between the electrode and the electrolyte.

Furthermore, low-/room-temperature cycling stability and temperature-shock tests were performed by alternating between 25 and −50 °C. As illustrated in Fig. S54A, the device delivers a stable capacity of 432 C g^−1^ after 50 cycles at 25 °C, which remains 270 C g^−1^ after holding at −50 °C for 2 h and continued cycling. Upon returning to 25 °C, the capacity fully recovers, with coulombic efficiency close to 100% throughout. Figure S54B shows that during temperature-shock cycling, the capacity slightly decreases during the initial cycles after switching to −50 °C and then stabilizes. After switching back to 25 °C, a similar recovery and stabilization behavior is observed, with no obvious capacity loss compared to the initial room-temperature performance. These results underscore the device’s excellent electrochemical robustness and structural stability under alternating and extreme temperature conditions.

## Conclusion

In summary, we present a carbonyl-rich conjugated polymer with fast NH_4_^+^ coordination kinetics for hybrid ammonium-ion capacitors under practical low temperatures. The charge storage behavior of the PDP electrode is determined by an intrinsic self-selectivity mechanism arising from the conjugated electron system, which enables a cooperative interaction between coordinated and synergistically coordinated carbonyl groups to rapidly transport NH_4_^+^ ions. The enhanced NH_4_^+^ storage capability endows PDP electrode with a high-rate performance (107 mAh g^−1^ at 20 A g^−1^) and stable low-temperature (−50 °C) operation. Integrative methods of experimental characterization and theoretical analysis are employed to uncover a reversible 4-step NH_4_^+^ coordination process involving a 4-electron transfer. Moreover, the constructed PANI@rGO//PDP all-organic hybrid ammonium-ion capacitors deliver an excellent capacity retention of 95.4% after 3,000 cycles at −40 °C, and can be operated for over 3,000 cycles at −50 °C. This work paves the way to develop ammonium-ion-based energy devices with organic polymers at low-temperature conditions.

## Materials and Methods

### Materials

PTCDA, DAAP, (NH_4_)_2_SO_4_, and EG were purchased from Shanghai Aladdin Biochemical Technology Co., Ltd. Aniline and ammonium persulfate [(NH_4_)_2_S_2_O_8_] were purchased from Shanghai Macklin Biochemical Co., Ltd.

### Synthesis of PDP

PDP was synthesized through a one-step solvothermal method. Initially, 4 mmol of DAAP and 4 mmol of PTCDA were added to 50 ml of *N*,*N*-dimethylformamide (DMF) and stirred thoroughly to ensure homogeneous mixing. The resulting mixture was then loaded into a 100-ml autoclave and kept at 180 °C for 10 h. After the reaction, the precipitate was collected and sequentially washed with DMF and ethanol to remove impurities, then dried under vacuum at 60 °C for 12 h to obtain the red powder.

### Synthesis of PANI@rGO

Firstly, graphene oxide (GO) was synthesized according to a modified Hummers’ method [59]. GO solution (2 mg ml^−1^) was then transferred into an autoclave and heated at 180 °C for 12 h. The obtained sample was repeatedly rinsed with deionized water, centrifuged, and then subjected to freeze-drying for 48 h to obtain rGO. For the synthesis of PANI@rGO composite, 0.365 ml of aniline and 30 mg of rGO were dispersed in 15 ml of 1 M HCl solution (designated as solution A). Separately, 0.228 g of (NH_4_)_2_S_2_O_8_ was dissolved in 5 ml of 1 M HCl to form solution B. Solution B was then gradually introduced into solution A under constant agitation. The polymerization was carried out in an ice bath to control the reaction rate and ensure uniform growth of the polymer chains. After 12 h of polymerization, a dark green product was obtained by vacuum filtration. The obtained material underwent lyophilization for 48 h to yield final PANI@rGO composite.

### Preparation of electrodes

The active materials (PDP or PANI@rGO), Ketjen Black, and polyvinylidene fluoride binder were mixed in a mass ratio of 7:2:1 in *N*-methylpyrrolidone (NMP) and subsequently coated onto carbon paper. The electrodes underwent vacuum drying at 60 °C for 24 h to thoroughly eliminate residual NMP. When assembling the hybrid ammonium-ion capacitor, the mass ratio of active materials between the cathode and anode was 1.2, and the specific capacity of hybrid ammonium-ion capacitor was calculated based on the mass of the anode active material. The theoretical specific capacity of PDP is determined using the following equation:

$$C=\frac{\mathrm{nF}}{3.6\;M}$$ (1)

where C refers to the specific capacity, n is the number of transferred electrons, F is the Faradic constant, and M represents the molecular weight of the active materials. Therefore, the theoretical specific capacity of PDP is calculated to be 178 mAh g^−1^.

### The self-supported carbon film preparation

Activated carbon, acetylene black, and polytetrafluoroethylene (PTFE, with a mass fraction of 10 wt%) were uniformly mixed in a mass ratio of 7:2:1 and thoroughly milled. Subsequently, the resulting mixture was punched into round self-supporting membranes of 12 mm diameter, followed by drying under vacuum at 60 °C for 24 h.

### Electrochemical measurements

Both 3-electrode and 2-electrode tests were conducted in Swagelok cells. CV, GCD, and EIS measurements were performed using a VMP3 electrochemical workstation (BioLogic, France). Cycling performance and GITT tests were carried out using the LAND system. Low-temperature environments (0, −20, −40, and −50 °C) were provided by refrigerators from the Jie Sheng brand.

The *b* value calculated process is shown as follows:

$$i=av^{b}$$ (2)

In this equation, i represents the peak current (mA) and ν represents the scan rate (mV s^−1^).

The ionic conductivity is calculated as follows:

$$\rho =\frac{L}{\mathrm{RS}}$$ (3)

In this equation, L represents the distance between 2 platinum electrodes (cm), R represents resistance of the electrolyte (Ω), and S represents the area of the platinum electrode (1 cm^−2^).

### Characterization methods

The XRD patterns of the composition and crystal structure for powder materials were obtained using a Rigaku Ultima IV diffractometer operating with Cu Kα radiation (λ = 1.5406 Å). The morphology of powder samples was characterized using a scanning electron microscope (JEOL JSM-7100F). The chemical valence states of the surface and near-surface regions of the electrode materials were analyzed using x-ray photoelectron spectroscopy (XPS, VG-MultiLab-2000). The structural characteristics were further analyzed using FTIR (Thermo Scientific Nicolet iS20) spectroscopy and Raman spectroscopy (Horiba LabRAM HR Evolution, λ = 532 nm). The heat resistance of experimental materials was assessed with a thermogravimetric analyzer (STA-449C). The freezing point was measured using differential scanning calorimeter (American TA Q2000). The presence of free radicals in the organic material was detected with an EPR spectrometer (Bruker E500). The molecular structure of the material was determined using an NMR spectrometer (AVANCE NEO 400 MHz). Molecular ordering in the bulk powder state was assessed using powder WAXS measurements (Xenocs-Xeuss 2.0). The wettability of material surface was evaluated using a contact angle goniometer (JC2000D7M).

### Theoretical calculation detail

Spin-polarized DFT calculations [60,61] were performed with the Vienna ab initio simulation package (VASP), using plane-wave basis sets and the projector augmented-wave (PAW) method [62,63]. The exchange-correlation interactions were modeled within the generalized gradient approximation using the Perdew–Burke–Ernzerhof functional [64]. The energy cutoff was set to 450 eV. The Brillouin-zone integrations were carried out with a Γ-centered Monkhorst-Pack k-point mesh of 1 × 1 × 1. Structural relaxations were conducted until the residual atomic forces were below 0.03 eV/Å and the total energy convergence threshold was 10^−5^ eV. The Gibbs free energy is expressed as $\Delta G_{f}\left(n\right)=\Delta E_{\text{total}}\left(n\right)+\Delta G_{T}\left(n\right)$, where $\Delta G_{T}\left(n\right)=\Delta E_{\mathrm{zpe}}\left(n\right)-T\Delta S\left(n\right)$, n represents the corresponding reaction step, while $\Delta E_{\text{total}}\left(n\right)$, $\Delta E_{\mathrm{zpe}}\left(n\right)$, and $\Delta S\left(n\right)$ represent the total energy difference, zero-point correction energy, and entropy change at 298 K for each intermediate step n, respectively. The visualizations of the crystal structures and charge density distributions were generated using the VESTA code.

### MD simulations

MD simulations were performed using the Forcite module in Materials Studio with the COMPASS III force field to investigate the solvation structures and particle transport behavior in the electrolyte. Each MD simulation cell contained approximately 2,500 atoms. A time step of 1 fs was employed, and all supramolecular systems were simulated in the canonical (NVT) ensemble at constant particle number, volume, and temperature for 300,000 steps, corresponding to a total simulation time of 3 ns. The electrolyte density was adjusted to match the corresponding experimental values.

## Data Availability

The data that support the findings of this study are available from the corresponding author upon reasonable request.
