# Supplementary material for: Conjugation-Induced Self-Selective Coordination Enables Organic Polymers with Low-Temperature Ammonium-Ion Storage
Source: Research (Wash D C). 2026 Mar 9;9:1160. doi: 10.34133/research.1160 (PMC12968395; doi:10.34133/research.1160)
Supplement: Supplementary 1 — Figs. S1 to S54 Tables S1 to S3 [file research.1160.f1.docx]

Supporting Information

**Conjugation-Induced Self-Selective Coordination Enables Organic Polymers with Low-Temperature Ammonium-Ion Storage**

*Xinji Zhou^1^, Tiezhu Xu^1^, Miaoran Zhang^1^, Tengyu Yao^1^, Zhenming Xu^1^, Duo Chen^1^, and Laifa Shen^1^**

^1^Jiangsu Key Laboratory of Materials and Technologies for Energy Storage, College of Materials Science and Technology, Nanjing University of Aeronautics and Astronautics, Nanjing, Jiangsu, 210016, China

^*^Address correspondence to: lfshen@nuaa.edu.cn


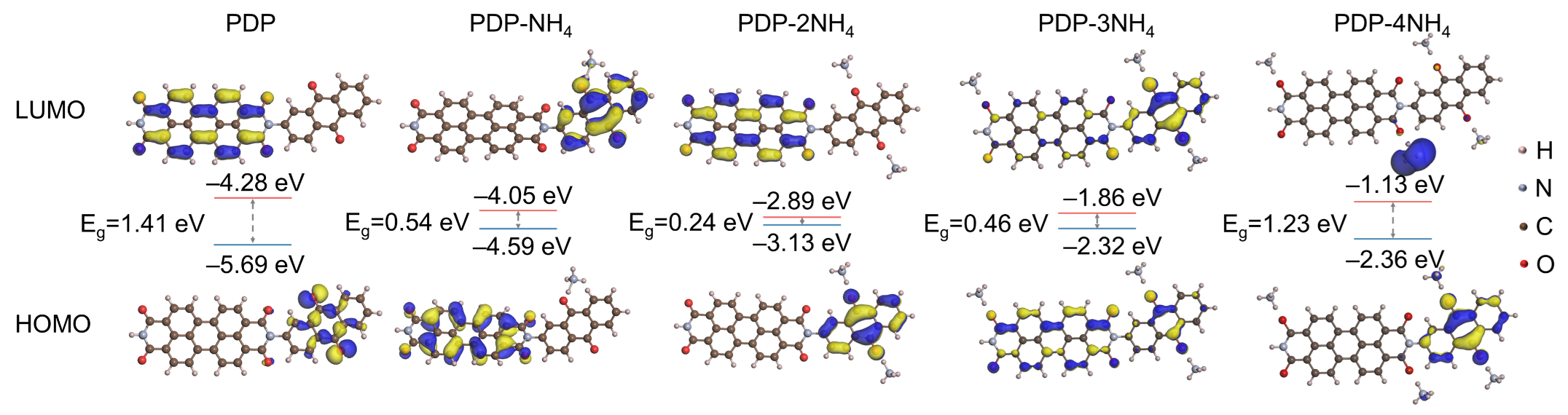


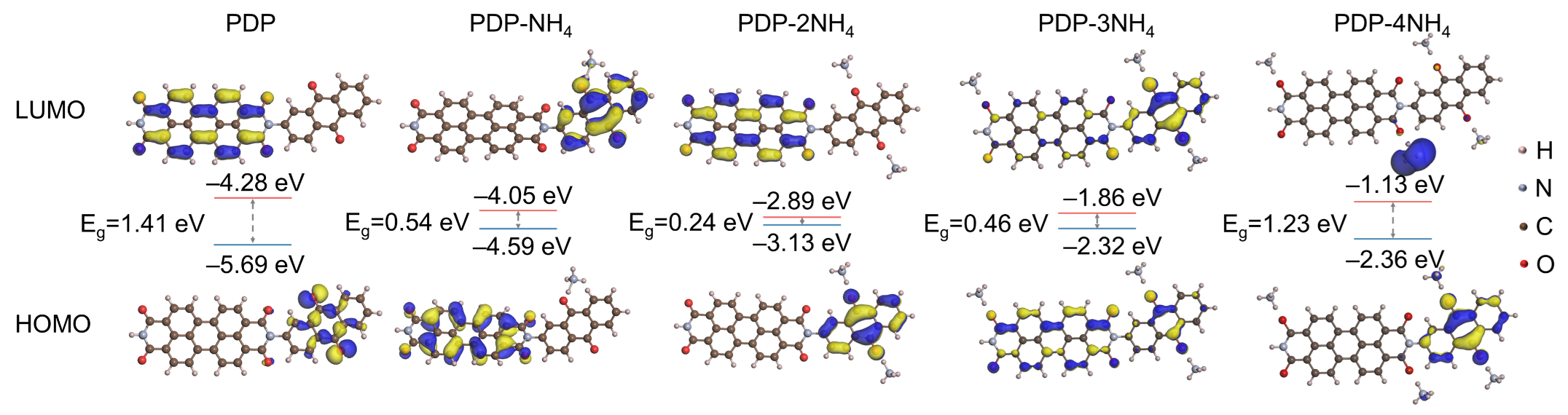


**Figure S1.** Relative HOMO/LUMO energy levels and energy gaps of PDP and PDP-xNH_4_ based on the DFT calculation.


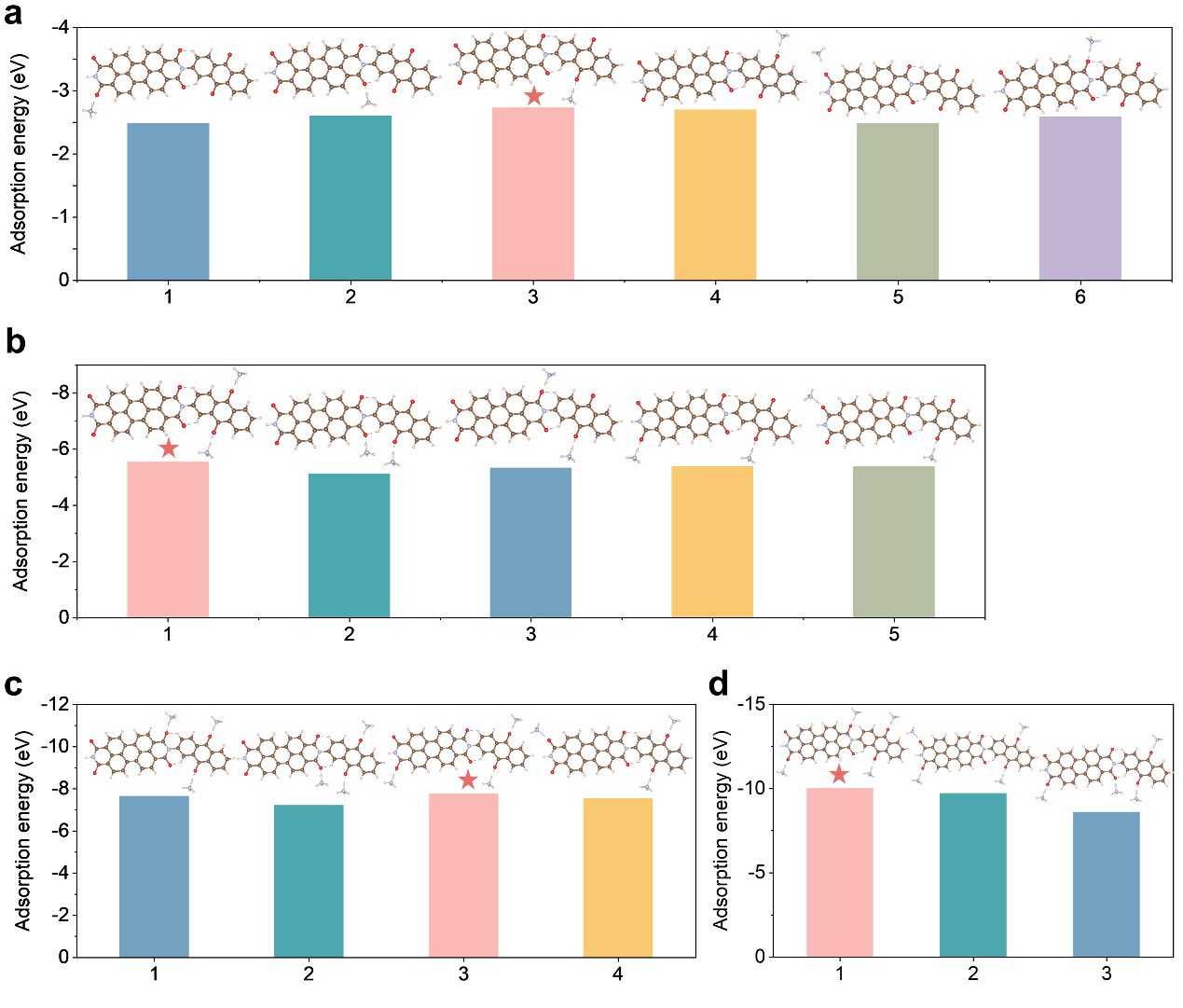


**Figure S2.** Adsorption energies of PDP with (a) one, (b) two, (c) three, and (d) four NH_4_^+^ ions coordinated at different sites.


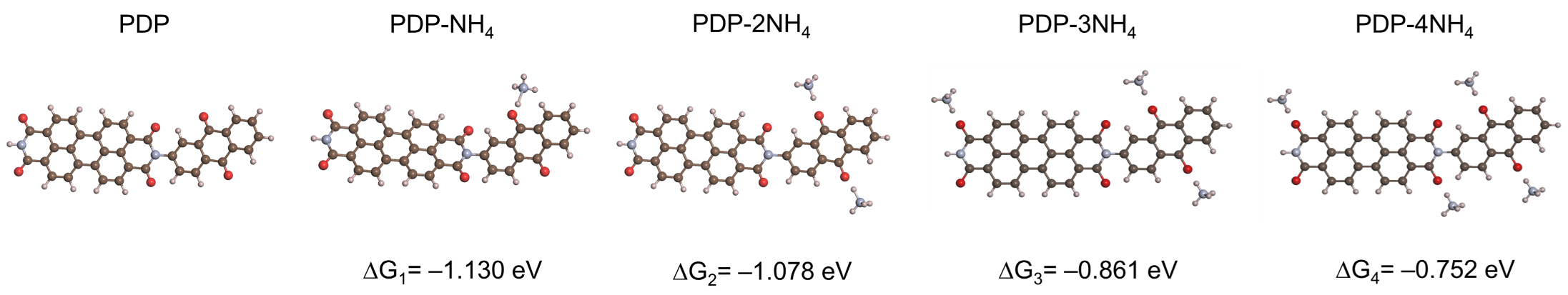


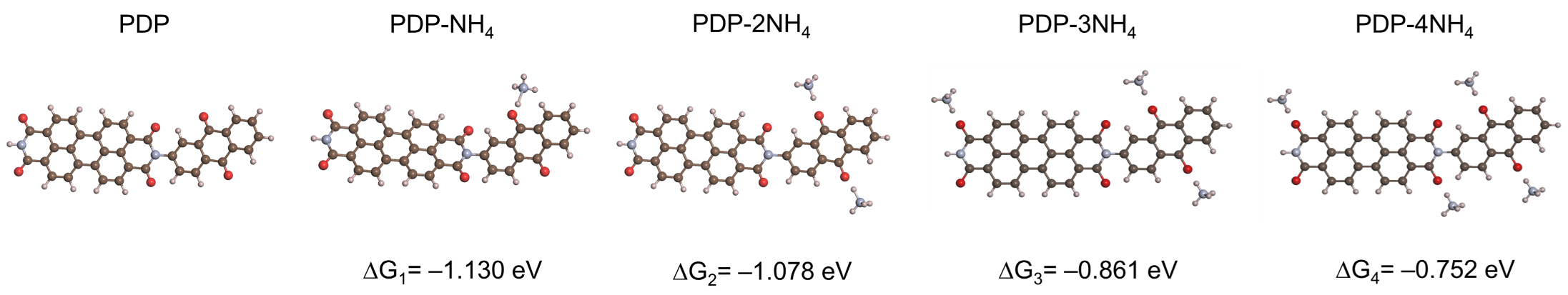


**Figure S3.** Calculated ∆G of PDP-xNH_4_.


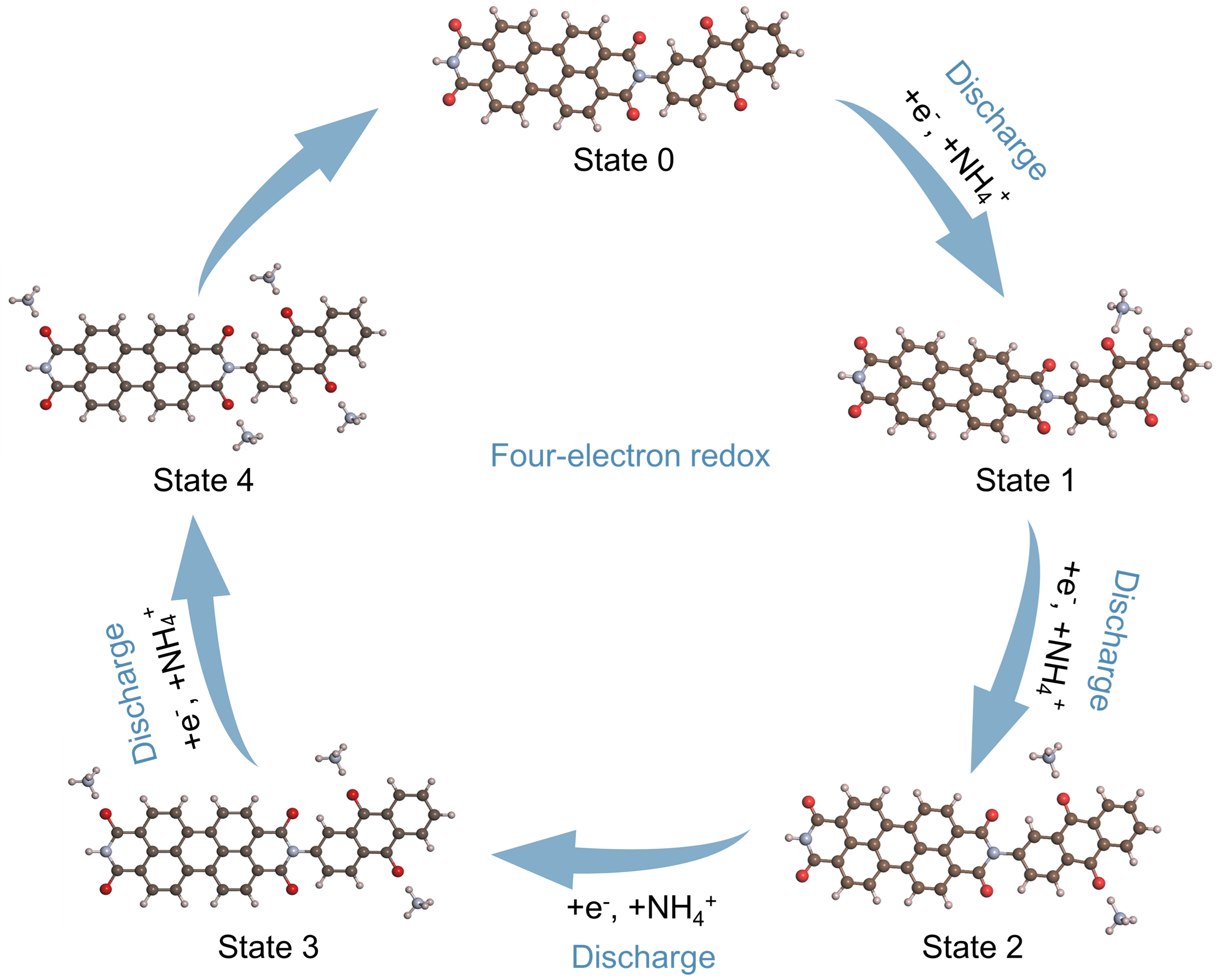


**Figure S4.** NH_4_^+^ storage pathway obtained from simulations of PDP electrode in the redox process.


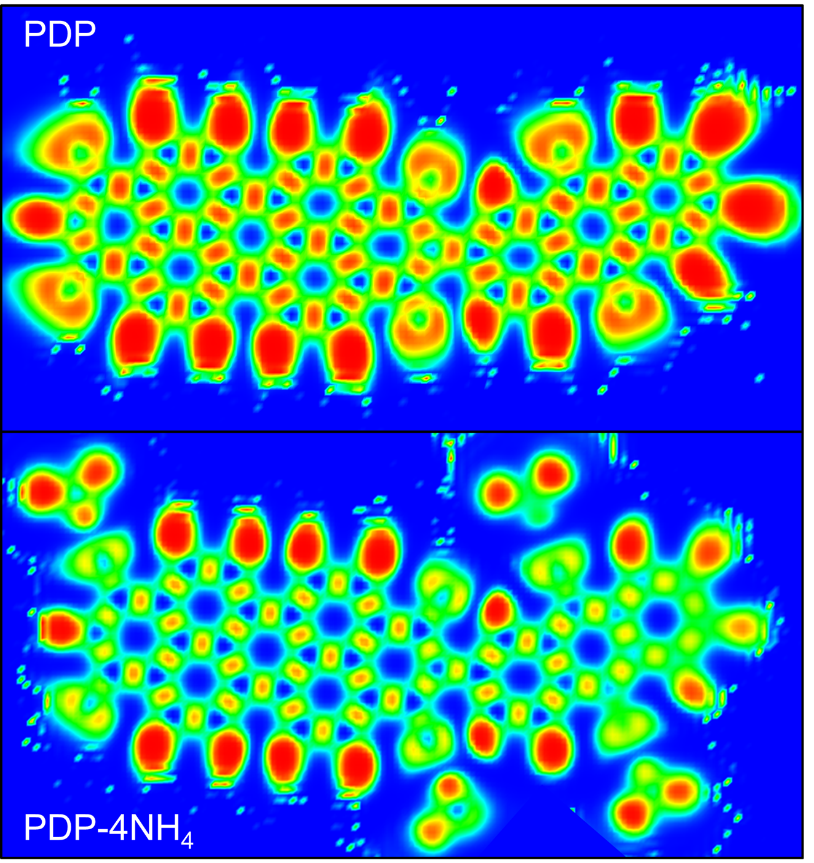


**Figure S5.** ELF map of PDP and PDP-4NH_4_.


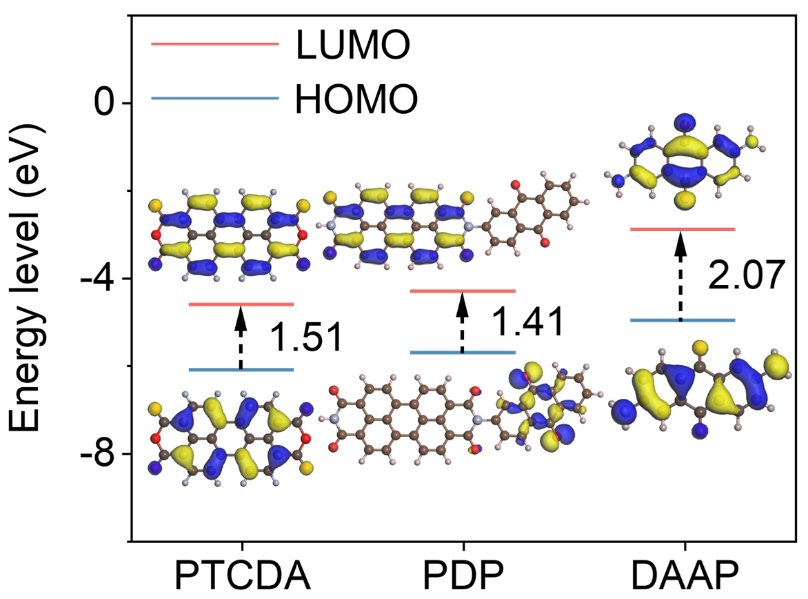


**Figure S6.** Relative HOMO/LUMO energy levels and energy gaps of PDP, PTCDA, and DAAP.

**
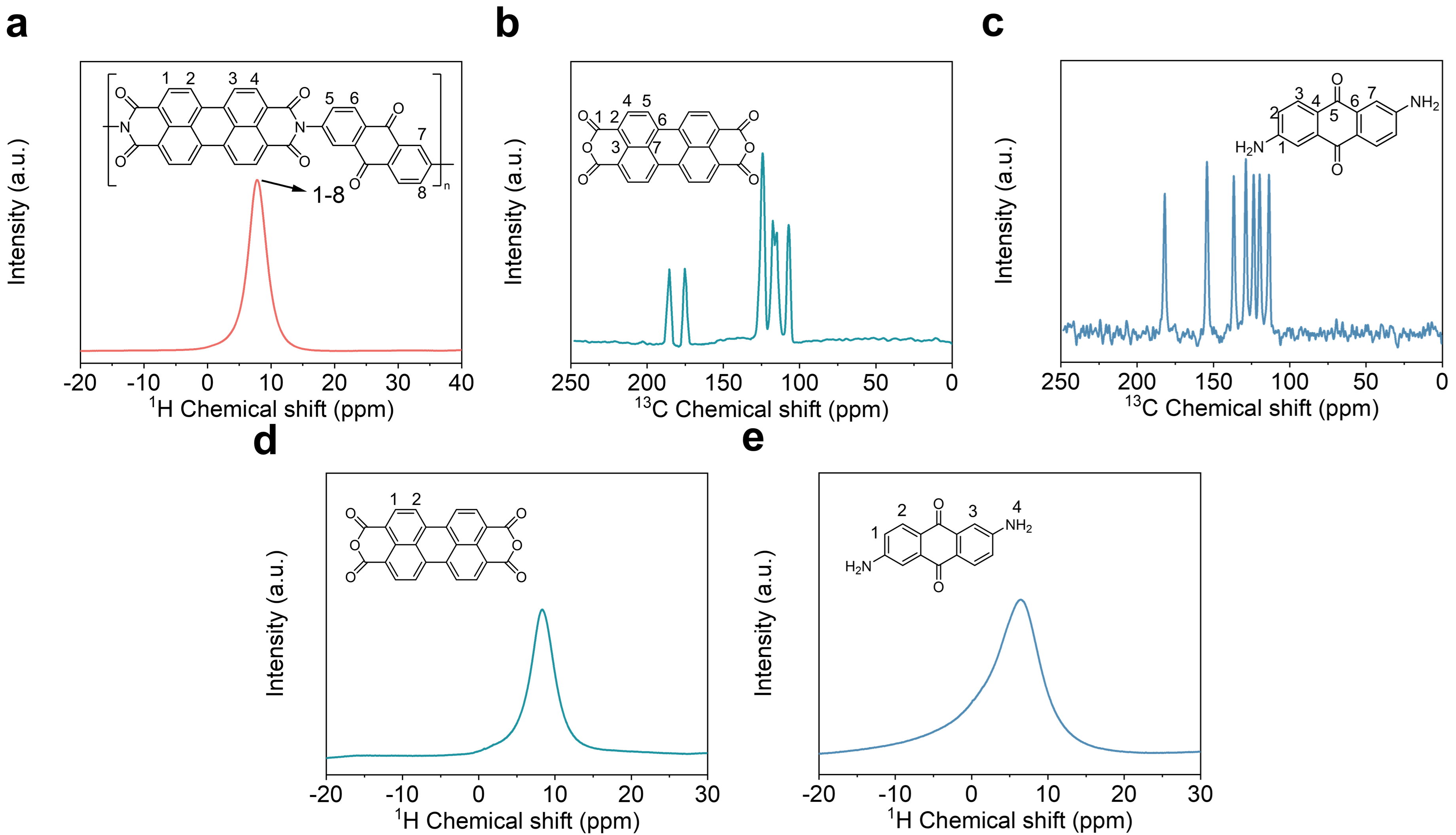
**

**Figure S7.** ^1^H NMR spectra of (a) PDP, (d) PTCDA, and (e) DAAP. ^13^C NMR of (b) PTCDA and (c) DAAP.

**
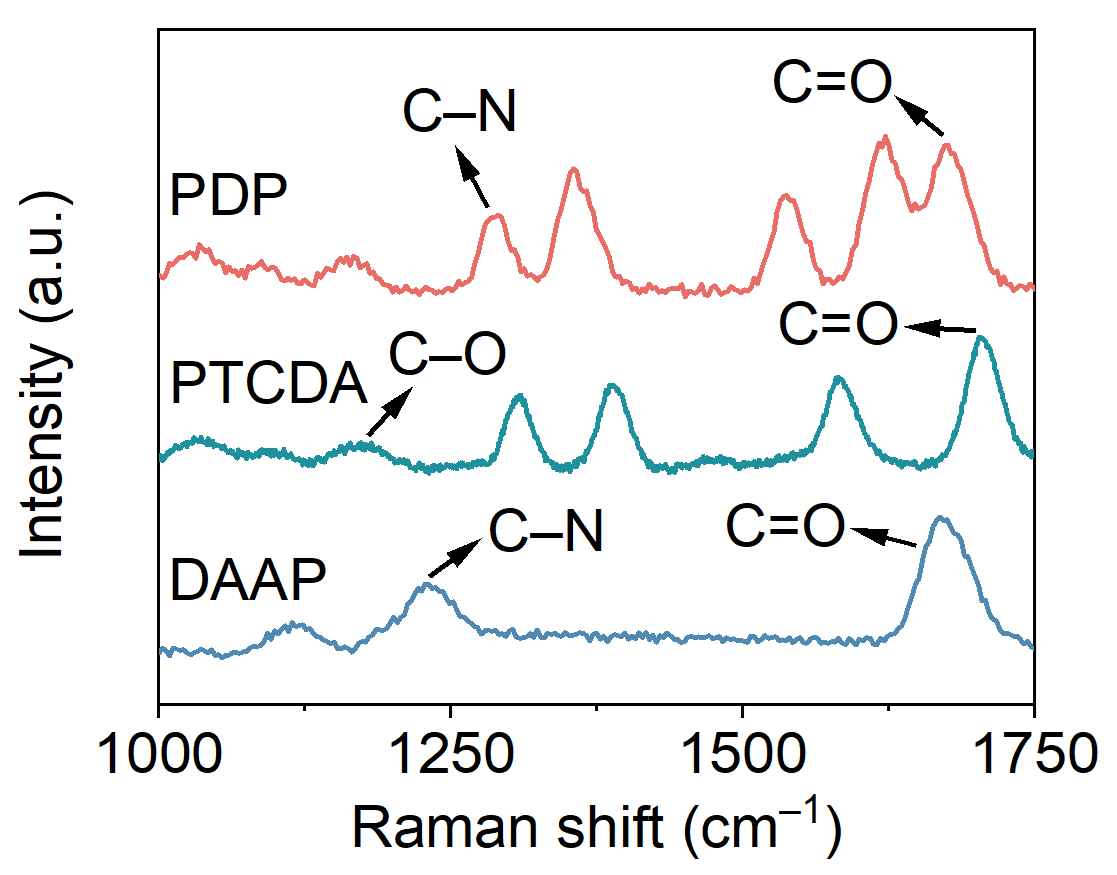
**

**Figure S8.** Raman spectra of PDP polymer and two monomers.

**
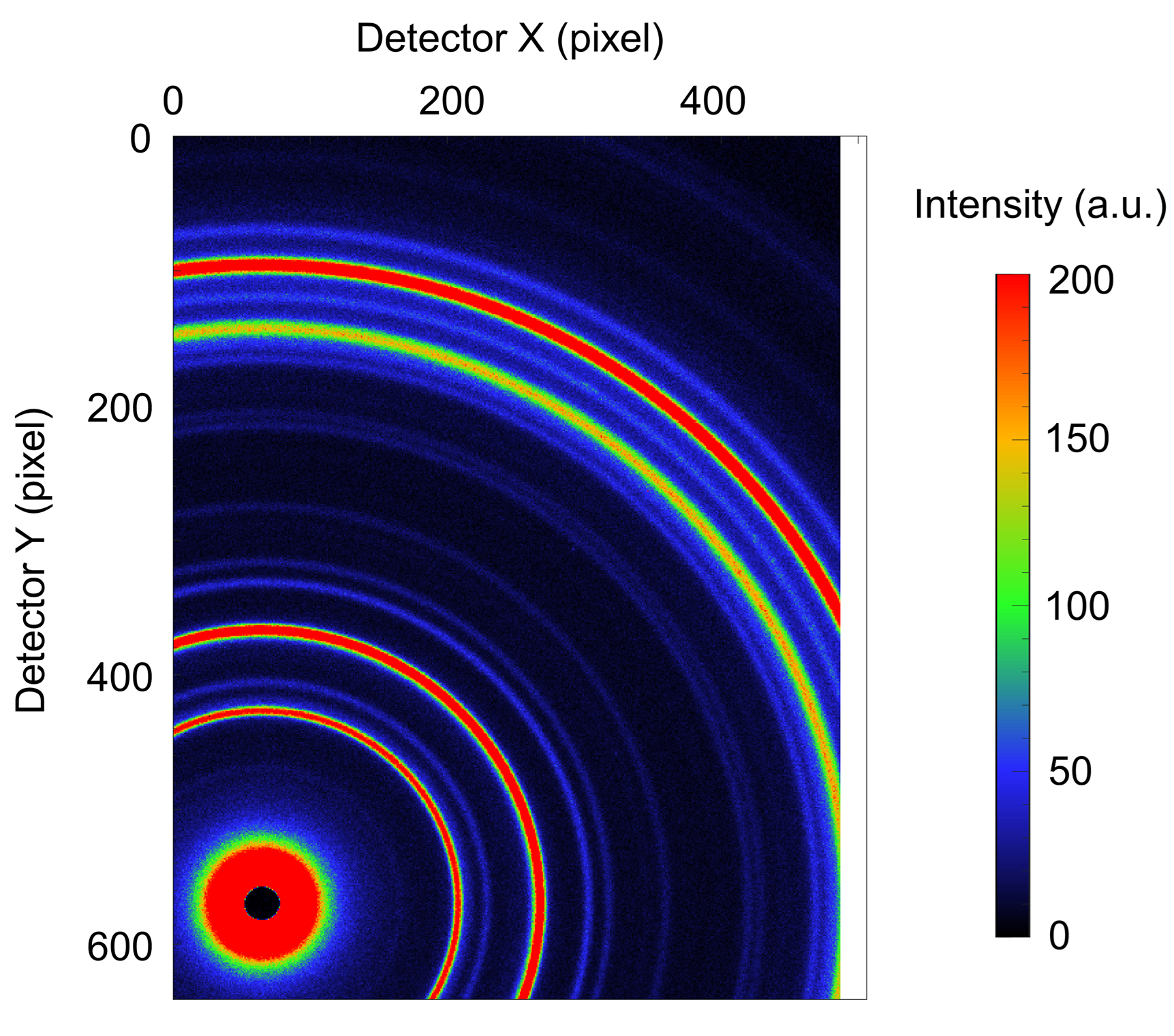
**

**Figure S9.** 2D WAXS pattern of PDP collected in transmission geometry. Axes correspond to detector coordinates (pixels), and the color scale represents scattering intensity (a.u.).

**
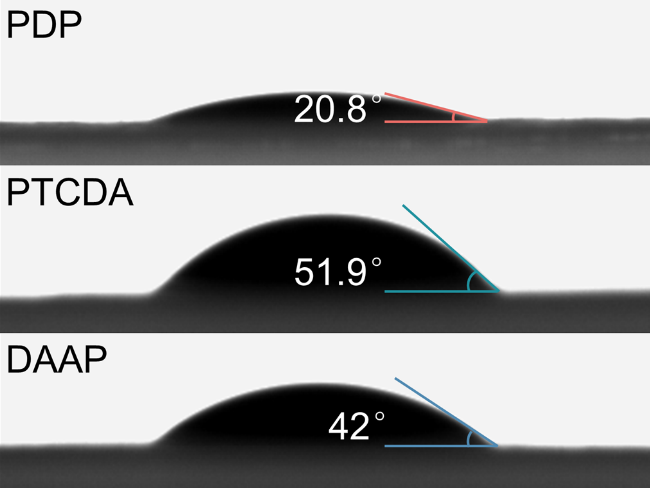
**

**Figure S10.** Images of the contact angle for PTCDA, DAAP, and PDP.

**
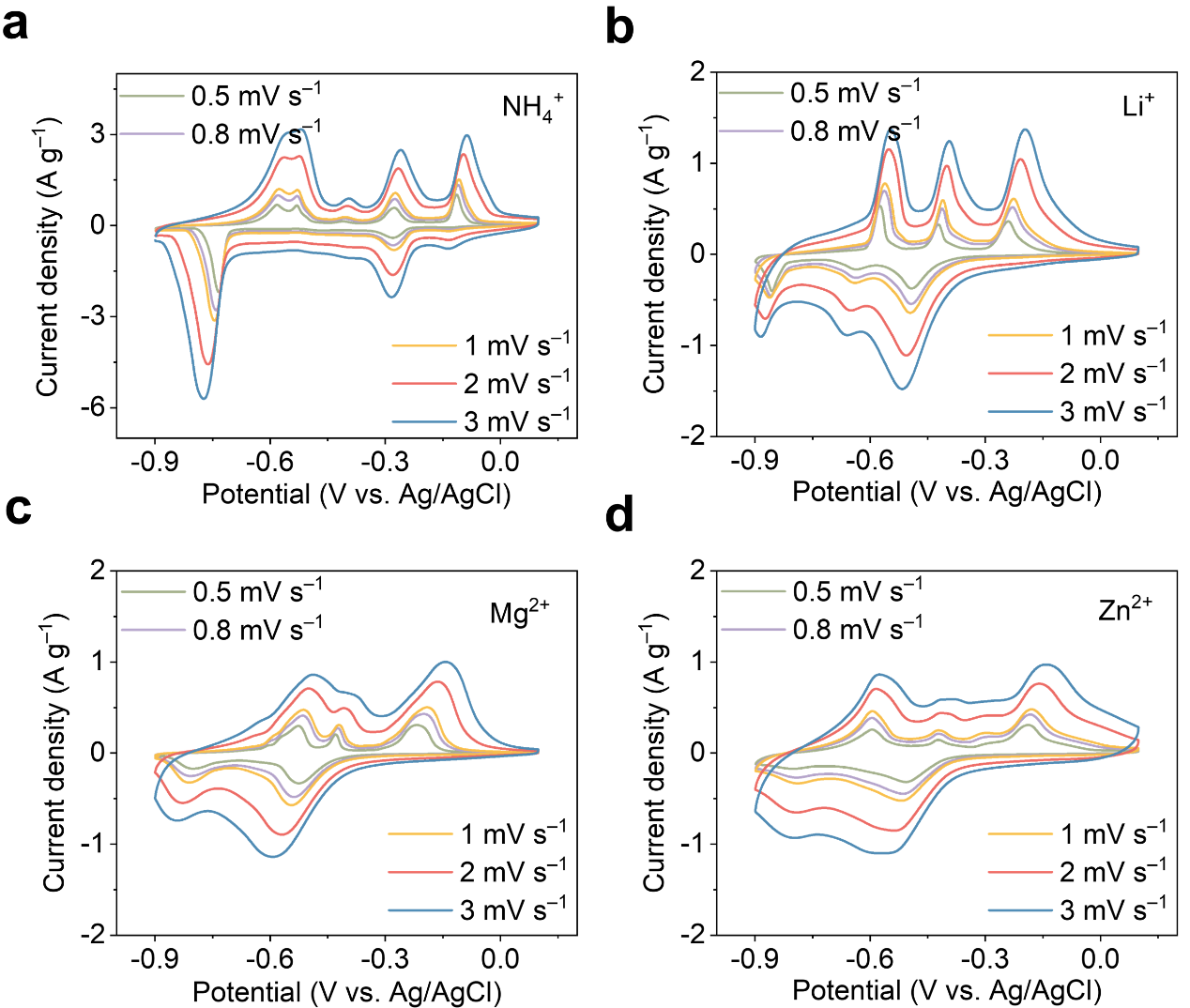
**

**Figure S11.** CV curves in different cation-based electrolytes at various scan rates.

**
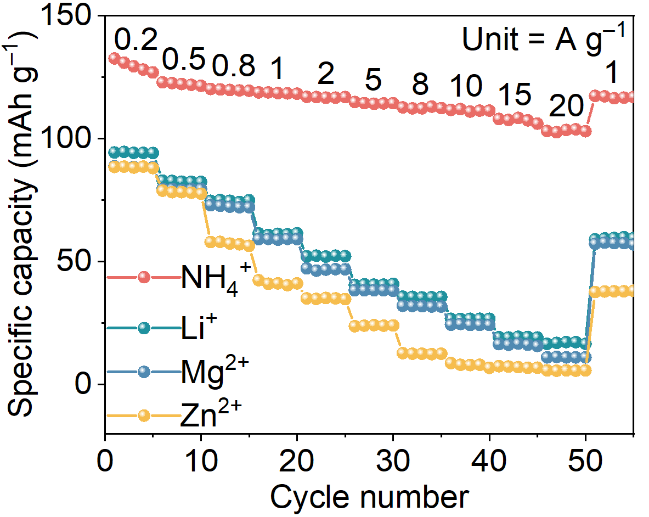
**

**Figure S12.** Rate performance of PDP electrode in different cation-based electrolytes.


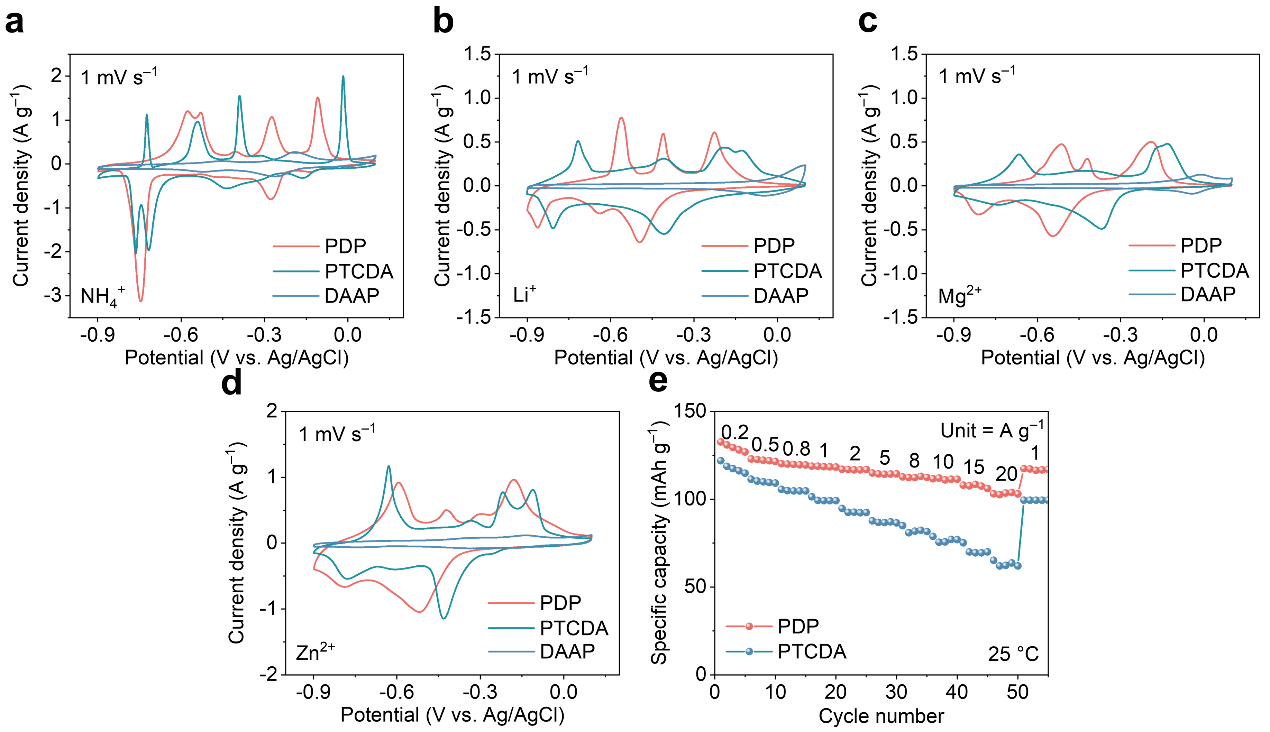


**Figure S13.** CV curves of PDP, PTCDA, and DAAP in (a) NH_4_^+^, (b) Li^+^, (c) Mg^2+^, and (d) Zn^2+^ electrolytes at a scan rate of 1 mV s^–1^. (e) Comparison of rate performance of PDP and PTCDA at 25 °C.


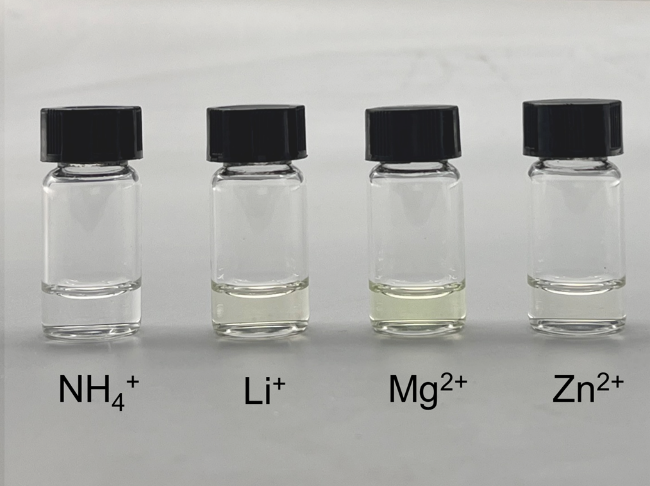


**Figure S14.** Photographs of PDP electrode after cycling in the four different electrolytes.


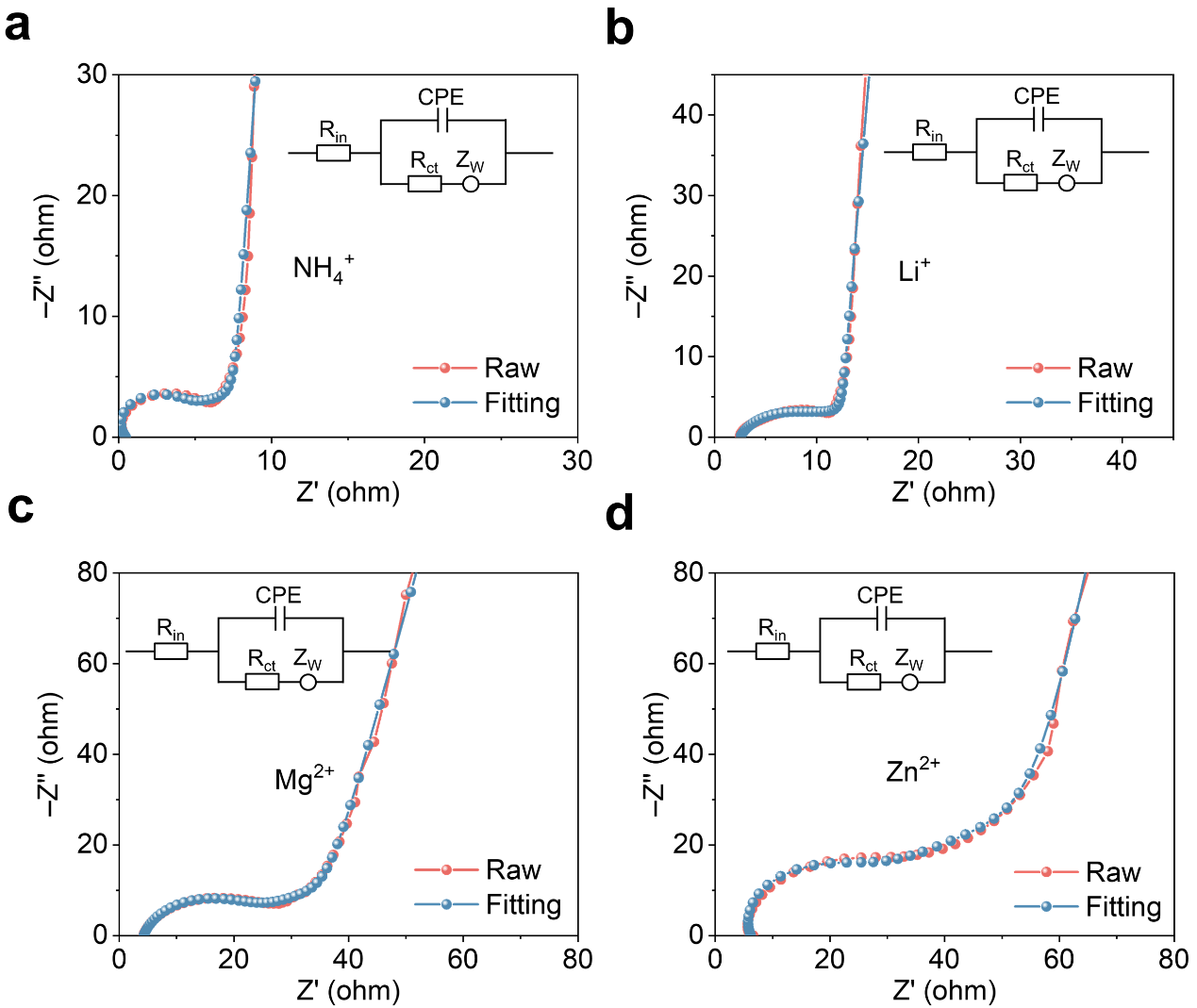


**Figure S15.** EIS plots of PDP electrode in four different electrolytes before electrochemical activation. (The tests are conducted at the open circuit potential after immersing the electrodes in the electrolytes for 20 mins.)


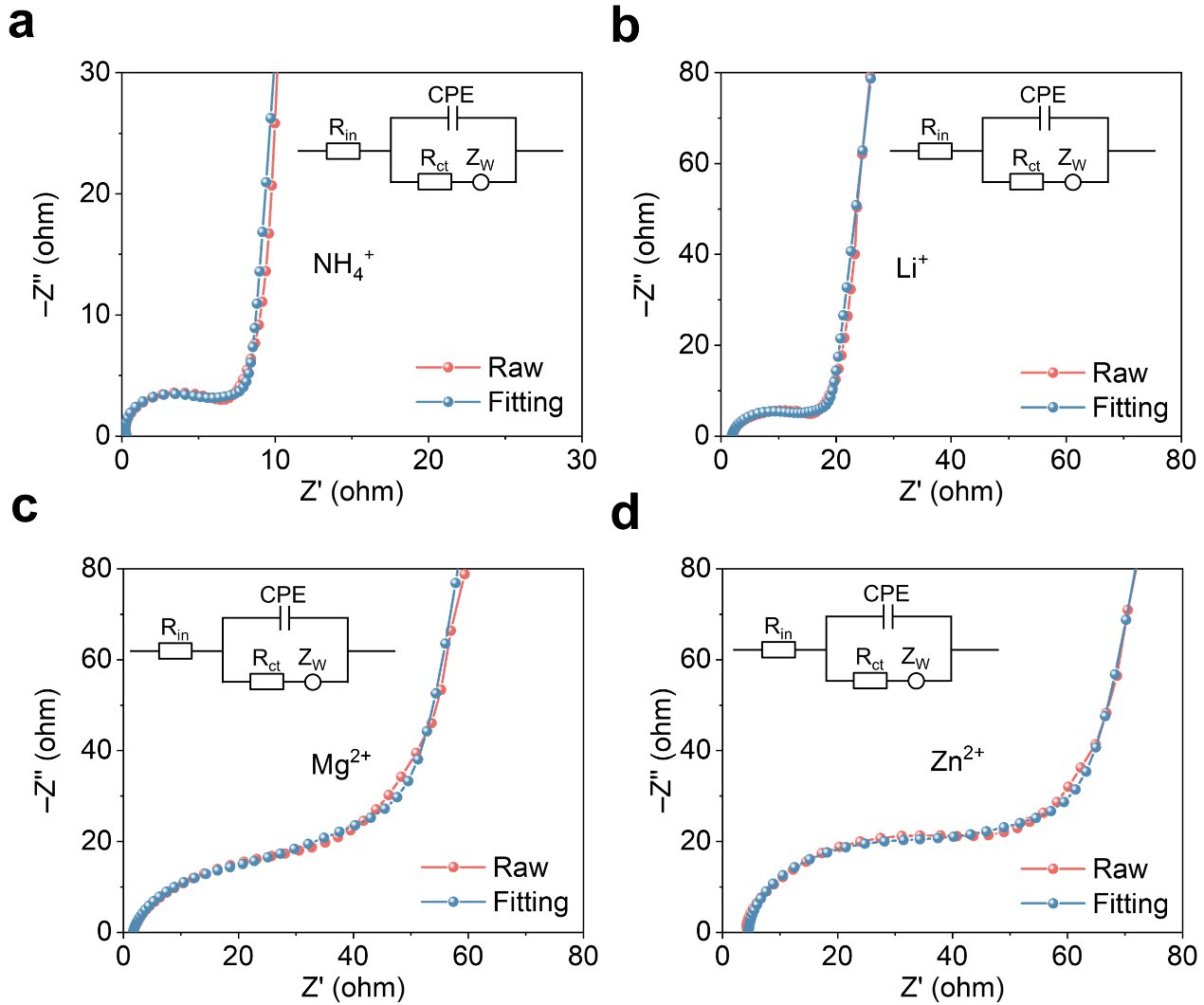


**Figure S16.** EIS plots of PDP electrode in four different electrolytes after electrochemical activation. (The tests are conducted after 20 cycles CV at a scan rate of 1 mV s^–1^.)


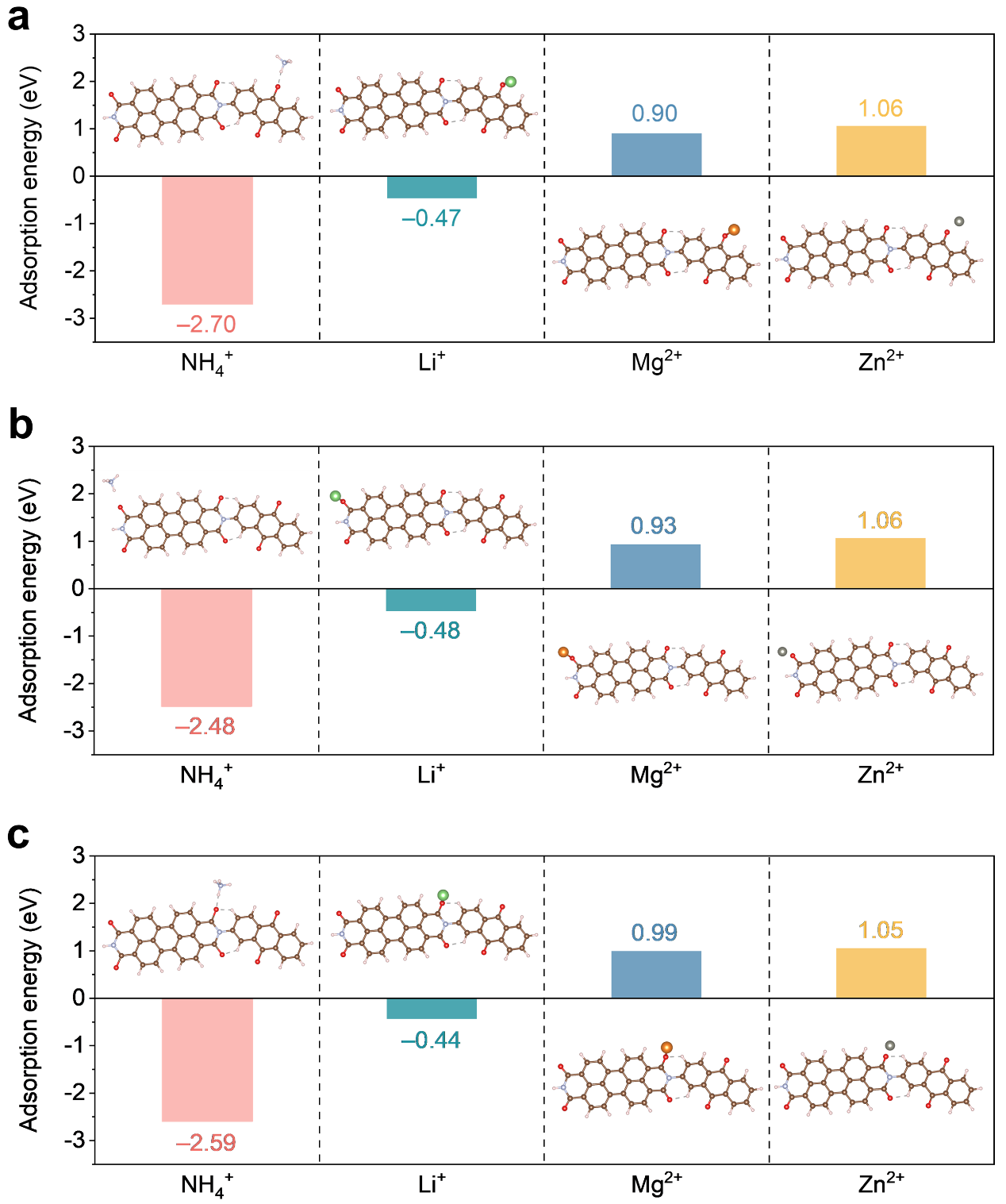


**Figure S17.** Comparison of cation adsorption energies within single-coordination structures at different sites.


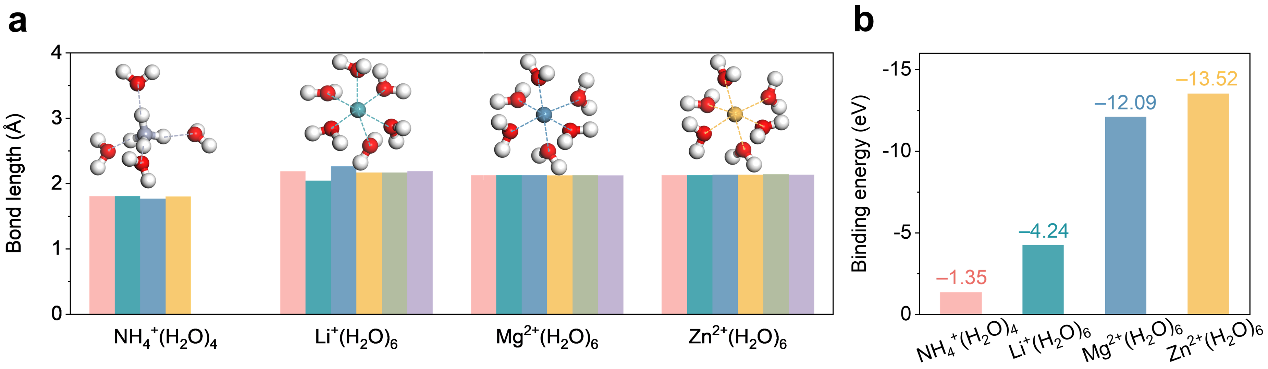


**Figure S18.** (a) Bond lengths in the solvation structures formed by the coordination of different cations with H_2_O molecules. (b) Binding energies between different cations and H_2_O molecules.


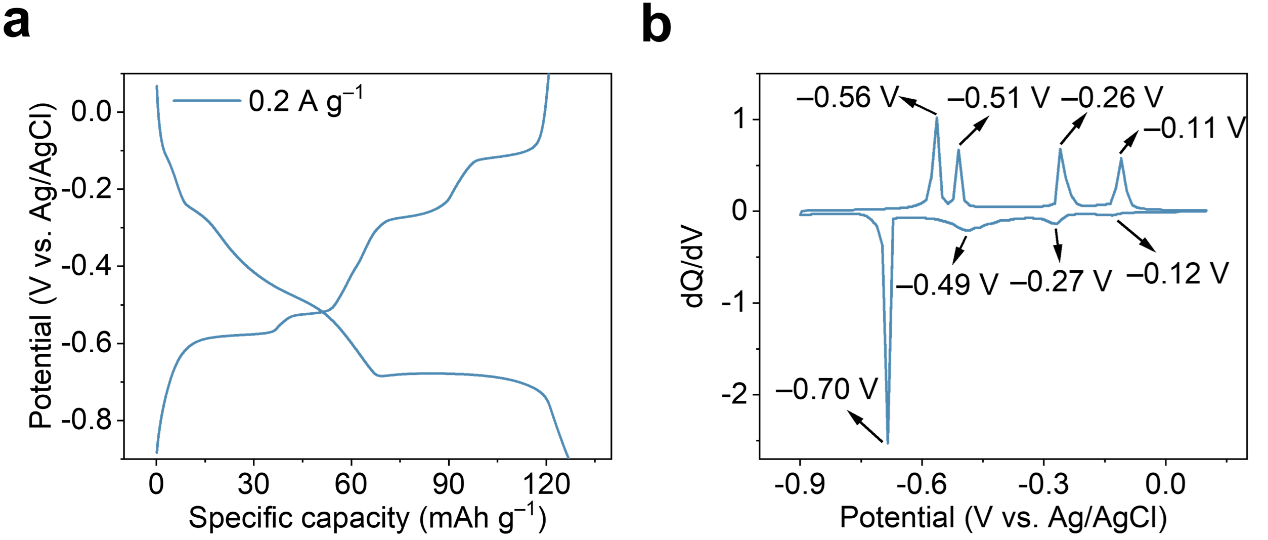


**Figure S19.** (a) GCD curve of PDP electrode at 0.2 A g^–1^. (b) dQ/dV curve obtained from the previous GCD curve.

**
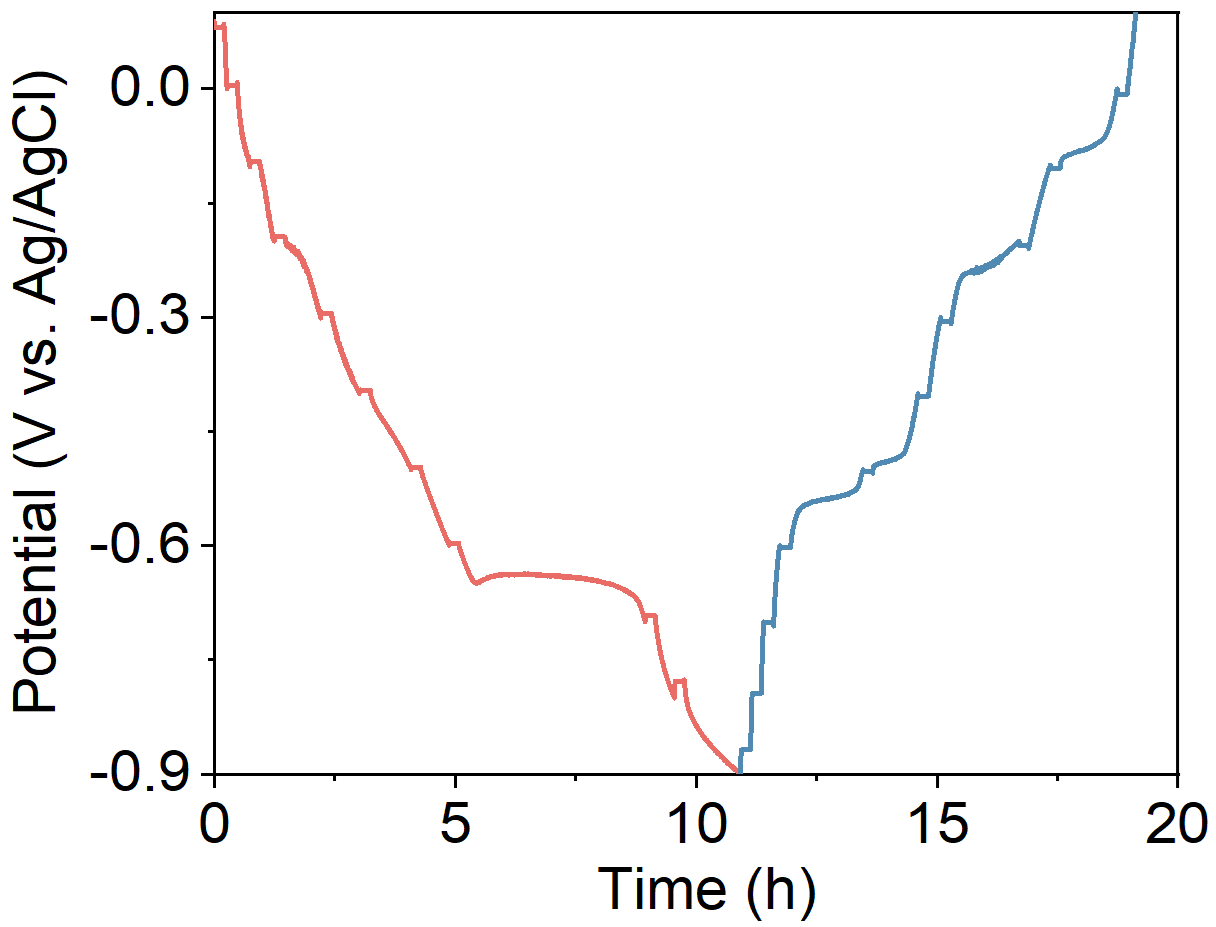
**

**Figure S20.** Charge/discharge curves of PDP electrode during in-situ EIS measurement.


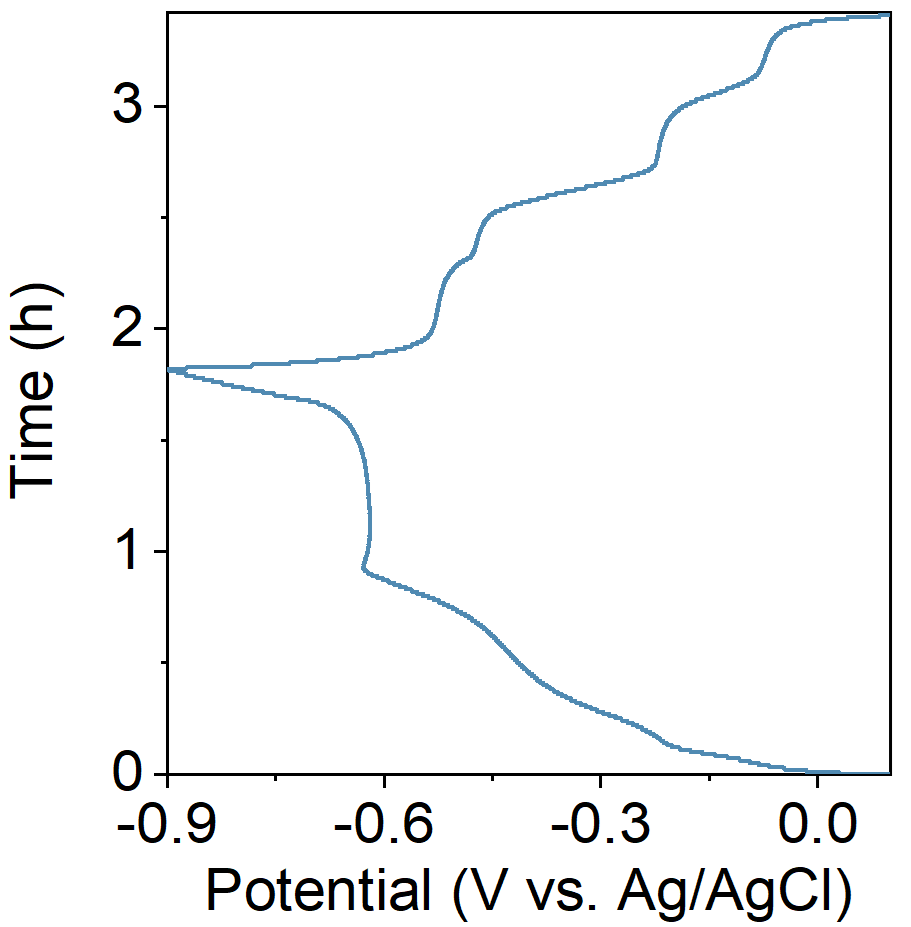


**Figure S21.** Charge/discharge curves of PDP electrode during in-situ FTIR characterization.


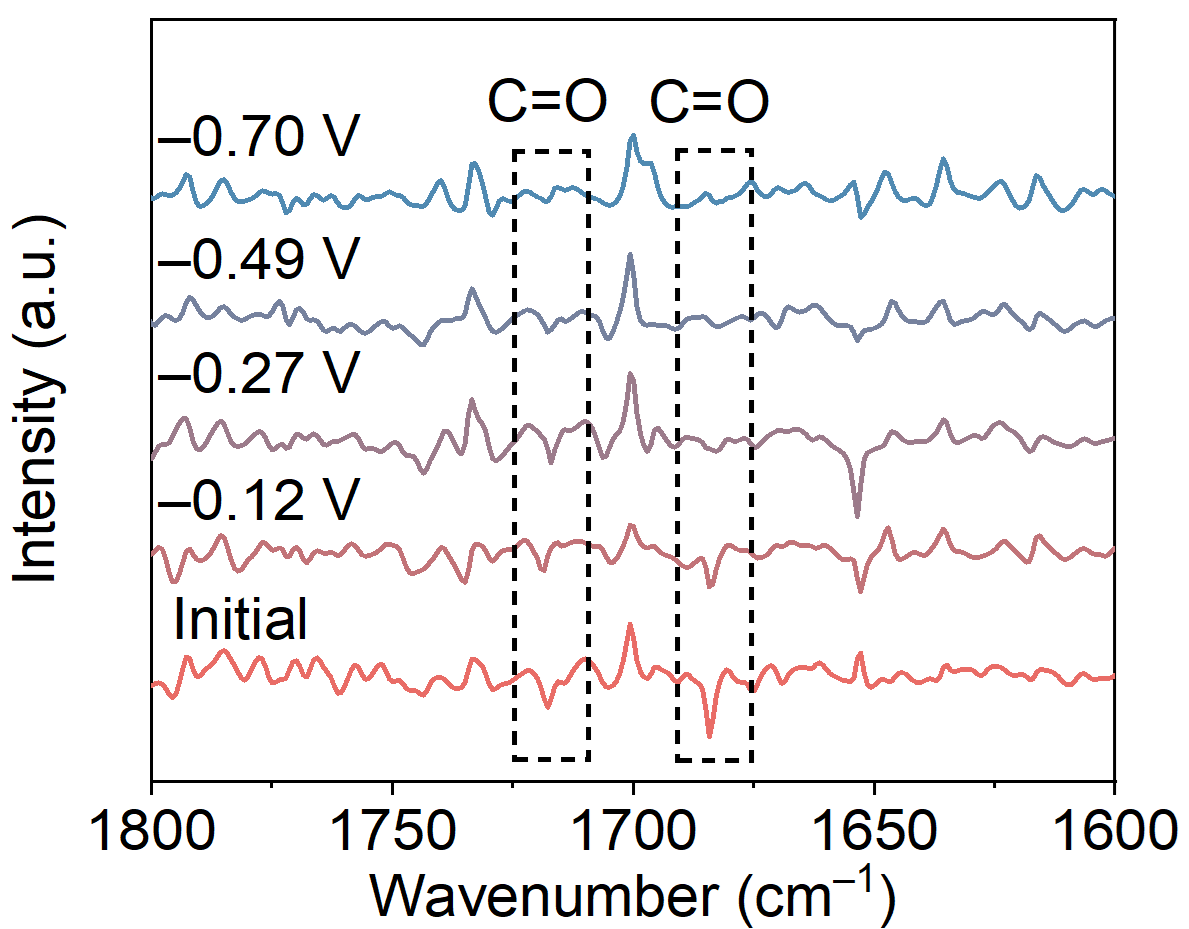


**Figure S22.** The FTIR spectra at the initial potential and at the potentials of the reduction peaks.


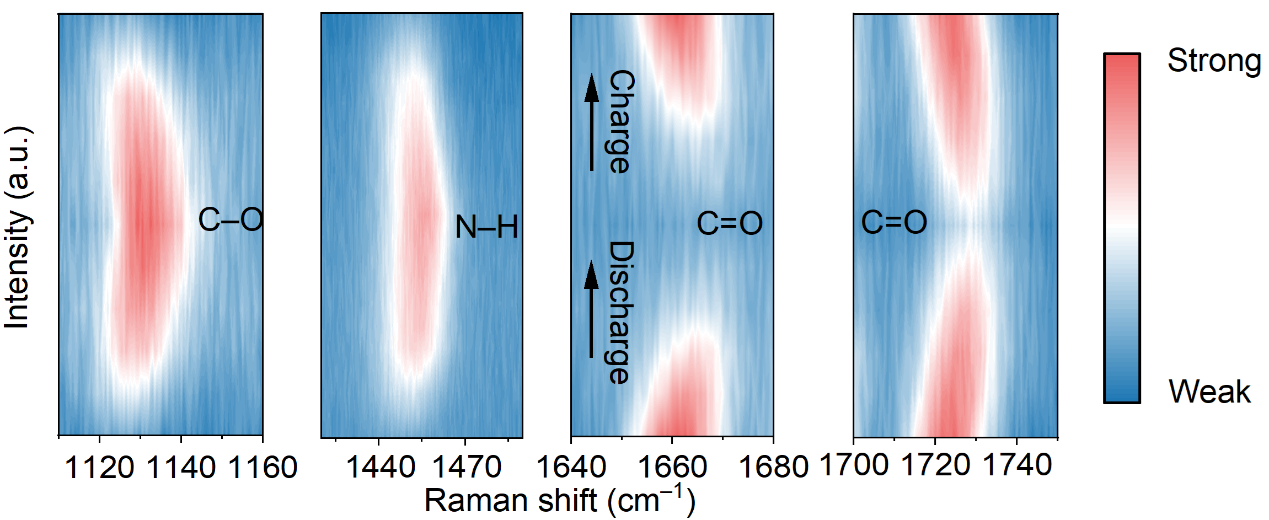


**Figure S23.** In-situ Raman spectra of PDP electrode at room temperature.


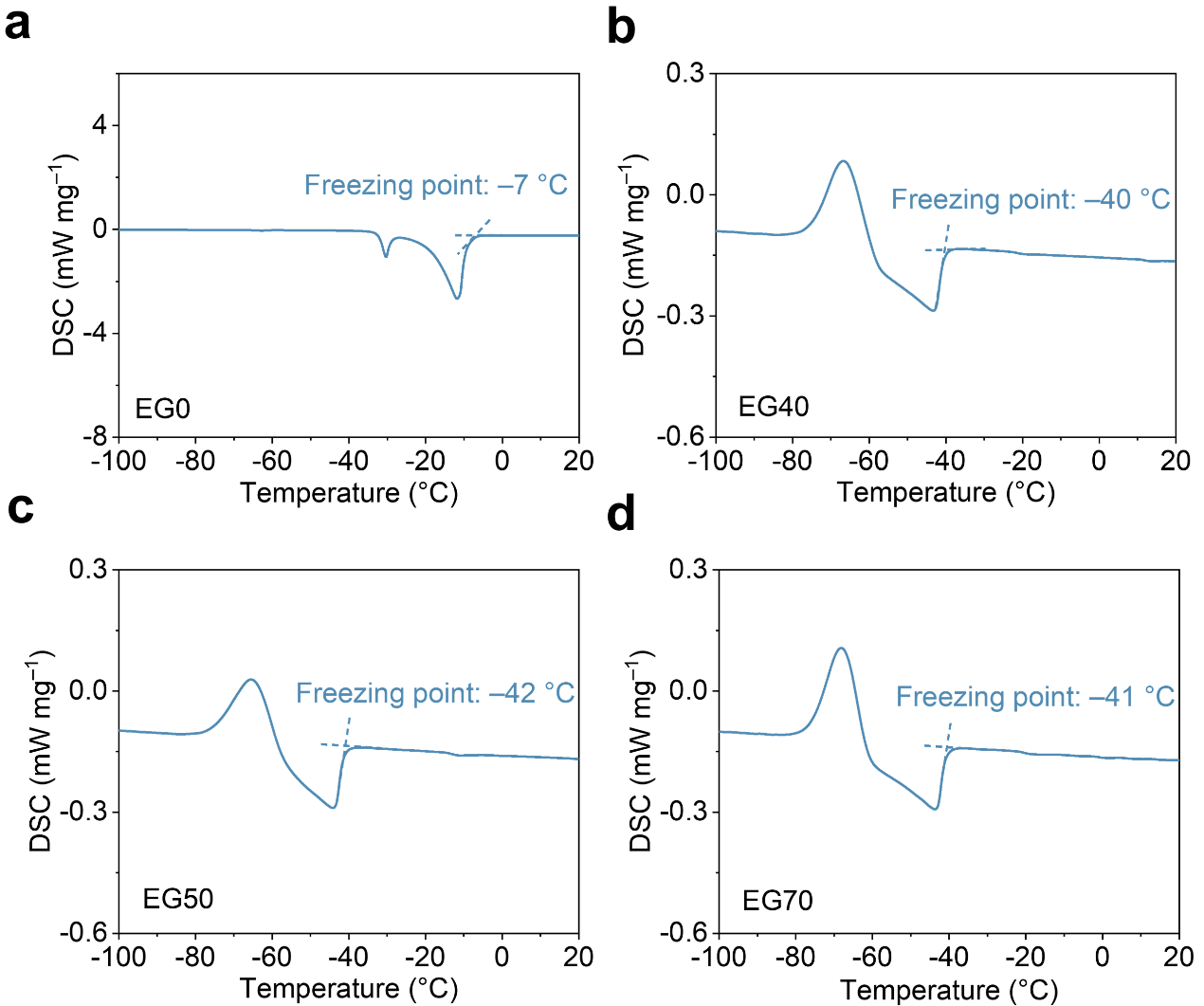


**Figure S24.** DSC curves of (a) EG0, (b) EG40, (c) EG50, and (d) EG70.


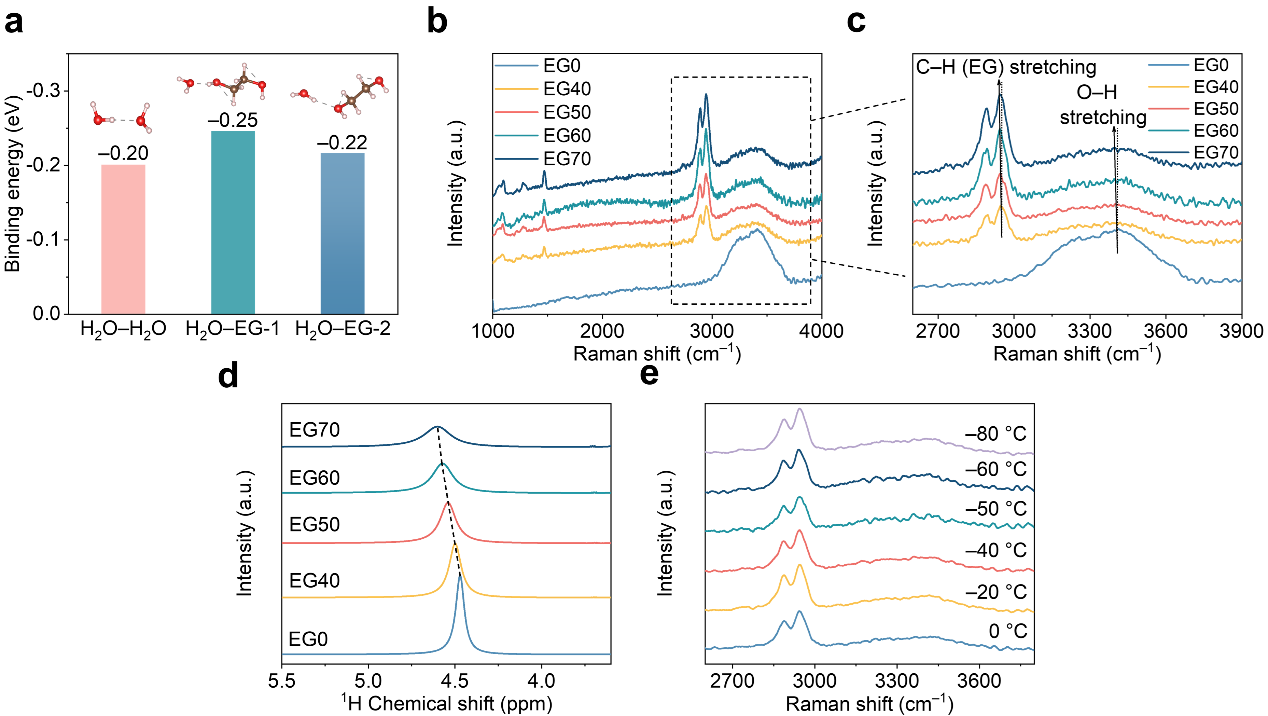


**Figure S25.** (a) Calculated binding energy of hydrogen bonds. (b) Raman spectra of electrolytes with different composition ratios at 25 °C. (c) Magnified view of the selected region in Figure (b). (d) ^1^H NMR spectra of electrolytes with different composition ratios at 25 °C. (e) Raman spectra of EG60 at various low temperatures.


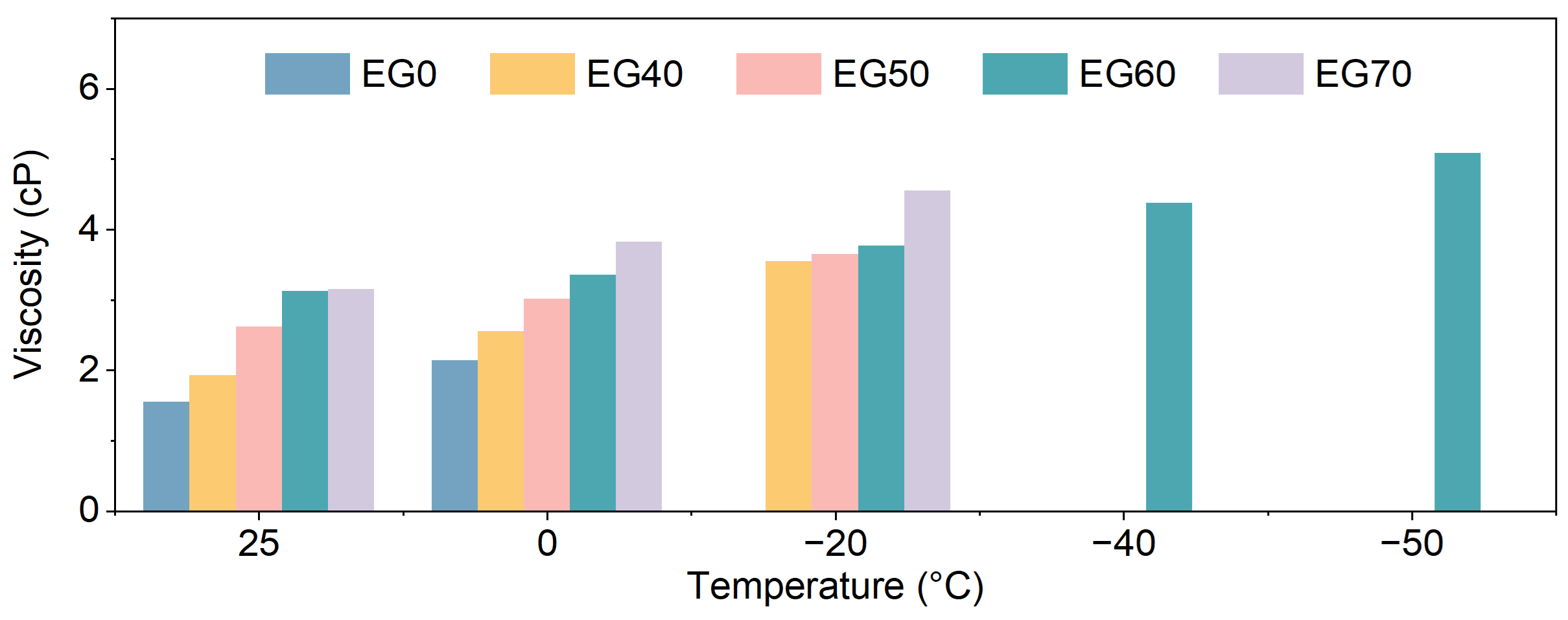


**Figure S26.** Viscosity plot of electrolytes with different composition ratios at various temperatures.


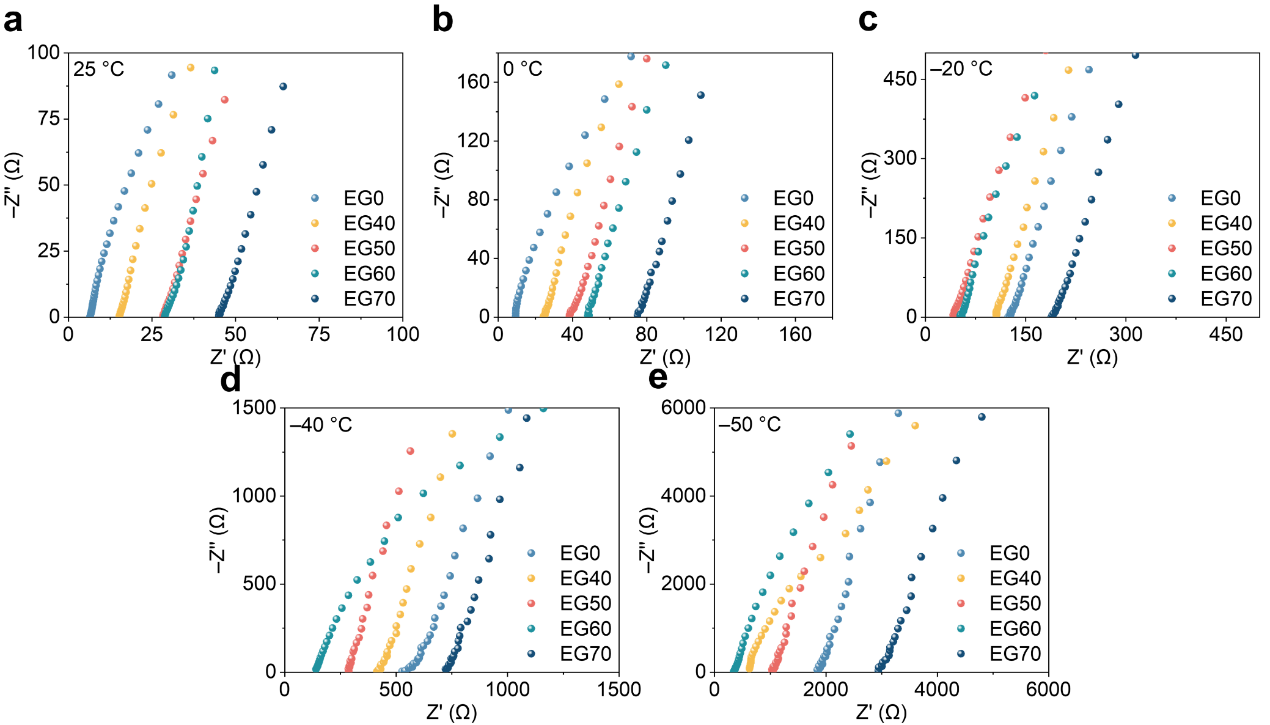


**Figure S27.** EIS plots of electrolytes with different composition ratios at various temperatures.


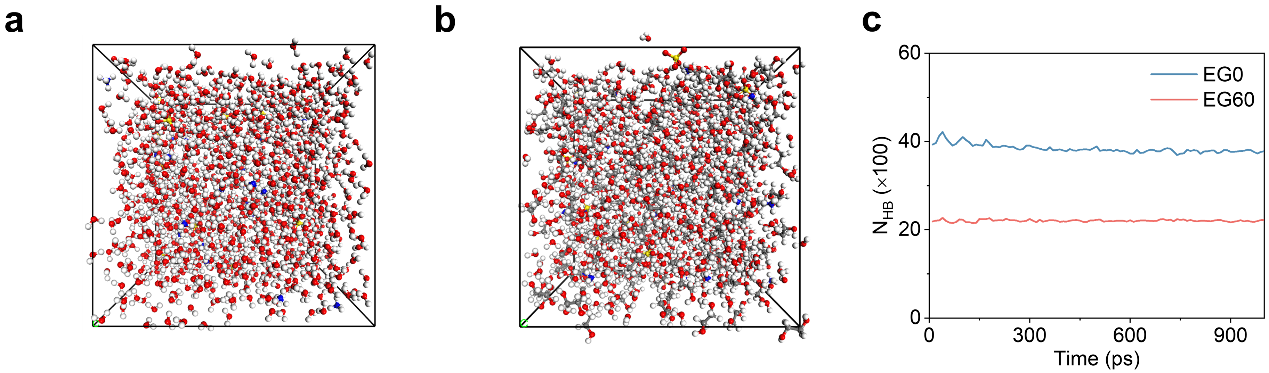


**Figure S28.** Snapshots of (a) EG0 and (b) EG60 at 25 °C. (c) Hydrogen-bond number of EG0 and EG60.


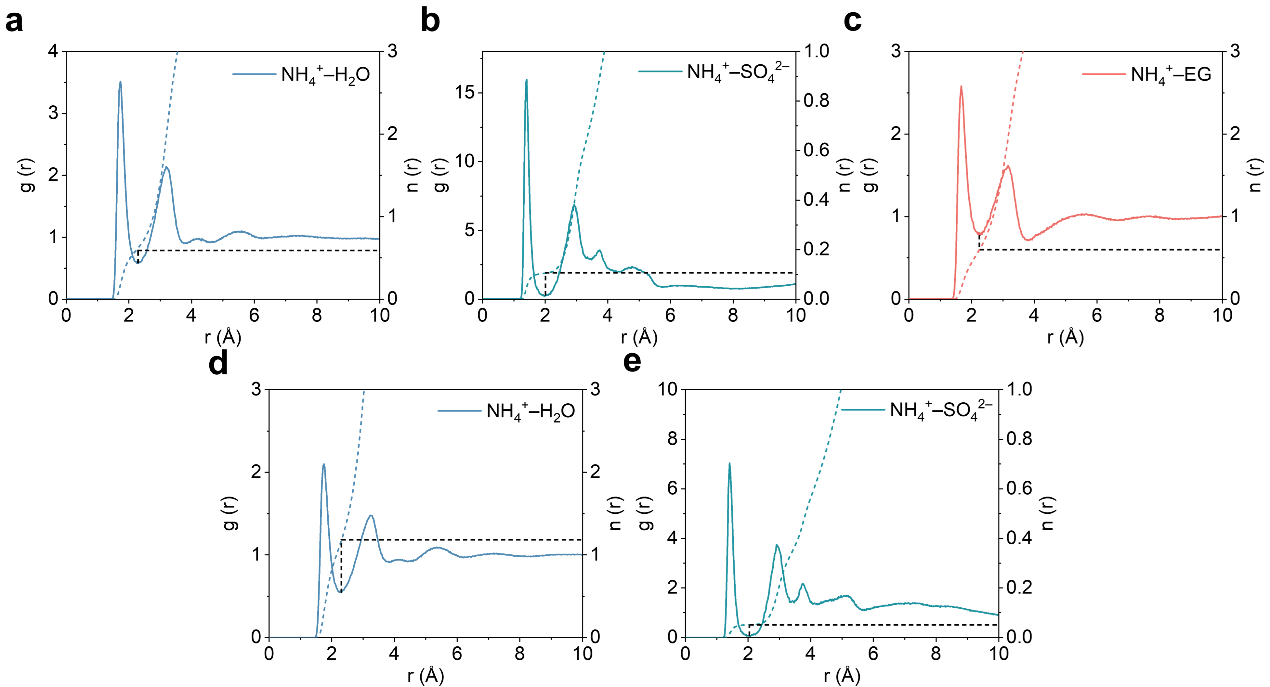


**Figure S29.** The radial distribution functions g(r) and coordination number distribution functions n(r) obtained from MD simulations for (a-c) EG60 and (d, e) EG0 at –50 °C, where r is radial distance.


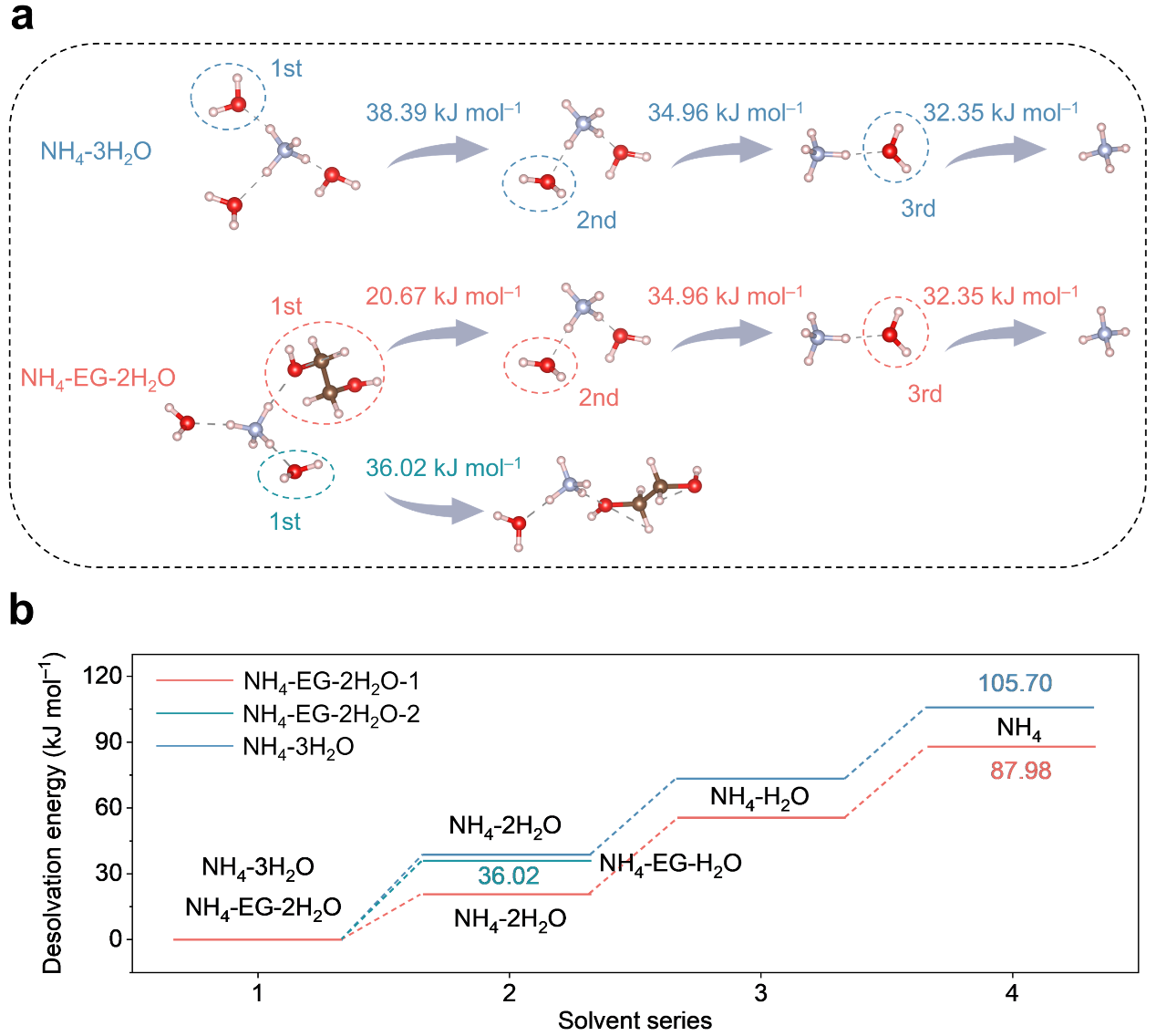


**Figure S30.** (a) The stepwise desolvation process and energy barriers. (b) The total desolvation energy calculation.


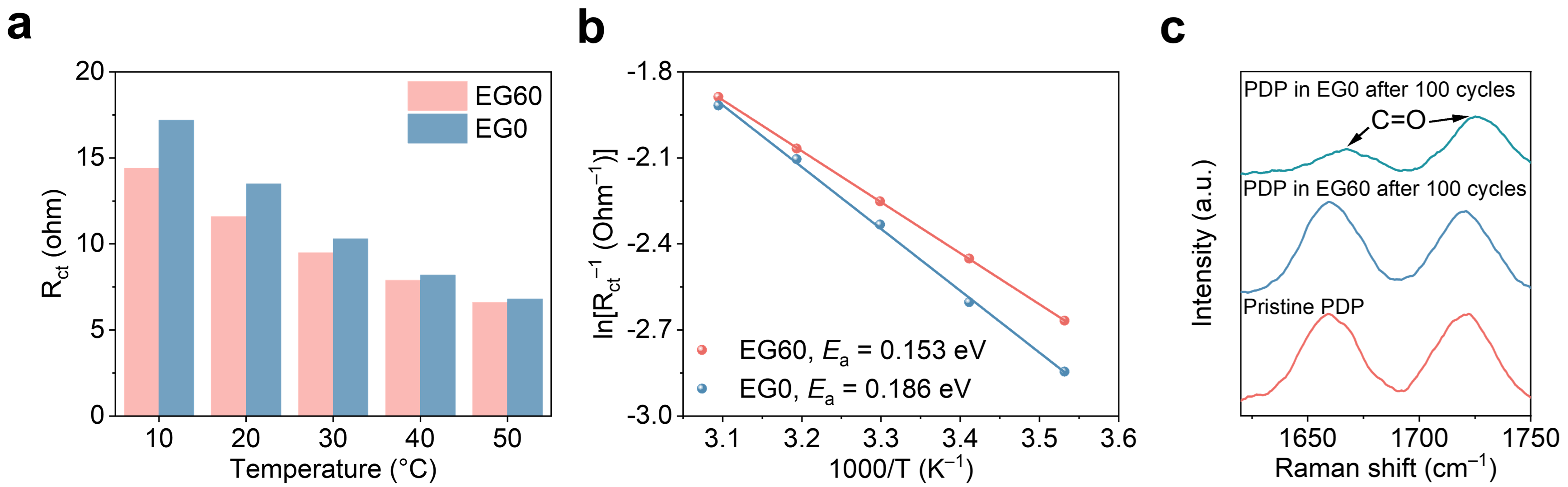


**Figure S31.** (a) R_ct_ values at different temperatures in EG60 and EG0. (b) Calculated E_a_ values.


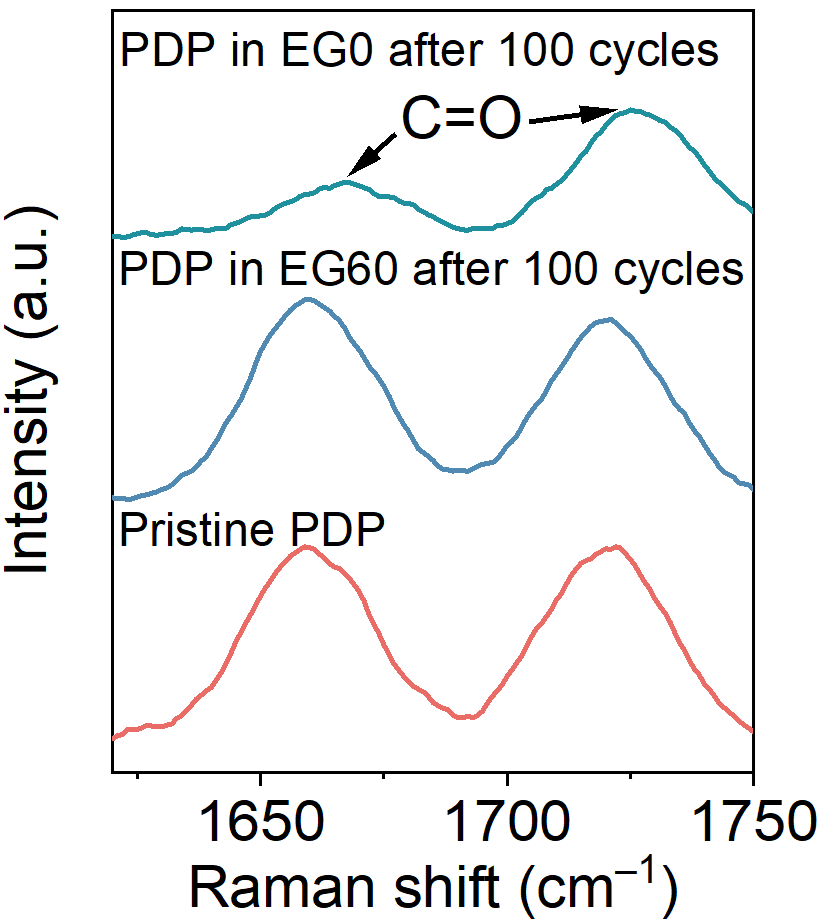


**Figure S32.** Raman spectra of Pristine PDP, PDP in EG60 after 100 cycles, and PDP in EG0 after 100 cycles.


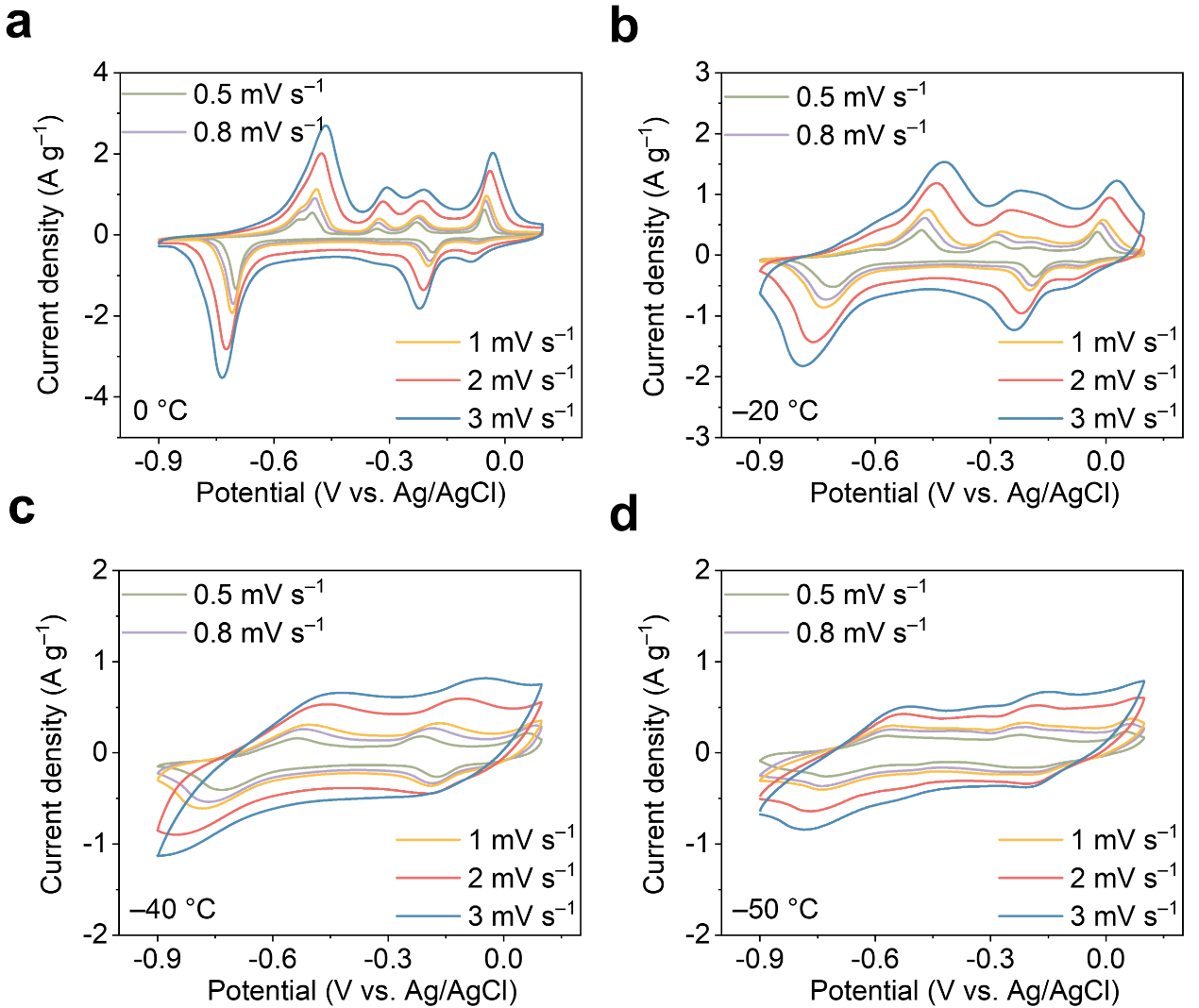


**Figure S33.** CV curves of PDP electrode at different scan rates under (a) 0 °C, (b) –20 °C, (c) –40 °C, and (d) –50 °C.


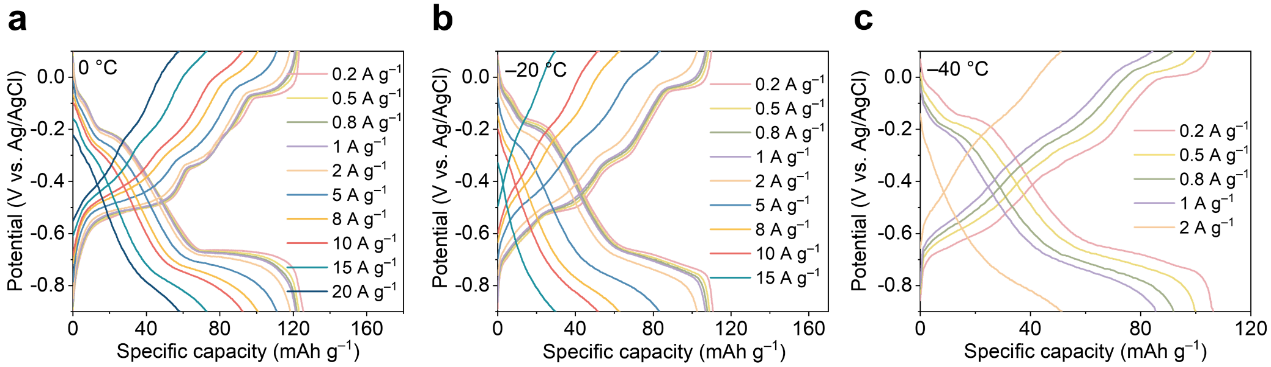


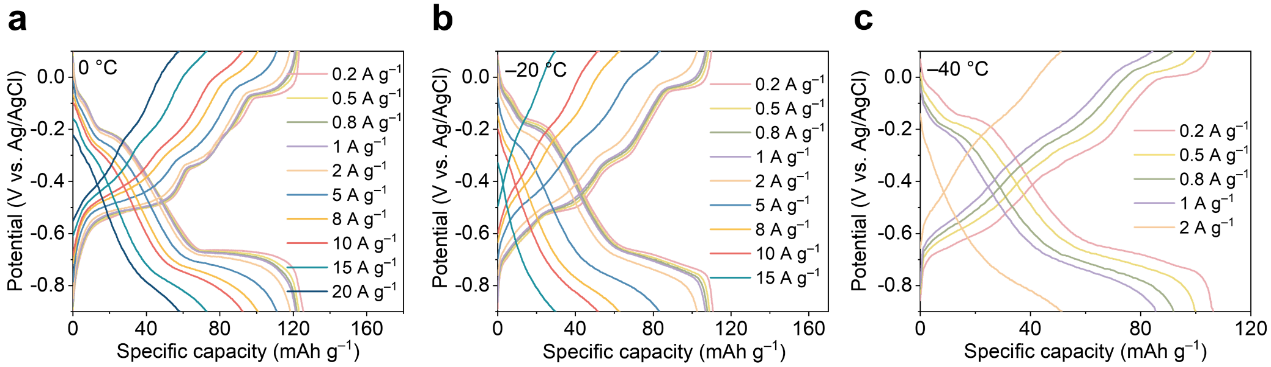


**Figure S34.** GCD curves of PDP electrode at different current densities under (a) 0 °C, (b) –20 °C, and (c) –40 °C.


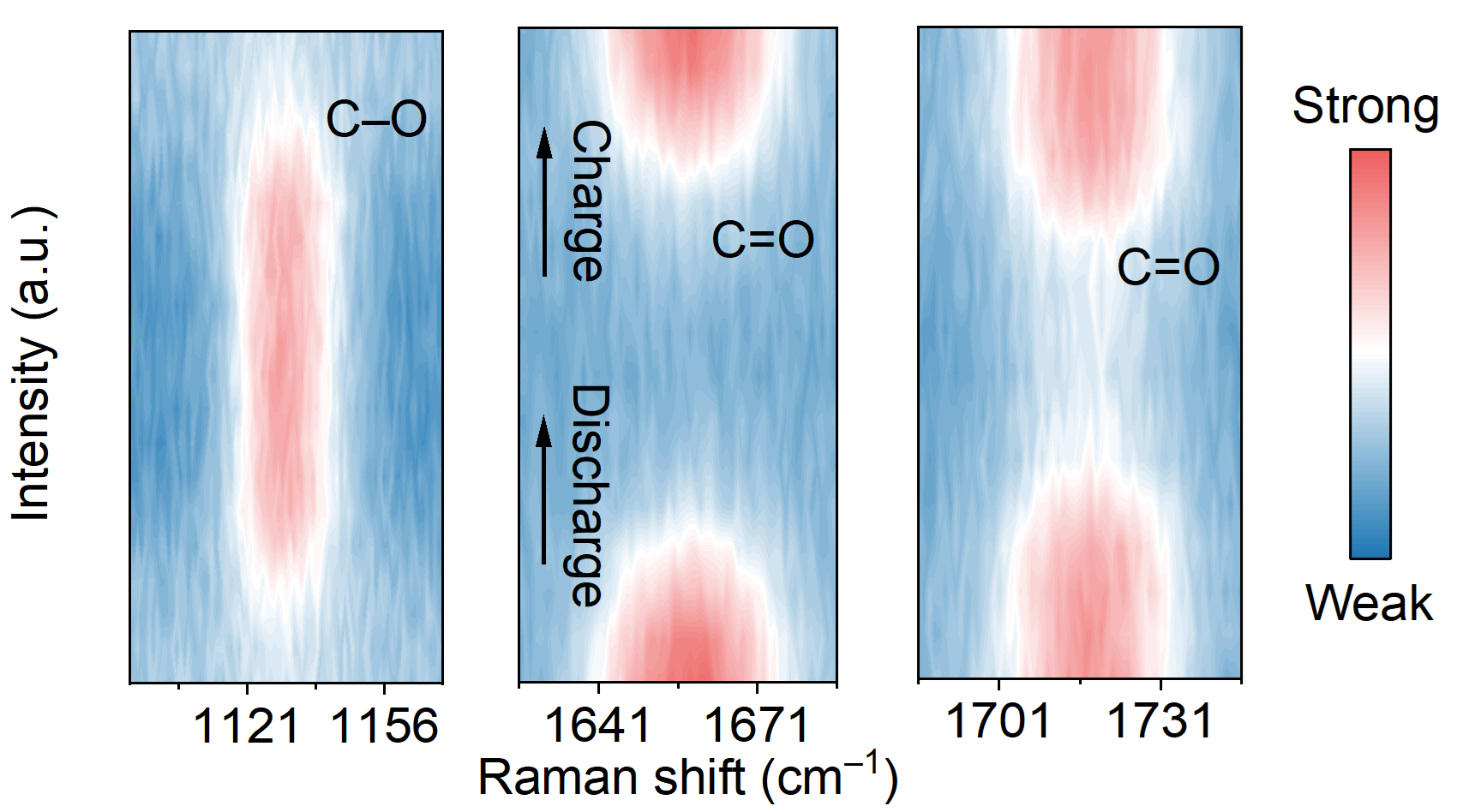


**Figure S35.** In-situ Raman spectra of PDP electrode at low temperature.


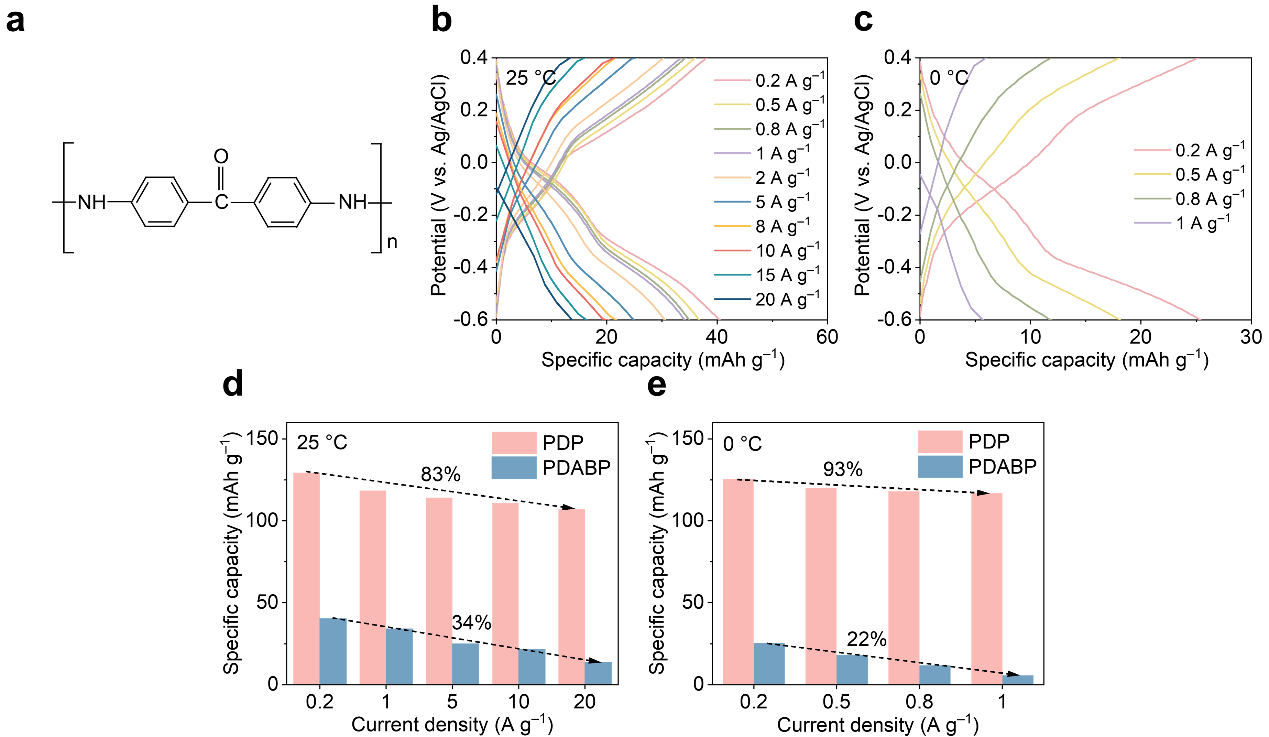


**Figure S36.** (a) The structural formula of PDABP. GCD curves of PDABP at different current densities under (b) 25 °C and (c) 0 °C. The comparison of capacity retention rates of PDP and PDABP at (d) 25 °C and (e) 0 °C.


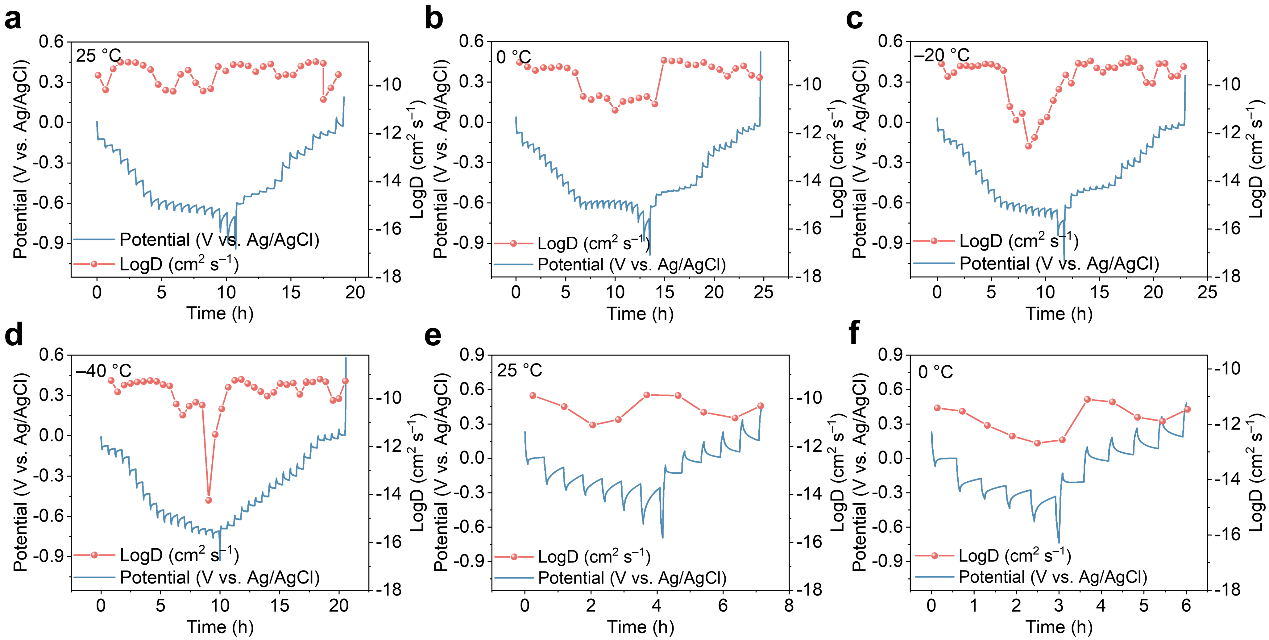


**Figure S37.** GITT curve and corresponding NH_4_^+^ diffusion coefficient of (a-d) PDP and (e-f) PDABP at different temperatures.


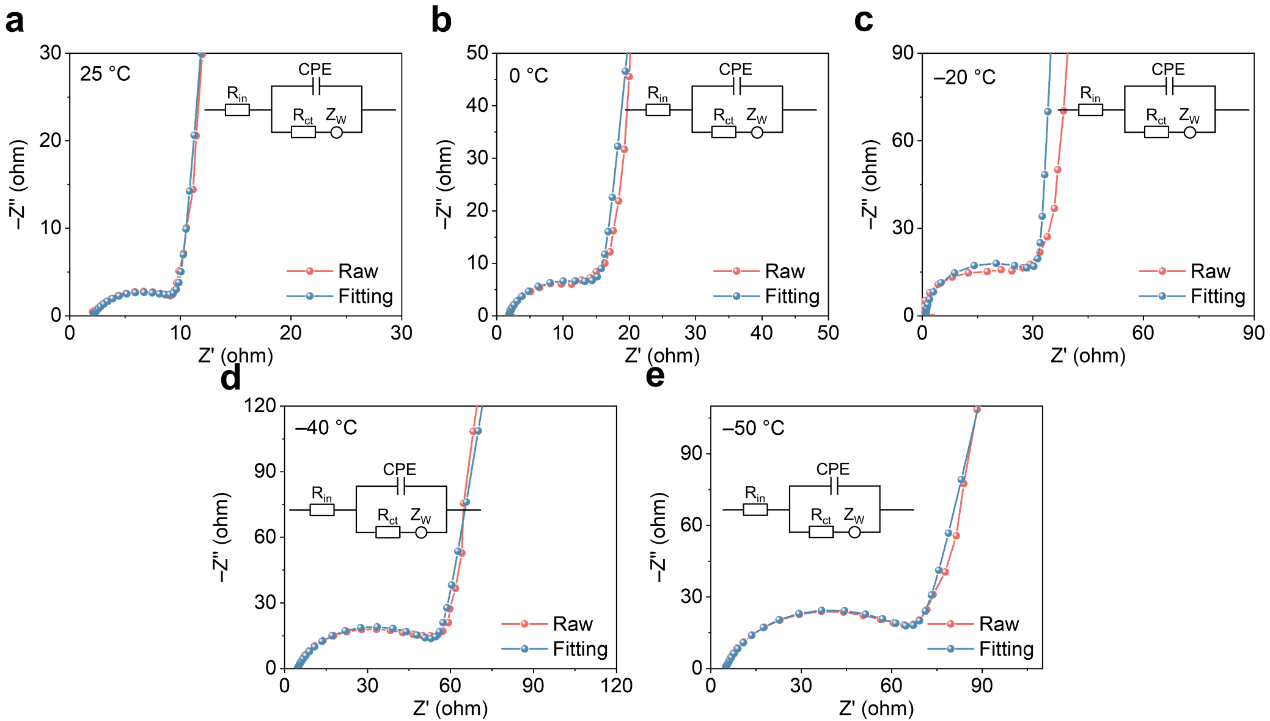


**Figure S38.** EIS plots of PDP electrode at different temperatures.


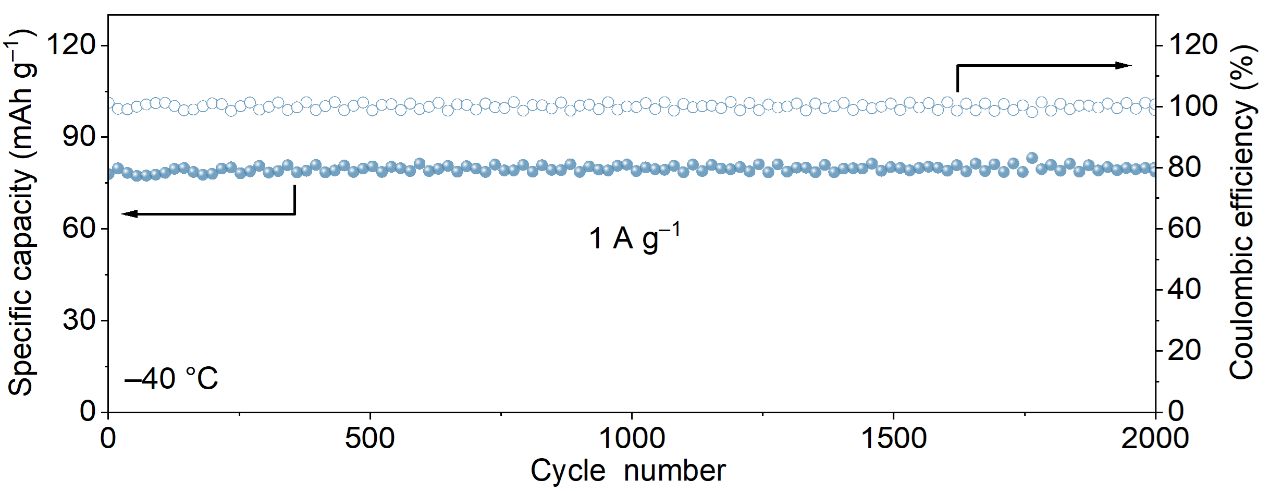


**Figure S39.** Cycling performance of PDP electrode at 1 A g^–1^ under –40 °C.


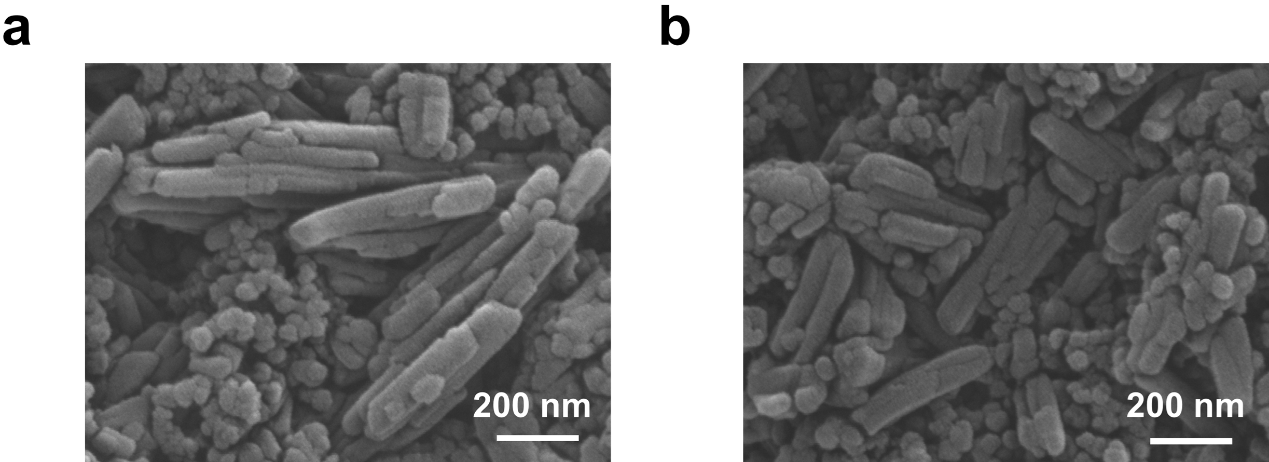


**Figure S40.** SEM images for PDP electrodes (a) before and (b) after 2000 cycles.


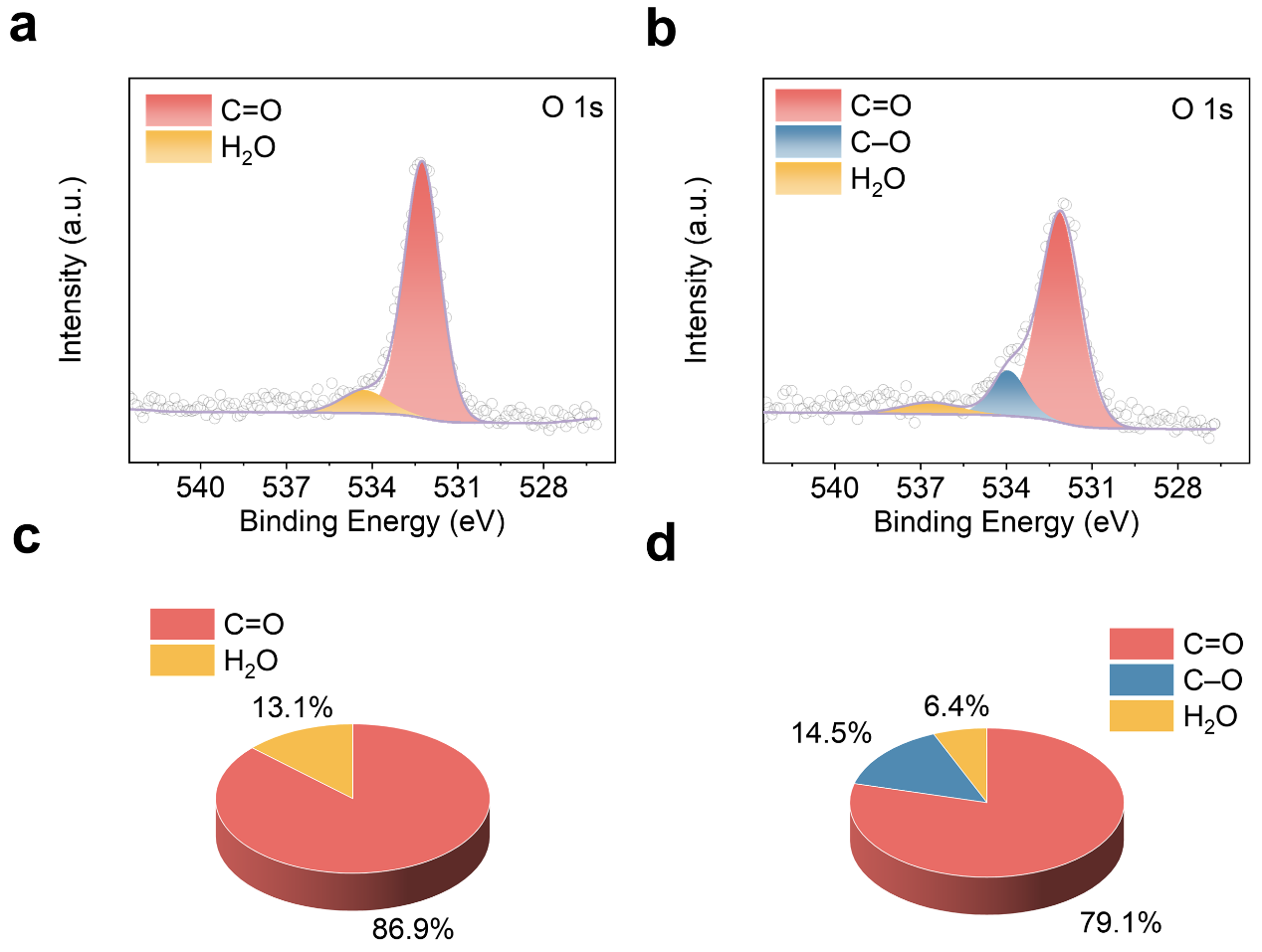


**Figure S41.** O 1s XPS spectra for PDP electrodes (a) before and (b) after 2000 cycles. (c-d) The percentage of element O in each part of (a) and (b).


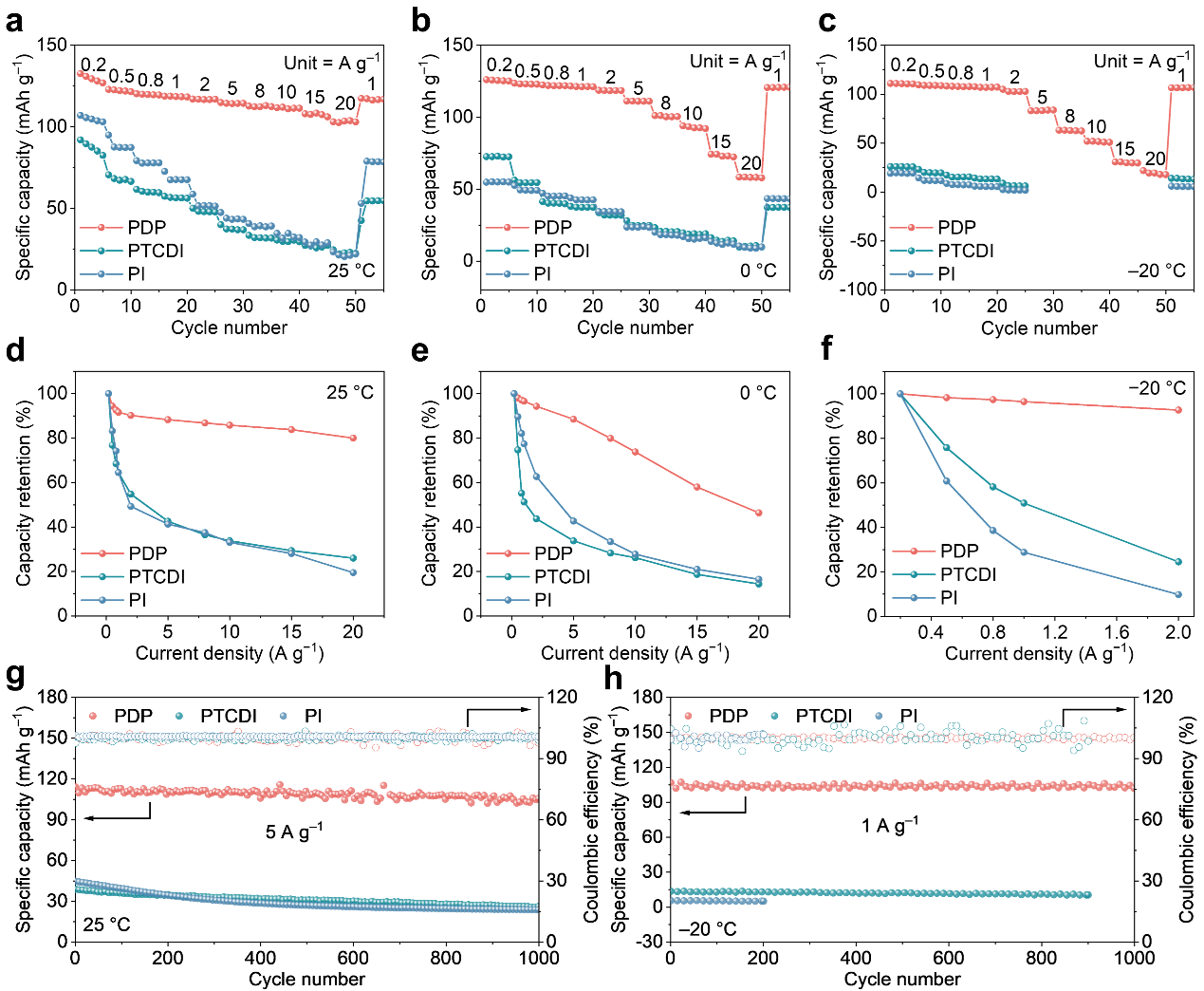


**Figure S42.** The comparison of rate performance of PDP, PTCDI, and PI at (a) 25 °C (b) 0 °C, and (c) –20 °C. The comparison of capacity retention of PDP, PTCDI, and PI at (d) 25 °C, (e) 0 °C, and (f) –20 °C. The comparison of cycle performance of PDP, PTCDI, and PI at (g) 25 °C and (h) –20 °C.


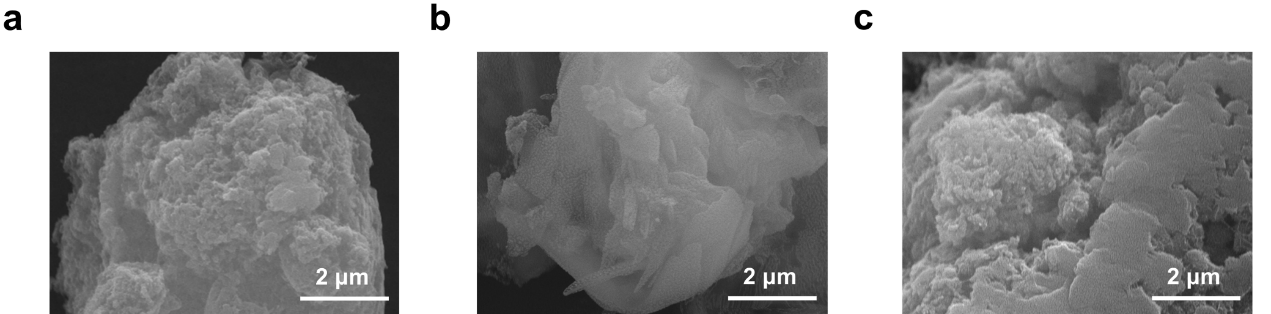


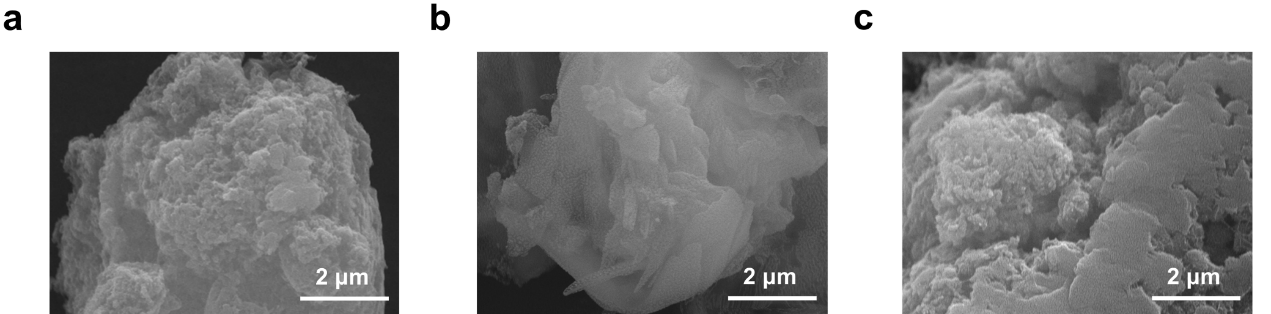


**Figure S43.** SEM images of (a) PANI, (b) rGO, and (c) PANI@rGO.


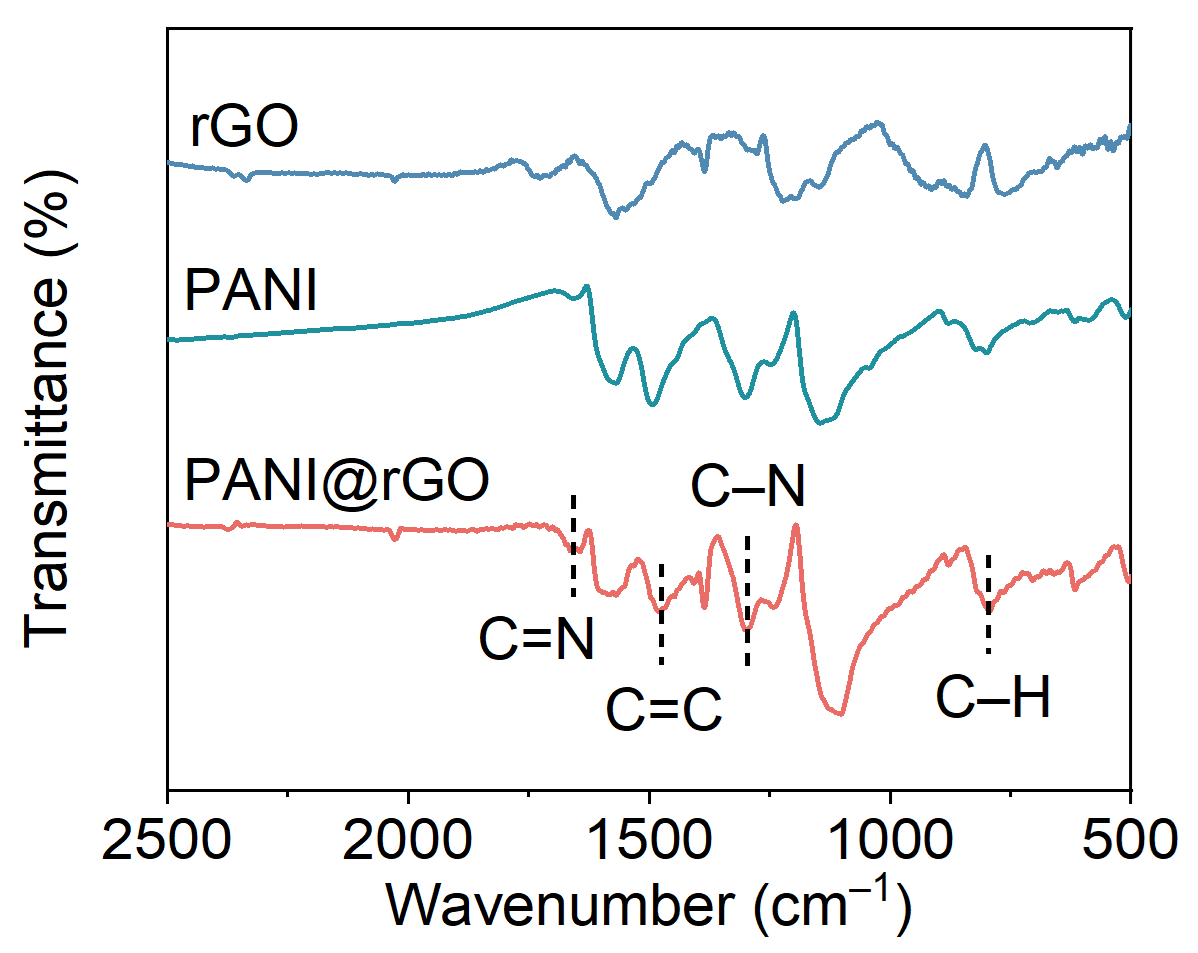


**Figure S44.** FTIR spectra of PANI@rGO, PANI, and rGO.


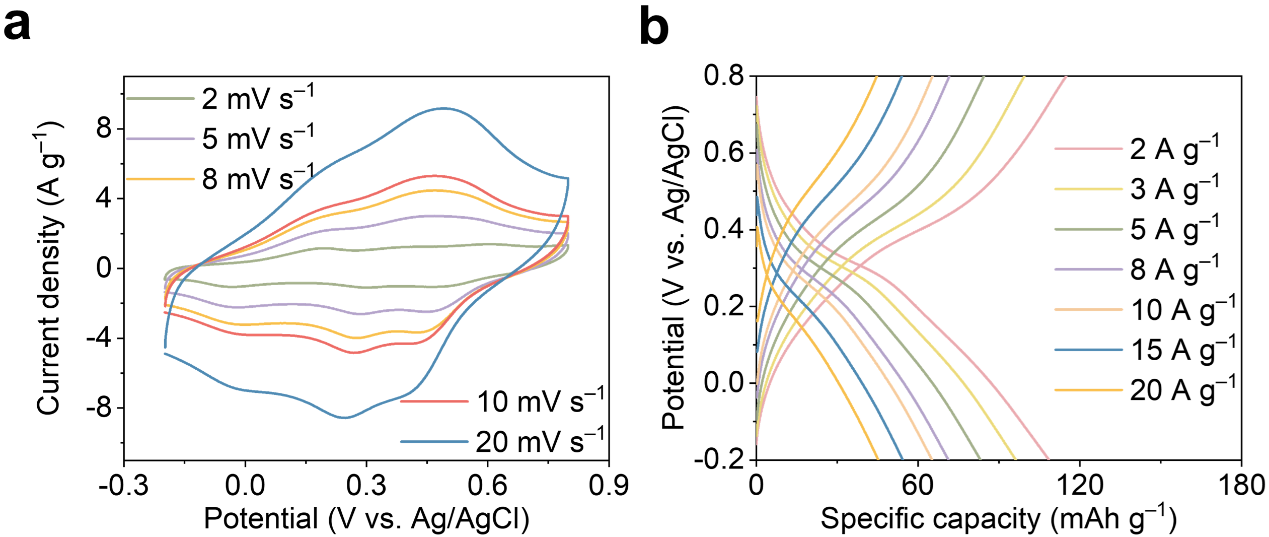


**Figure S45.** (a) CV curves of PANI@rGO at different scan rates. (b) GCD curves of PANI@rGO at different current densities.


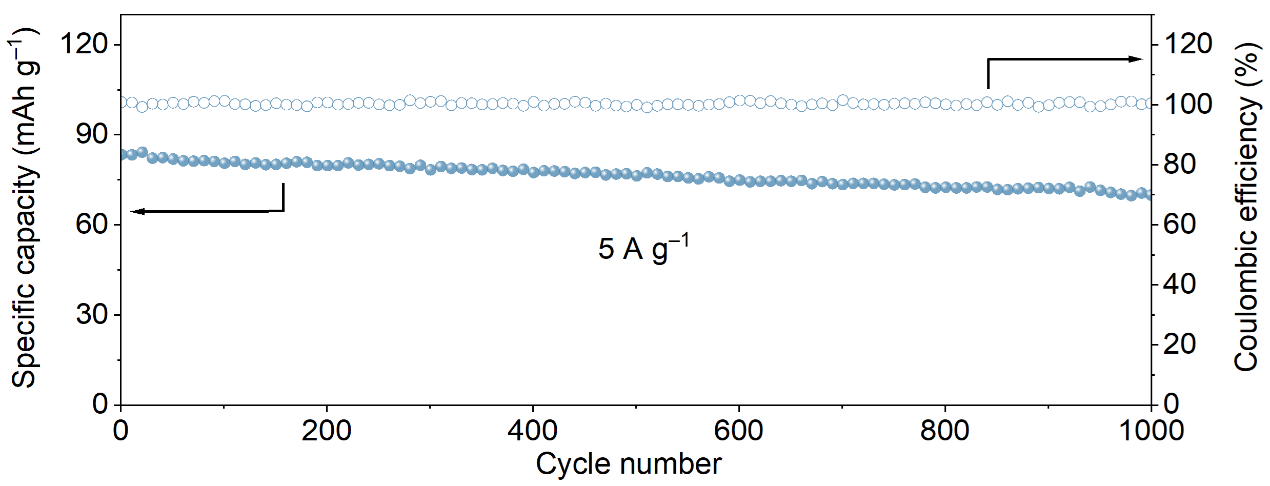


**Figure S46.** Cycling performance of PANI@rGO at 5 A g^–1^.


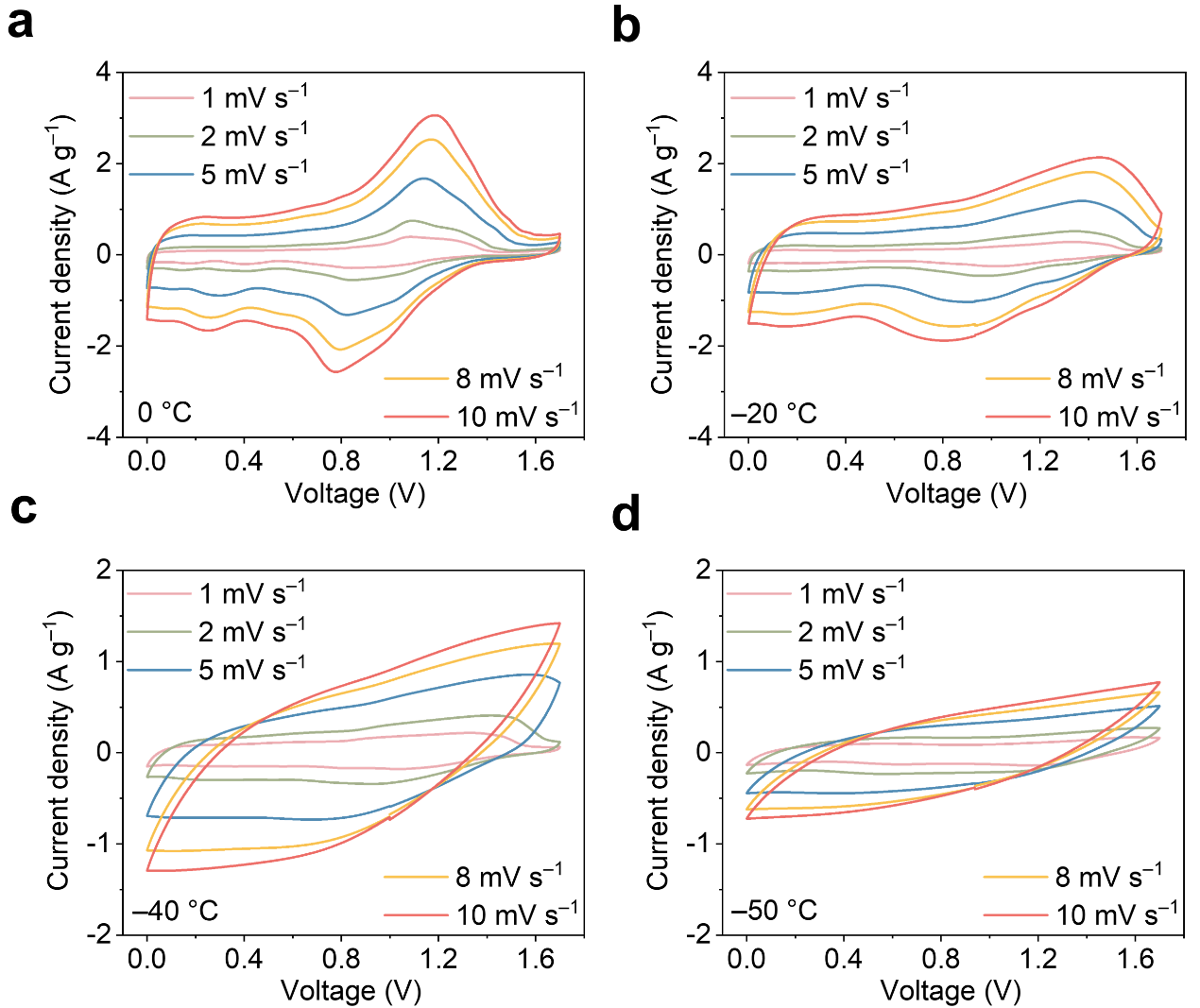


**Figure S47.** CV curves of the hybrid ammonium-ion capacitor at different scan rates under (a) 0 °C, (b) –20 °C, (c) –40 °C, and (d) –50 °C.


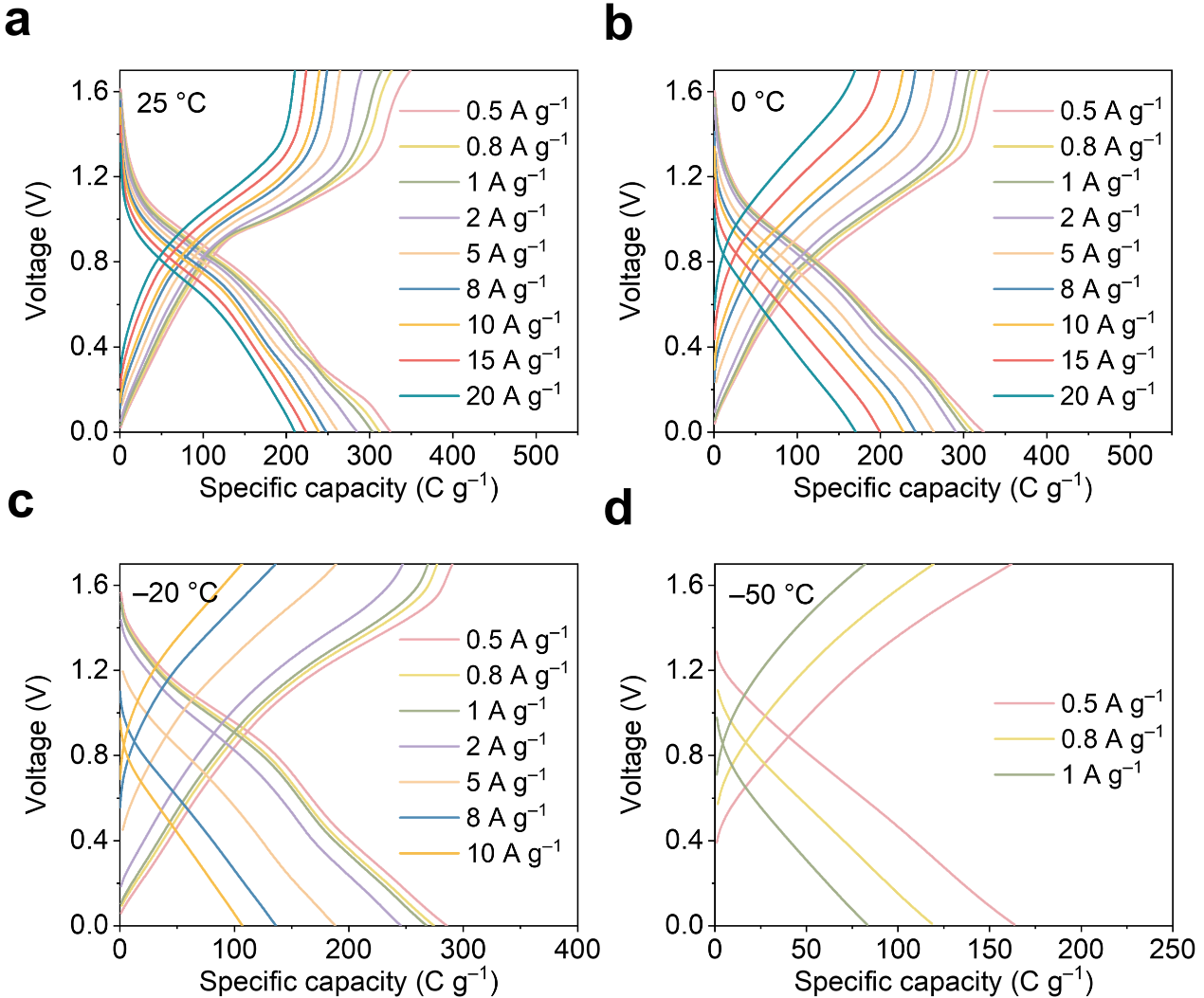


**Figure S48.** GCD curves of the hybrid ammonium-ion capacitor at different current densities under (a) 25 °C, (b) 0 °C, (c) –20 °C, and (d) –50 °C.


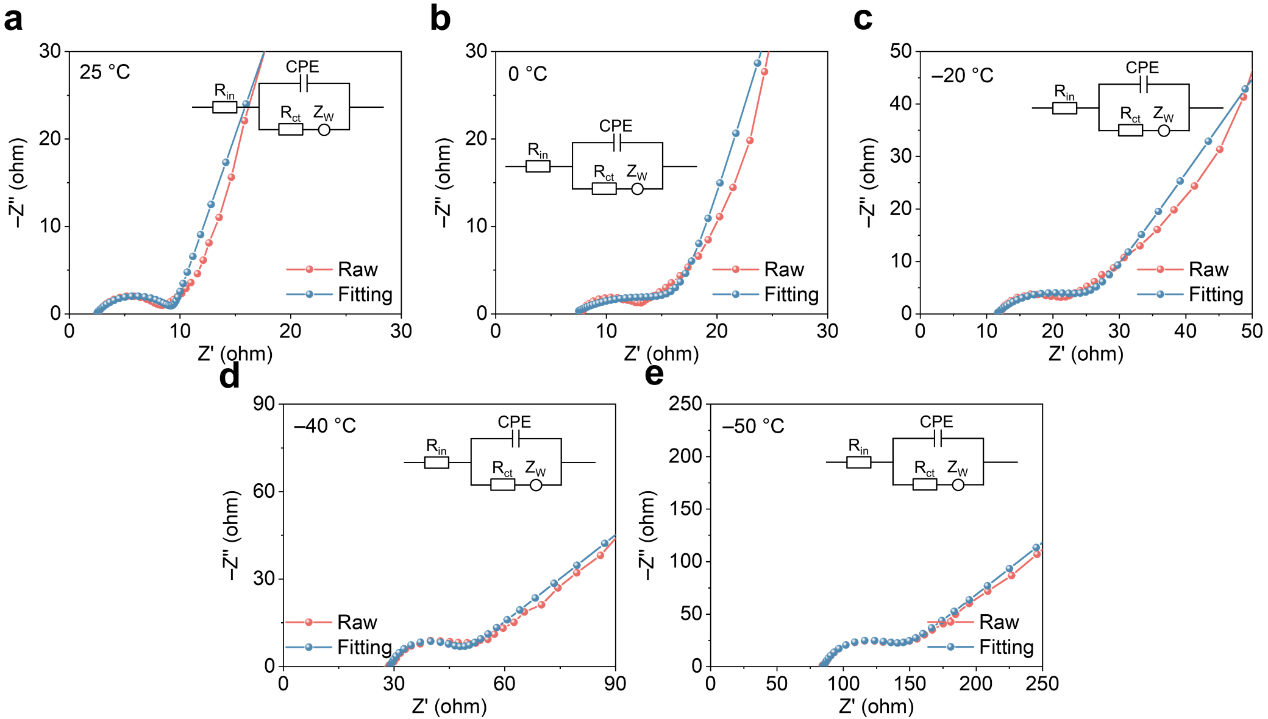


**Figure S49.** EIS plots of the hybrid ammonium-ion capacitor at different temperatures.


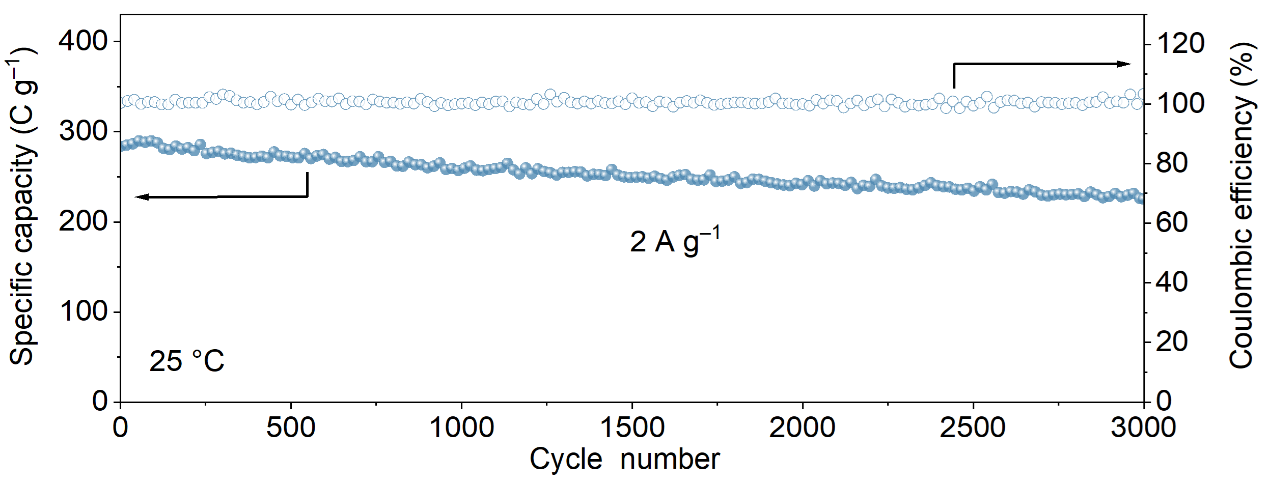


**Figure S50.** Cycling performance of the hybrid ammonium-ion capacitor at 2 A g^–1^ under 25 °C.

**
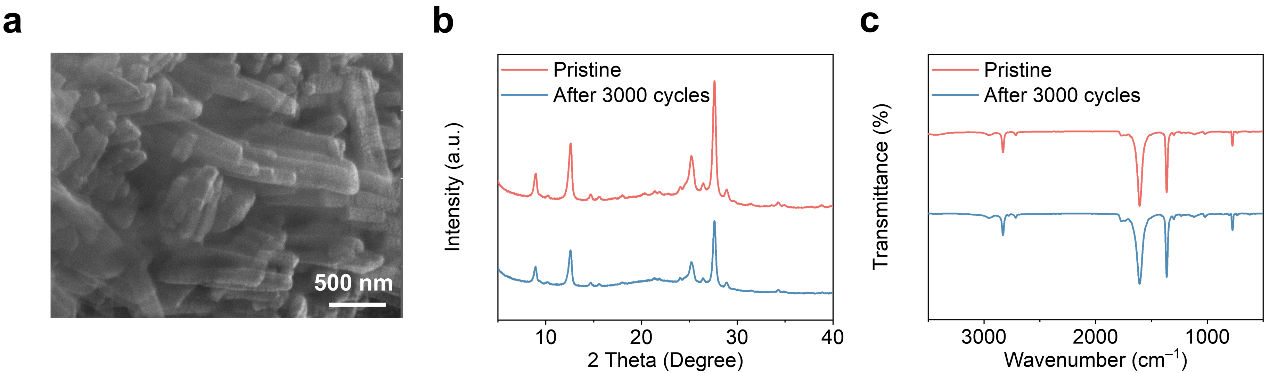
**

**Figure S51.** (a) SEM image for PDP electrode after 3000 cycles in the hybrid ammonium-ion capacitor. (b) XRD pattern and (c) FTIR spectra for PDP electrodes before and after 3000 cycles in the hybrid ammonium-ion capacitor.

**
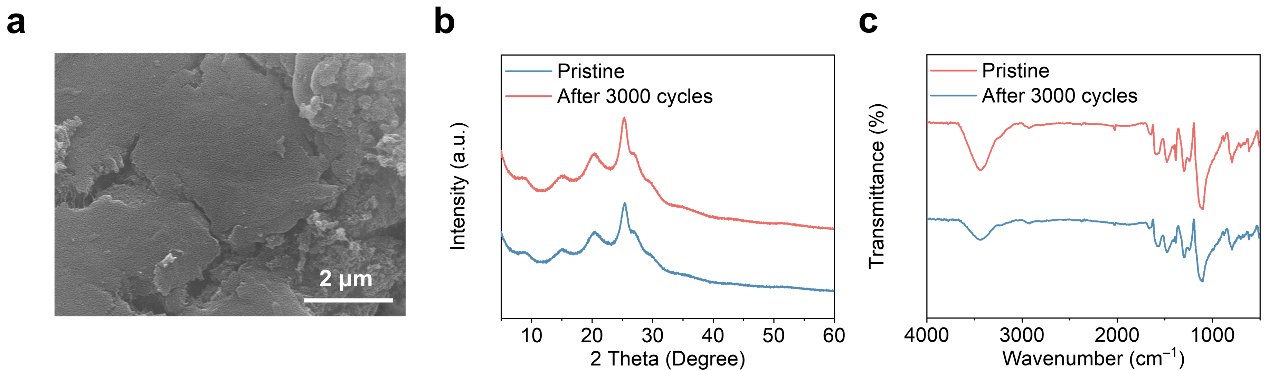
**

**Figure S52.** (a) SEM image for PANI@rGO electrode after 3000 cycles in the hybrid ammonium-ion capacitor. (b) XRD pattern and (c) FTIR spectra for PANI@rGO electrodes before and after 3000 cycles.

**
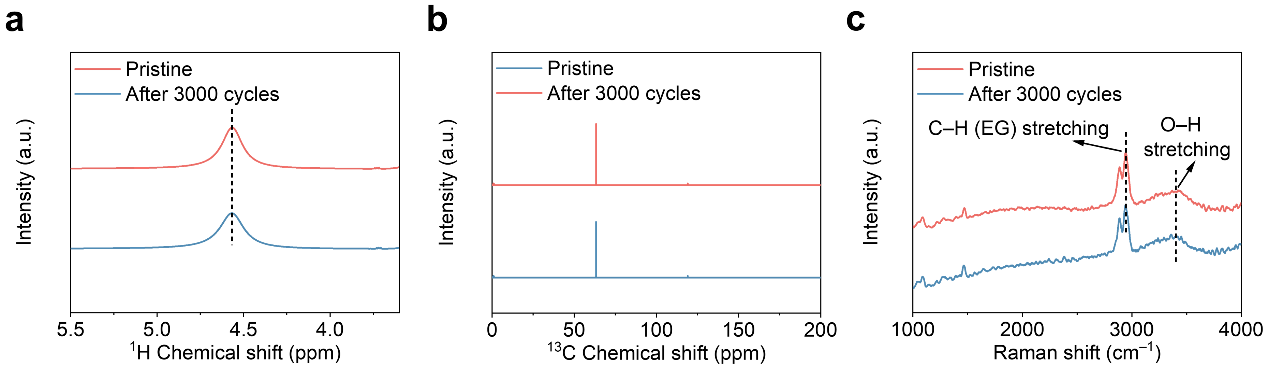
**

**Figure S53.** (a) ^1^H NMR spectra, (b) ^13^C NMR spectra, and (c) Raman spectra of EG60 in the hybrid ammonium-ion capacitor before and after 3000 cycles.

**
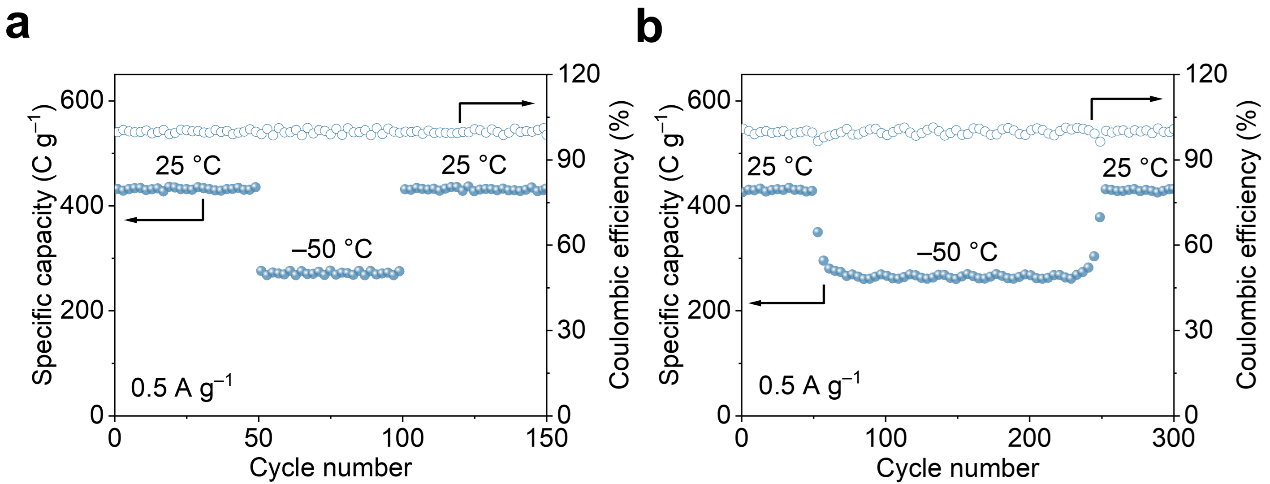
**

**Figure S54.** (a) Cycling stability and (b) temperature shock test under alternating temperatures between 25 °C and –50 °C.

**Table S1.** Comparison of rate performance for organic/inorganic electrode materials in ammonium-ion batteries/capacitors.

| Chemical formula | Rate performance  Specific capacity at Current density | Journal name | Ref. |
| --- | --- | --- | --- |
| PTCDI | 50 mAh g^–1^ at 3.6 A g^–1^ | Angew. Chem. Int. Ed. | [1] |
| PNNI | 111 mAh g^–1^ at 5 A g^–1^ | ACS Energy Lett. | [2] |
| QA-COF | 40 mAh g^–1^ at 10 A g^–1^ | J. Am. Chem. Soc. | [3] |
| TCNQ-rGO | 83 mAh g^–1^ at 10 A g^–1^ | J. Colloid Interface Sci. | [4] |
| Poly (1,5-NAPD) | 69 mAh g^–1^ at 20 A g^–1^ | eScience | [5] |
| PTCDA/Ti_3_C_2_T_x_ MXene | 91 mAh g^–1^ at 15 A g^–1^ | Adv. Sci. | [6] |
| PTPD | 97 mAh g^–1^ at 10 A g^–1^ | Adv. Funct. Mater. | [7] |
| DAAQ-TP-COF | 51 mAh g^–1^ at 10 A g^–1^ | Angew. Chem. Int. Ed. | [8] |
| MnHCF | 30 mAh g^–1^ at 0.5 A g^–1^ | Angew. Chem. Int. Ed. | [9] |
| Ni-APW | 22 mAh g^–1^ at 1.8 A g^–1^ | Angew. Chem. Int. Ed. | [1] |
| VO_x_@PPy | 107 mAh g^–1^ at 6 A g^–1^ | Chem. Eng. J. | [10] |
| E-VOPO | 48 mAh g^–1^ at 20 A g^–1^ | Adv. Mater. | [11] |
| NiHCF@CNTs | 15 mAh g^–1^ at 10 A g^–1^ | eScience | [5] |
| rGO_60_/MnO_x_ | 109 mAh g^–1^ at 5 A g^–1^ | Appl. Energy | [12] |
| WO_3_-1 | 53 mAh g^–1^ at 2 A g^–1^ | Chem. Eng. J. | [13] |
| VO_2_@C | 66 mAh g^–1^ at 5 A g^–1^ | J. Colloid Interface Sci. | [14] |
| MnAl-LDH | 70 mAh g^–1^ at 5 A g^–1^ | Adv. Energy Mater. | [15] |
| VOPO_4_·2H_2_O | 85 mAh g^–1^ at 1 A g^–1^ | Angew. Chem. Int. Ed. | [16] |
| P-NWO | 81 mAh g^–1^ at 2 A g^–1^ | Chem. Eng. J. | [17] |
| CuHCF | 50 mAh g^–1^ at 20 A g^–1^ | Chin. Chem. Lett. | [18] |
| BVO | 24 mAh g^–1^ at 5 A g^–1^ | Chin. Chem. Lett. | [18] |
| d-V_2_CT_x_ | 53 mAh g^–1^ at 5 A g^–1^ | Nat. Commun. | [19] |
| BG-2 | 60 mAh g^–1^ at 5 A g^–1^ | J. Colloid Interface Sci. | [20] |
| Mn-PBA@ppy | 39 mAh g^–1^ at 3 A g^–1^ | Adv. Energy Mater. | [21] |
| Bi_2_Se_3_NF | 84 mAh g^–1^ at 10 A g^–1^ | Adv. Funct. Mater. | [22] |
| NiHCF | 44 mAh g^–1^ at 1 A g^–1^ | Adv. Mater. | [23] |
| PDP | 107 mAh g^–1^ at 20 A g^–1^ | / | This work |

**Table S2.** Comparison of operating low temperature for ammonium-ion storage devices.

| Electrodes: Cathode//Anode | Electrolyte | Operating low temperature | Specific capacity (C g^–1^) | Ref. |
| --- | --- | --- | --- | --- |
| NiHCF@CNTs//Poly(1,5-NAPD) | 19 M CH_3_COONH_4_ | –40 °C | 190.8 at 1 A g^–1^ | [5] |
| Graphite//PTCDI | 1 m NH_4_PF_6_/AND-EMC (1: 1 by mass) | –20 °C | 179.3 at 0.2 A g^–1^ | [24] |
| Carbon//Carbon | 0.5 M NH_4_Cl  (50%-EDA) | –20 °C | 106.9 at 0.5 A g^–1^ | [25] |
| AC//PTCDI-rGO | 32 M CH_3_COONH_4_ | –20 °C | 49.0 at 1 A g^–1^ | [26] |
| PANI//PPy | 19 M CH_3_COONH_4_ | 0 °C | 68.4 at 1 A g^–1^ | [27] |
| PANI@rGO//PDP | 0.5 M (NH_4_)_2_SO_4_  (EG: H_2_O=6: 4) | –50 °C  –40 °C | 163.4 at 0.5 A g^–1^  207.4 at 1 A g^–1^ | This work |

**Table S3.** Comparison with the performance of lithium-ion batteries.

| Devices | Operating temperature range (°C) | Low-temperature cycling performance | Safety  (Electrolyte and corrosivity) | Ref. |
| --- | --- | --- | --- | --- |
| LFP//Graphite | –50~25 | 90.36% after 300 cycles (–30 °C) | 1.5 M LiFSI in 2MT: FEC (9:1 vol/vol)  Corrosion of the aluminum current collector and the electrode active material | [28] |
| NCM622//Graphite | –20~25 | 85.1% after 1000 cycles at 0.3 C (–20 °C) | 0.3 M LiF + 0.1 M TPFPB + 0.05 M LiDFOB in MA: FEC (9:1 vol/vol)  Corrosion of the aluminum current collector | [29] |
| NCM811//Graphite | –40~25 | 93.5% after 300 cycles at 0.5 C (–20 °C) | LiFSI: MB: EMC: TTE = 1:1.025:0.483:1.1  Flammable | [30] |
| LiFePO_4_//Graphite | –30~25 | 98% after 300 cycles at 1 C (–10 °C) | 0.55 M LiPF_6_ + 0.55 M LiFSI in EC: EMC: DMC: EP (3:1:1:5 vol/vol)  Corrosion of the aluminum current collector | [31] |
| LiCoO_2_//Graphite | –20~25 | 80.6% after 100 cycles (–20 °C) | 1 M LiPF_6_ in EC: DEC: DMC (1:1:1) Corrosion of the aluminum current collector | [32] |
| NCM811//Graphite | –20~25 | 96.2% after 4000 cycles at 0.5 C (–20 °C) | 3 M LiPF_6_ in EA/FEC (9:1 vol/vol)  Severe battery swelling and failure induced by EA | [33] |
| PANI@rGO//PDP | –50~25 | 97.5% after 3000 cycles at 0.5 A g^–1^ (–50 °C) | 0.5 M (NH_4_)_2_SO_4_ in EG: H_2_O (6:4 vol/vol)  Non-corrosive | This work |

**References**

[1] Wu X, Qi Y, Hong JJ, Li Z, Hernandez AS, Ji X. Rocking-chair ammonium-ion battery: a highly reversible aqueous energy storage system. *Angew. Chem. Int. Ed.* 2017; 56(42): 13026-13030.

[2] Zhang S, Zhu K, Gao Y, Cao D. A long cycle stability and high rate performance organic anode for rechargeable aqueous ammonium-ion battery. *ACS Energy Lett.* 2023; 8(2): 889-897.

[3] Tian Z, Kale VS, Wang Y, Kandambeth S, Czaban-Jóźwiak J, Shekhah O, Eddaoudi M, Alshareef HN. High-capacity NH_4_^+^ charge storage in covalent organic frameworks. *J. Am. Chem. Soc.* 2021; 143(45): 19178-19186.

[4] Shao P, Liao Y, Feng X, Yan C, Ye L, Yang J. Electronic modulation and structural engineering of tetracyanoquinodimethane with enhanced reaction kinetics for aqueous NH_4_^+^ storage. *J. Colloid Interface Sci.* 2023; 633: 199-206.

[5] Yan L, Qi Y, Dong X, Wang Y, Xia Y. Ammonium-ion batteries with a wide operating temperature window from −40 to 80 °C. *eScience*. 2021; 1(2): 212-218.

[6] Niu K, Shi J, Zhang L, Yue Y, Mo S, Li S, Li W, Wen L, Hou Y, Sun L, et al. MXene-integrated perylene anode with ultra-stable and fast ammonium-ion storage for aqueous micro batteries. *Adv. Sci.* 2023; 11(1): 2305524.

[7] Huang F, Zhao W, Guo Y, Mi Y, Gull S, Long G, Du P. Conjugated enhanced polyimide enables high-capacity ammonium ion storage. *Adv. Funct. Mater.* 2024; 34(44): 2407313.

[8] Liu J, Guo K, Guo W, Chang J, Li Y, Bao F. Superconjugated anthraquinone carbonyl-based covalent organic framework as anode material for high-performance aqueous ammonium-ion batteries. *Angew. Chem. Int. Ed.* 2025; 64(14): e202424494.

[9] Zhang H, Tian Y, Wang W, Jian Z, Chen W. Organic ammonium ion battery: a new strategy for a nonmetallic ion energy storage system. *Angew. Chem. Int. Ed.* 2022; 61(27): e202204351.

[10] Mu X, Song Y, Qin Z, Meng J, Wang Z, Liu XX. Core-shell structural vanadium oxide/polypyrrole anode for aqueous ammonium-ion batteries. *Chem. Eng. J.* 2023; 453(1): 139575.

[11] Zhang X, Wei H, Ren B, Jiang J, Qu G, Yang J, Chen G, Li H, Zhi C, Liu Z. Unlocking high-performance ammonium-ion batteries: activation of in-layer channels for enhanced ion storage and migration. *Adv. Mater.* 2023; 35(40): 2304209.

[12] Chen H, He H, Wang B, Han L, Ma J, Sui D, Wang C, Hua Y. Investigating the physical-chemical effects of reduced graphene oxide-covered manganese oxide on ammonium-ion batteries. *Appl. Energy*. 2024; 353: 122067.

[13] Wen X, Luo J, Xiang K, Zhou W, Zhang C, Chen H. High-performance monoclinic WO_3_ nanospheres with the novel NH_4_^+^ diffusion behaviors for aqueous ammonium-ion batteries. *Chem. Eng. J.* 2023; 458: 141381.

[14] Tan X, Zhang F, Chen D, Gong Jn, Sun J, Meng C, Zhang Y. One-step hydrothermal synthesis of vanadium dioxide/carbon core-shell composite with improved ammonium ion storage for aqueous ammonium-ion battery. *J. Colloid Interface Sci.* 2024; 669: 2-13.

[15] Liu Q, Ye F, Guan K, Yang Y, Dong H, Wu Y, Tang Z, Hu L. MnAl layered double hydroxides: a robust host for aqueous ammonium-ion storage with stable plateau and high capacity. *Adv. Energy Mater.* 2022; 13(5): 2202908.

[16] Ye F, Pang R, Lu C, Liu Q, Wu Y, Ma R, Hu L. Reversible ammonium ion intercalation/de-intercalation with crystal water promotion effect in layered VOPO_4_⋅2H_2_O. *Angew. Chem. Int. Ed.* 2023; 62(24): 2303480.

[17] Tian G, Ling D, Zhang D, Wang Q. Reversible NH_4_^+^ intercalation/de-intercalation with phosphate promotion effect in tunnel (NH_4_)_0.25_WO_3_. *Chem. Eng. J.* 2024; 482: 149160.

[18] Wang T, Li X, Zhao S, Bu H, Li C, Li N, Zhang X, Xu X. Rocking-chair ammonium ion battery with high rate and long-cycle life. *Chin. Chem. Lett.* 2024; 35(1): 108449.

[19] Bao Z, Lu C, Liu Q, Ye F, Li W, Zhou Y, Pan L, Duan L, Tang H, Wu Y, et al. An acetate electrolyte for enhanced pseudocapacitve capacity in aqueous ammonium ion batteries. *Nat. Commun.* 2024; 15(1): 1934.

[20] Guo Y, Qu J, Liu X, Wang P, Liu Z, Zhang J, Yi T. Berlin Green with tunable iron content as ultra-high rate host for efficient aqueous ammonium ion storage. *J. Colloid Interface Sci.* 2024; 667: 607-616.

[21] Liu Q, Zhang D, Yang Y, Gu Y, Liang Z, Chen W, Wu Y, Hu L. Encapsulation of prussian blue analogues with conductive polymers for high-performance ammonium-ion storage. *Adv. Energy Mater.* 2024; 15(4): 2402863.

[22] Long B, Ma X, Chen L, Song T, Pei Y, Wang X, Wu X. Se vacancy activated Bi_2_Se_3_ nanodots encapsulated in porous carbon nanofibers for aqueous zinc and ammonium ion batteries. *Adv. Funct. Mater.* 2024; 34(48): 2411430.

[23] Zhou M, Wu T, Kang M, Cheng T, Li H, He L, Lian C, Ma T, Zhao Q. Ligand field-induced dual active sites enhance redox potential of nickel hexacyanoferrate for ammonium ion storage. *Adv. Mater.* 2025; 37(32): 2419446.

[24] Zhao Z, Lei Y, Shi L, Tian Z, Hedhili MN, Khan Y, Alshareef HN. A 2.75 V ammonium-based dual-ion battery. *Angew. Chem. Int. Ed.* 2022; 61(51): 2212941.

[25] Ouyang D, Yang L, Chen D, Yin J, Li Y, Zhu H, Yu F, Yin J. Ethylenediamine modulate bonding interaction of solvation structure for wide-temperature aqueous ammonium-ion capacitor. *J. Colloid Interface Sci.* 2024; 663: 1028-1034.

[26] Tsai HH, Lin TJ, Vedhanarayanan B, Tsai CC, Chen TY, Ji X, Lin TW. A 1.9 V all-organic battery-supercapacitor hybrid device with high rate capability and wide temperature tolerance in a metal-free water-in-salt electrolyte. *J. Colloid Interface Sci.* 2022; 612: 76-87.

[27] Kuchena SF, Wang Y. A metal-free all-organic ammonium-ion battery with low-temperature applications. *J. Mater. Chem. A*. 2023; 11(6): 2814-2825.

[28] Wang Z, Gu X, Zhu J, Zhong C, Xu S, Weng S, Liu B, Wang Z, Li Y, Cheng T, Wang X. Weakly-solvated and Co-intercalation-free ether-based electrolytes enhance the low-temperature and fast-charging performance of LiFePO_4_||Graphite Batteries. *Angew. Chem. Int. Ed.* 2025; 14(13): e21171.

[29] Hossain M, Wu D, Zhu Q, Liu Q, Yang J, Xu J, Liu C, Kang S, Yang Z, Son S, Yoo D, Zhang Z. High Li^+^ transference number electrolyte enabled by fluoride acceptor for low temperature Li-ion batteries. *ACS Energy Lett.* 2025; 10(7): 3629-3637.

[30] Su F, Lin Y, Dou X, You H, Gao S, Bai Z, Jiang J, Chen L, Li C. Compact ion-pair aggregates dominated electrolytes enable high-performance low-temperature lithium-ion batteries. *Angew. Chem. Int. Ed.* 2025; 64(38): e202510647.

[31] Ma C, Qiu Z, Shan B, Song Y, Zheng R, Feng W, Cui Y, Xing W. The optimization of the electrolyte for low temperature LiFePO_4_-graphite battery. *Mater. Lett.* 2024; 356: 135594.

[32] Kim C, Jekal S, Kin J, Kin H, Park G, Ra Y, Noh J, Yoon C. Ester-based electrolytes for graphite solid electrolyte interface layer stabilization and low-temperature performance in lithium-ion batteries. *Carbon Lett.* 2024; 34(8): 2113-2125.

[33] Li Z, Yao N, Yu L, Yao Y, Jin C, Yang Y, Xiao Y, Yue X, Cai W, Xu L, Wu P, Yan C, Zhang Q. Inhibiting gas generation to achieve ultralong-lifespan lithium-ion batteries at low temperatures. *Matter* 2023; 6(7): 2274-2292.
